# Donor–Acceptor Separation Augments Temperature Dependence of Kinetic Isotope Effects in NADH Model Hydride Transfer Reactions: Mimicking Enzyme versus Mutant Dynamics

**DOI:** 10.1021/acs.jpcb.6c02719

**Published:** 2026-07-07

**Authors:** Nicholas DeGroot, Pratichhya Adhikari, Bibesh Pokhrel, Grishma Singh, Yun Lu

**Affiliations:** Department of Chemistry, 33140 Southern Illinois University Edwardsville, Edwardsville, Illinois 62026, United States

## Abstract

Observed shift from temperature (T)-independent hydrogen tunneling kinetic isotope effects (KIEs) in enzymes to T-dependent KIEs in mutants has been attributed to the donor­(D)–acceptor­(A) separation effects caused by the weakened protein dynamical compression of D–A distances (DADs) in mutants. To examine the relationship between D–A separations (DADs) and T-dependence of KIEs (represented by Δ*E*_a_ = *E*_aD_ – *E*_aH_), we design hydride-transfer reactions in solution. Our hypothesis is that a looser nucleus-transfer system exhibits a larger Δ*E*_a_ value. Herein, the Δ*E*_a_’s were determined for three series of apparent hydride-transfer reactions of NADH models in acetonitrile. These include hydride-transfers (1) from Hantzsch ester to 10-methyl-9-substituted­(R)­acridinium ions (RMA^+^), (2) from the reduced RMA^+^ (RMAH) to a benzoquinone structure, and (3) from RMAH to the [(BnTPEN)­Fe­(IV)=O]^2+^ complex. Reactions (2) and (3) use multistep electron–proton–electron sequential transfer mechanisms. Δ*E*_a_ increases from reactions (1) (0.94–1.19 kcal/mol) to (2) (1.14–1.60 kcal/mol) to (3) (3.05–5.05 kcal/mol), and within each series, Δ*E*_a_ increases with the size of the R substituent. The unusually high Δ*E*_a_’s observed for the iron­(IV)-oxo complex reactions are likely partly attributed to electrostatic repulsion between like-charged RMAH^+•^ acid and [(BnTPEN)­Fe­(III)=O]^+^ in the reaction complex. These results support our hypothesis and the proposed role of protein dynamics in barrier compression for enzyme catalysis.

## Graphical abstract

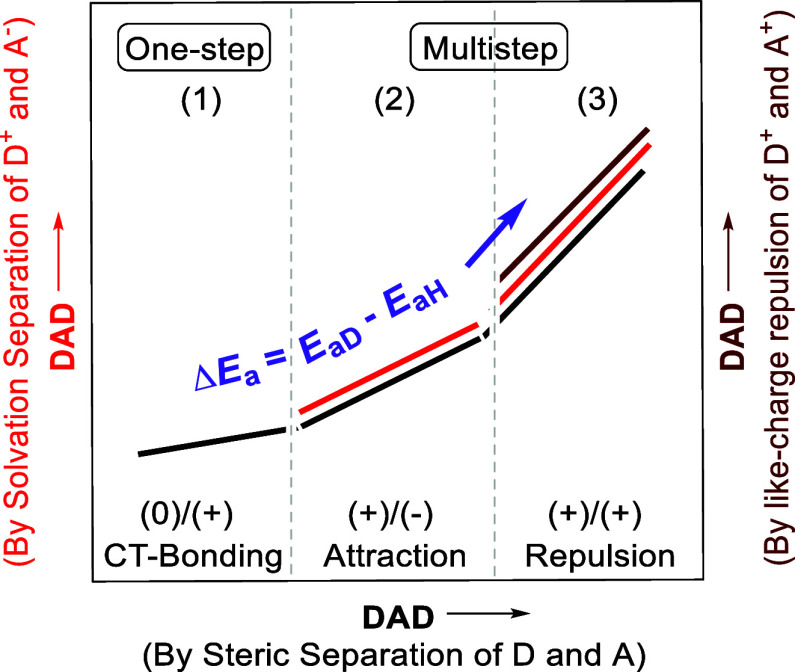

## Introduction

Kinetic isotope effect (KIE) is an important tool to determine mechanisms of the H-transfer reactions. Within the semiclassical transition state (TS) theory, KIE arises from differences in zero-point energies between isotopes at the reactant state and the TS. The deuterium (H/D) KIE is typically smaller than 7, and the isotopic activation energy difference (Δ*E*_a_ = *E*_aD_ – *E*_aH_), which reflects the temperature (T) dependence of KIEs, falls in the range of 1.0–1.2 kcal/mol. Observations outside of these semiclassical limits have been attributed to H-tunneling, in which H traverses the energy barrier via its wave-like properties. The earliest H-tunneling model was formulated by Bell who added a tunnel correction to the one-dimensional energy barrier.^1,2^ In the Bell model, KIE is largest at extremely low temperature conditions where *E*_aH_ and *E*_aD_ approach zero and Δ*E*_a_ ∼0.

In the recent two to three decades, however, the T-independence of both small and large KIEs (Δ*E*_a_ ∼0, but *E*_aH_ and *E*_aD_ > 0) has been frequently observed in wild-type enzyme-catalyzed H-transfer reactions at biological temperature conditions.^3–13^ While this has already been inexplainable by the Bell model, a further complication is that Δ*E*_a_ is found to increase, into the semiclassical range and beyond the 1.2 kcal/mol limit, as enzymes are mutated strategically to increase the donor–acceptor distance (DAD).^11,12,14–17^ Some researchers proposed the vibration-assisted activated H-tunneling (VA-AHT) model to explain the observed trend in Δ*E*_a_’s.^7,14,18–22^ The latter tunneling model involves two orthogonal activation processes. In one process, heavy atoms’ motions bring the reactants (Donor­(D) and Acceptor­(A)) to a tunneling-ready state (TRS) where the reactant [D-H A]^‡^ and product [D H-A]^‡^ moieties are degenerate for the H- wave function to effectively overlap, i.e., H-tunneling. In the other, heavy atom motions sample the short DADs for H-tunneling to occur efficiently. Given that the TRS formation step is H-isotope insensitive, KIE arises from the latter activation process. Since the wave function of H is more diffuse than D, the H-wave function overlap is more than the D-overlap, so that KIE >1. Meanwhile, since the D-overlap decreases with increasing DAD more rapidly than the H-overlap, KIE increases as DAD increases. Therefore, under this model, the T-independence of KIEs observed in wild-type enzymes has been ascribed to the strong protein dynamics (strong heavy atom motions) that press the donor and acceptor closer to each other so that the short DAD sampling is not influenced by temperature, so are the KIEs. When enzyme is mutated, however, the kinds of protein dynamics are impaired and DAD becomes longer so that the DAD and thus the KIE become T-dependent. Therefore, T-dependence of KIEs has been used to investigate the role of fast protein dynamics, also called the promoting vibrations,^23,24^ in enzyme catalysis.

The QM and hybrid QM/MM simulations of the T-dependence of KIEs have been conducted within the VA-AHT model to evaluate the proposed connection between DAD sampling and Δ*E*_a_’s.^14,25^ In the meantime, other frameworks were also used to replicate the results in an attempt to examine where there is such a “DAD–Δ*E*_a_” relationship. Those include nonadiabatic vibronic models of H-tunneling (another form of the VA-AHT model),^26–28^ the ensemble-averaged variational TS theory with multidimensional H-tunneling,^29–31^ and the empirical valence bond theory.^32–34^ Nonadiabatic H-tunneling models have successfully reproduced the T-dependence of large KIEs for proton-coupled electron transfer reactions catalyzed by soybean lipoxygenases, supporting the thermally activated DAD sampling concept.^8,15,21,22,26,35,36^ While the VA-AHT models have been well applied to interpret the DAD–KIE correlations in adiabatic hydride-transfer reactions of some oxidoreductases,^14,15,21,37^ it has also been proposed that such adiabatic tunneling may proceed from the ground state of the reactant to an excited state of the product within the TRS, complicating the DAD–KIE relationship.^36,38^ Moreover, simulations using alternative theoretical approaches have sometimes attributed weak T-dependence of hydride KIEs to the relative insensitivity of DADs to temperature.^11,15,26,30,33,39^ In contrast, other studies suggest that weak T-dependence of hydride KIEs may arise from temperature-induced changes in the microscopic reaction mechanism, such as shifts in the position of the TS or alterations in the shape of the potential energy barrier.^29,33^ Thus, whether T-dependent KIEs directly reflect DAD sampling remains an open and unresolved question.

Our group has investigated the DAD–Δ*E*_a_ correlation by studying hydride-transfer reactions in solution.^40–51^ In contrast to enzymatic systems, solution reactions allow systematic variation of both molecular structure and solvent environment, enabling rigorous evaluation of how structural rigidity or dynamics influences the T-dependence of KIEs. Our central hypothesis is that a looser H-tunneling system with a broadly distributed longer DADs exhibits a larger T-dependence of KIEs, reflected in a larger Δ*E*_a_ value. We have investigated how variations in electronic structures, remote heavy-group vibrations, and solvent environments influence donor–acceptor geometries and Δ*E*_a_ values in hydride tunneling reactions of the NADH/NAD^+^ models. Overall results appear to support our rigidity (DAD)–Δ*E*_a_ relationship hypothesis. More recently, we found that only a relatively small fraction of the reaction free-energy (Δ*G*°) contributes to modulation of the Δ*E*_a_ values.^51^ This observation led us to formulate a VA-AHT-inspired model in which a distinct free-energy term, Δ*G*°_DAD_, is proposed to govern the activation required for DAD sampling. Within this framework, a Δ*G*° closer to thermoneutrality is associated with a looser TRS structure, broader DAD sampling, and consequently a larger Δ*E*_a_ value. These findings provide further support for the proposed relationship between DAD flexibility and the T-dependence of KIEs.

In this work, we designed hydride-transfer systems in which the nucleus donor–acceptor separation at the TRS is progressively augmented to further test our hypothesis. According to our hypothesis, increasing this separation will lead to a larger Δ*E*_a_ value, mimicking the enzyme versus mutant dynamics on the tunneling DAD sampling process. One approach is to introduce steric effects to physically separate the donor and acceptor; and another is to employ reactions that proceed through distinctly different TRS structures, thereby modulating DADs. Hydride-transfer reactions between two carbons of the NADH/NAD^+^ models that contain reactive 1,4-dihydropyridine/pyridinium rings typically proceed via a direct one-step mechanism.^52–54^ However, when particular hydride acceptors with high one-electron reduction potentials are employed, reactions could follow a sequential electron–proton–electron (e-H^+^-e) transfer pathway.^52,54–60^ In one-step mechanisms, hydride-transfer occurs within tightly bound charge-transfer (CT) complexes, whereas in multistep mechanisms, proton-transfer occurs between radical ions that can be more widely separated by solvation effects. According to the DAD sampling framework for nucleus transfer reactions, the multistep pathway, associated with longer DADs for proton-transfer, would exhibit larger Δ*E*_a_ values. Furthermore, in both mechanisms, systematic modification of donor/acceptor structures allows further tuning of DADs, providing a means to examine their relationship with Δ*E*_a_’s in the context of our hypothesis. Note that we have already successfully applied the strategy to one-step hydride-transfer reactions,^40,41,45–47,49,51^ and we will do the same for the multistep reactions in this work.

Herein, we will use various hydride-transfer reactions of NADH/NAD^+^ models to investigate the assumed effects of steric and reaction-type variations on the Δ*E*_a_’s. The 10-methyl-9-substituted­(R) acridines (RMAH) and their oxidized forms (RMA^+^BF_4_^–^) are used as hydride donors/acceptors, in which the size of the 9-α-R group increases from R=H (for MAH/MA^+^) to CH_3_ (MeMAH/MeMA^+^) to Ph (PhMAH/PhMA^+^). Reactions include the apparent hydride-transfer reactions from the Hantzsch ester (HEH) to RMA^+^ (eq 1), from RMAH to 2,3-dichloro-5,6-dicyanobenzoquinone (DDQ) (eq 2) and to a distinct iron­(IV)­oxo complex ([(BnTBEN)­Fe­(IV)=O]­(OTf)_2_) (eq 3). The BnTPEN ligand denotes *N*-benzyl-*N*,*N*′,*N*′-tris­(2-pyridylmethyl)­ethane-1,2-diamine. Note that reactions (2) and (3) use a multistep e-H^+^-e transferreaction mechanism.^58,60^


1

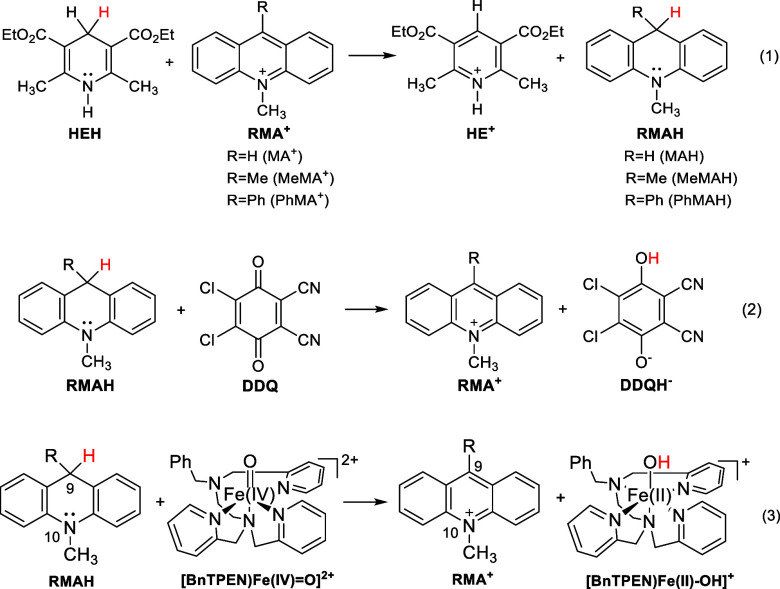




The KIEs and Δ*E*_a_’s were determined for the three series of apparent hydride-transfer reactions in acetonitrile. We found that the Δ*E*_a_ is augmented by (i) steric effects that separate the donor and acceptor, (ii) solvent-induced separation of the radical ion-pair donor and acceptor, and (iii) like-charge repulsion between the donor and acceptor. Overall, these results support our central hypothesis regarding the relationship between system rigidity and Δ*E*_a_ in nucleus transfer reactions. Notably, the reactions (3) show unusually high KIEs and Δ*E*_a_’s. Our analysis suggests that this is due to the relatively flexible proton-transfer systems and extended DADs. Whether the observed KIE and Δ*E*_a_ genuinely reflect the actual proton-transfer step of the multistep reactions (2) and (3) is also discussed.

## Experimental

### General Procedures

Syntheses of hydride donors of MA^+^, MeMA^+^, PhMA^+^, and their reduced forms MAH, MeMAH, PhMAH, as well as HEH, and their dideuterio analogues, have been reported by us.^45,47,61,62^ The commercially available DDQ was recrystallized from chloroform before use. The BnTPEN ligand and [Fe­(II)­BnTPEN]­(OTf)_2_ were synthesized using a literature procedure.^63,64^ The desired [(BnTPEN)­Fe­(IV)=O]­(OTf)_2_ product was synthesized by oxidation of [Fe­(II)­BnTPEN]­(OTf)_2_ with iodosylbenzene (molar ratio 1:1.1) in acetonitrile in an argon atmosphere.^65^ The iodosylbenzene was prepared from the reaction of phenyliodine­(III) diacetate in a NaOH aqueous solution, following the same synthesis procedure from a literature.^66^ The iron­(IV)­oxo compound was not isolated but prepared immediately before use for kinetics experiments in acetonitrile, which requires using a known and calculated molarity of the iron­(II) complex to successfully achieve pseudo-first order kinetics conditions for its reactions with RMAH (see Kinetic Procedures).^65^ Its structure was confirmed with checking its characteristic UV–vis absorption at λ_max_ = 739 nm that we also used to monitor for kinetic measurements.

HPLC-grade acetonitrile was redistilled twice under nitrogen, with the presence of KMnO_4_/K_2_CO_3_ (to remove the reducing impurity) and P_2_O_5_ (to remove water) in order, for kinetic measurements.

### Kinetic Procedures

Kinetic measurements were carried out by following the same procedures in our publications. Freshly distilled acetonitrile under nitrogen was used for each day’s measurements. The pseudo-first order rate constants (*k* ^pfo^’s) were determined using the SF-61DX2 Hi-Tech KinetAsyst double-mixing stopped-flow UV–vis instrument. The Absorbance *(Abs)*–time data of more than 12.5 half-lives of the reaction were collected. Using the software integrated with the instrument, 12 half-lives of the *Abs*–time data (for ∼99.98% completion of the reaction) was fitted to derive a *k* ^pfo^ value. The second-order rate constant (*k*_2_) was calculated as *k* ^pfo^/[excess substrate]. The temperature and concentration conditions as well as the wavelength that we followed for kinetic measurements are listed in Tables S1–S6. We use the same [excess substrate] for both isotope transfer reactions and same solutions for measurements at all temperatures to determine a Δ*E*_a_ value (KIE = *k*_2H_/*k*_2D_ = *k* ^pfo^_H_/*k* ^pfo^_D_).

In standard procedures, solutions were drawn up in 5 mL syringes and a few drops of solution were used to rinse the stopped-flow syringe and discarded, after which 1 mL of the kinetic sample was loaded. For reactions containing Fe­(IV)=O complex solutions, these were drawn up using a needle-tipped Luer lock syringe. The needle was then removed, large gas bubbles were eliminated, and a 0.22 μm nylon syringe filter was attached to remove excess solid PhIO particulates before filling the stopped-flow syringe.^65^

Measurement of *k* ^pfo^ was performed over a temperature range of 40 °C at the same day and repeated on two other days. Three or six consecutive kinetic runs (again, each covering at least 12.5 half-lives) were conducted back-to-back for each isotopic reaction. The procedure was then repeated at other temperatures as quickly as possible (e.g., 15, 25, 35, 45, 55 °C, in order) to maintain the constant instrument settings and minimize possible aging of the reaction solutions. (Although the reaction solutions are relatively stable, they were wrapped with aluminum foil and kept in a refrigerator between runs to avoid introducing any unknown impurities. The Fe­(IV)=O complex solution was stored under an argon atmosphere.) Repetitions of kinetic measurements at different days sometimes used different batches of substrates and sometimes were done by different workers. That was to eliminate the effect of different impurity from unknown sources or human errors on the KIE measurements. One KIE value was obtained from *at least* 9 repetitions (3 days of measurements with 6 (or 3, in some cases) repetitions each day).

Arrhenius correlation of *k*_2_’s was used to derive *E*_a_’s (see the exemplified Arrhenius plots in Figure S4). The correlation coefficient *R* ^2^ values range from 0.9990 to 1.0000, mostly very close to or exactly 1.0000. Standard deviation for each *E*_a_ was calculated using the Excel LINEST formula and that for Δ*E*_a_ was derived using the standard deviations for *E*_aH_ and *E*_aD_, respectively. Pooled standard deviations were reported, and the standard deviations for the average values of different days of measurements are also provided in Tables S1–S6 for comparison.

## Results

### Direct Hydride Reduction of RMA^+^ by HEH (1): Medium T-Dependence of Small KIEs

We have reported the T-dependence of KIEs for the reactions of MA^+^ and PhMA^+^ with HEH in acetonitrile.^40,45^ The Arrhenius plots of KIEs of all three reactions are plotted in Figure 1. These kinetic data including KIEs and Δ*E*_a_’s are listed in Table 1. The size of the KIEs (4.45–5.31) and Δ*E*_a_’s (0.94–1.19 kcal/mol) fall within their typical range for the one-step hydride-transfer reactions that we have reported. The KIEs in such reactions are usually below 6 and the Δ*E*_a_’s range from near 0 to ∼1.4 kcal/mol.^40–47,49–51^

**Figure 1:**
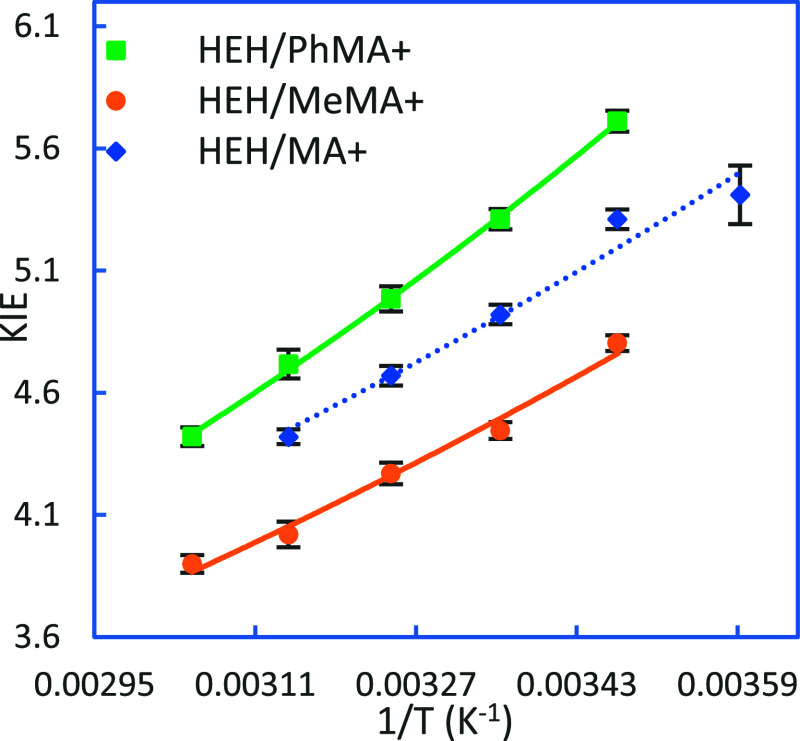
Arrhenius plots of KIEs for the reactions of HEH with 9-substituted-10-methylacridinium ions (RMA^+^) (from 15.0 to 55.0 °C for the reactions of PhXn^+^ and MeMA^+^, 5.2 to 45.0 °C for the reaction of MA^+^) in acetonitrile. Lines represent nonlinear regression using an Arrhenius-type exponential equation (KIE vs EXP­(1/T)). Plot of HEH/MA^+^ is reproduced from ref 40 (Copyright [2019] American Chemical Society.).

**Table 1:** Δ*G*°(H^–^_T_)’s, KIEs and Δ*E*_a_’s for the Multistep Hydride-Transfer Reactions of [(BnTPEN)­Fe­(IV)=O]^2+^ and DDQ in Acetonitrile^a^

reactions	*k*_2H_^25 ^°^C^ (M^–1^ s^–1^)	KIE^25 ^°^C^	Δ*E*_a_ (kcal/mol)
*HEH*			
-MA^+^ ^b^	1.56(0.01) × 10^2^	4.92 (0.04)	0.94 (0.12)
-MeMA^+^	2.11(0.01) × 10	4.45 (0.03)	0.98 (0.09)
-PhMA^+^ ^c^	1.37(0.08) × 10	5.31 (0.04)	1.19 (0.07)
*DDQ*			
-MAH	too fast		
-MeMAH	1.54(0.01) × 10^5^	4.33 (0.05)	1.14 (0.05)
-PhMAH	1.24(0.01) × 10^5^	5.45 (0.07)	1.62 (0.13)
*ClQ*_*4*_			
-MAH^d^	1.92(0.01) × 10	8.53 (0.16)	1.70 (0.13)
*[(BnTPEN)Fe(IV)*=*O]* ^*2+*^			
-MAH	2.76(0.03) × 10^3^	16.4 (0.3)	3.04 (0.23)
-MeMAH	2.35(0.05) × 10^2^	11.4 (0.3)	3.71 (0.14)
-PhMAH	1.74(0.01) × 10^2^	12.3 (0.4)^e^	5.38 (0.35)

a Numbers in parentheses are pooled standard deviations (standard deviations calculated from the mean values of three independent experiments are included in Supporting Information, which are comparable to the pooled standard deviations).

b From ref 40.

c From ref 45.

d From ref 48.

e KIE = 17.5 was calculated using the data from the literature.^58^.

### Multistep Hydride Reduction of Benzoquinones (DDQ and Chloranil) by RMAH: Large T-Dependence of Relatively Large KIEs

The mechanism of DDQ reduction by RMAHs in acetonitrile has also been studied in the literature.^58^ The reaction was proposed to proceed via a multistep e-H^+^-e transfer mechanism (Scheme 1). Supporting evidence included UV–vis and ESR spectroscopic observation of the RMAH^+•^ species in acid-promoted reaction with a benzoquinone structure (and a Fe­(IV)=O complex) in acetonitrile.^58,59^ This finding suggests that the corresponding radical ion-pair complex intermediates can be separated by the solvation effects. To our knowledge, the Δ*E*_a_’s of these reactions have not been previously reported. While the kinetics for the reaction of DDQ with MAH is too fast to be accurately determined at a wide range of temperature conditions, we determined the KIEs (from 5 to 45 °C) and Δ*E*_a_’s for the reactions of MeMAH and PhMAH in acetonitrile. The Arrhenius plots of KIEs for both reactions are shown in Figure 2. The rate constants and KIEs at 25 °C as well as Δ*E*_a_’s are listed in Table 1. These KIE values are relatively small (4.33 and 5.45, respectively); however, the Δ*E*_a_ for the reaction of PhMAH (1.62 kcal/mol) is larger than those typically observed for one-step hydride-transfer reactions between two carbon centers.

**Scheme 1:**
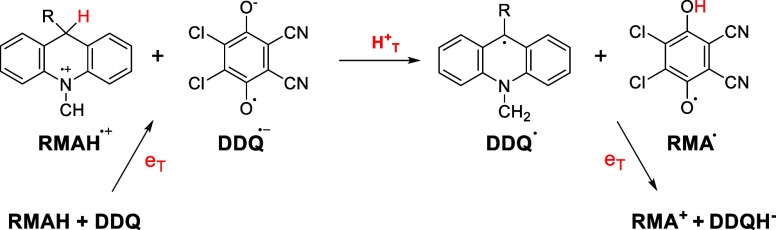
Multistep Mechanism of the Reactions of DDQ (2)

**Figure 2:**
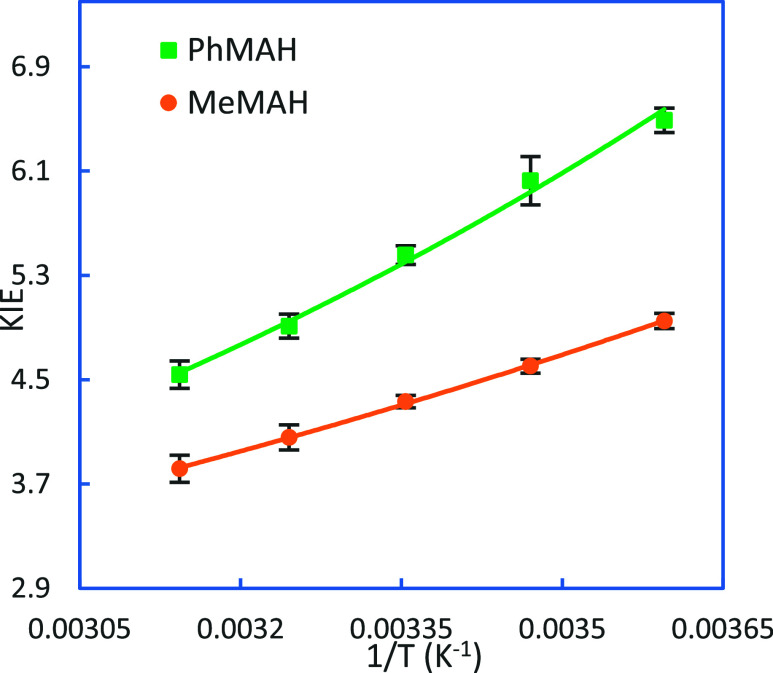
Arrhenius plots of KIEs for the reactions of 9-substituted-10-methylacridines (R)­MAH with DDQ (from 5.0 to 45.0 °C) in acetonitrile. Lines represent nonlinear regression using an Arrhenius-type exponential equation (KIE vs EXP­(1/T)).

We have also reported measurements for the multistep reactions of MAH and its 10-substituted acridine derivatives with another benzoquinone, 2,3,5,6-tetrachlorobenzoquinone (chloranil, Cl_4_Q), in the same solvent.^48^ These reactions generally show larger KIEs and greater Δ*E*_a_’s over the experimental temperature range of 15–55 °C.

For example, the two values for the reaction of MAH are 8.90 and 1.67 kcal/mol, respectively. These data are also listed in Table 1 for direct comparison with the corresponding reactions involving DDQ. We also attempted to study the reactions of MeMAH and PhMAH with chloranil, but those reactions are too slow, and the resulting kinetic results are insufficiently stable to produce reliable KIE results.

### Multistep Hydride Reduction of [(BnTPEN)­Fe­(IV)=O] by RMAH: Unusually Large T-Dependence of Large KIEs

Functionality of the inert C–H bonds by high valent iron-oxo structures in enzymes is an important biological process. The reactions are widely believed to be facilitated by H-tunneling. The primary evidence comes from the observation of large nonclassical KIEs. Representative examples include cytochromes P450 (H/D KIE ∼ 15),^67^ soluble methane monooxygenase (∼50),^68^ taurine dioxygenase (∼50),^69^ and soybean lipoxygenase (SLO, ∼80),^17^ as well as a double mutant of SLO (∼700).^12^ There is no consensus as to why these reactions show high KIEs and thus a pronounced degree of H-tunneling.^12,70^ The Δ*E*_a_’s have been determined for SLOs and their mutants that were strategically designed to increase DADs. These studies show that the Δ*E*_a_ increases with DAD.^12,17,71^ Simulations with the nonadiabatic VA-AHT model support the proposed DAD−Δ*E*_a_ relationship.^12^

Kinetics of hydride reduction of the [(L)­Fe­(IV)=O]^2+^ (L = ligand, including BnTPEN) complexes by NADH models has also been reported.^58^ Evidence shows that this class of reactions take place by a multistep e-H^+^-e transfer mechanism, supported by the observation of an ESR signal corresponding to RMAH^+•^ in acetonitrile. Scheme 2 illustrates the general three-step mechanism for the reductions by RMAH in which a proton is transferred from the radical cation acid (RMAH^+•^) to the one-electron reduced intermediate, [(L)­Fe­(III)=O]^+^.

**Scheme 2:**
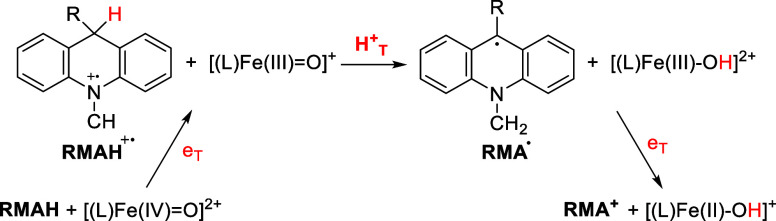
Multistep Mechanism of the Reactions (3) (L = BnTPEN); Proton-Transfer Takes Place within Two Positively Charged Species

This is a proton-transfer step from RMAH^+•^ to the basic O of the positively charged [(L)­Fe­(III)=O]^+^ intermediate. The KIEs of the reductions of [(BnTPEN)­Fe­(IV)=O]^2+^ by MAH and PhMAH in acetonitrile at 25 °C were reported to be 17.6 and 17.5, respectively, strongly suggesting a proton tunneling process in these reactions.^58^ The T-dependence of KIEs of these reactions has not yet been reported in the literature, but that for the reaction of another metal complex, [(TMC)­(Cl)­Cr­(III)­(O_2_)]^+^, with MAH in acetonitrile has been determined at low temperatures. In the latter multistep hydride-transfer reaction, the proton KIE was reported to be 477 at 233 K and the Δ*E*_a_ was found to be 10.8 kcal/mol measured at temperatures ranging from 233 to 253 K.^72^

We determined the kinetics of the reactions of [(BnTBEN)­Fe­(IV)=O]^2+^ by RMAH in acetonitrile at temperatures ranging from 5 to 45 °C. The Arrhenius plots of KIEs are presented in Figure 3. Reaction rates and KIEs at 25 °C as well as the Δ*E*_a_’s are also listed in Table 1. The KIEs at 25 °C are 16.4 (for MAH), 11.4 (MeMAH), and 12.3 (PhMAH), respectively. The Δ*E*_a_’s are 3.04 (for MAH), 3.71 (MeMAH), and 5.38 kcal/mol (PhMAH). These KIEs and Δ*E*_a_’s are unusually large and represent the highest reported to date for apparent hydride-transfer reactions of NADH/NAD^+^ model systems.^40–50^

**Figure 3:**
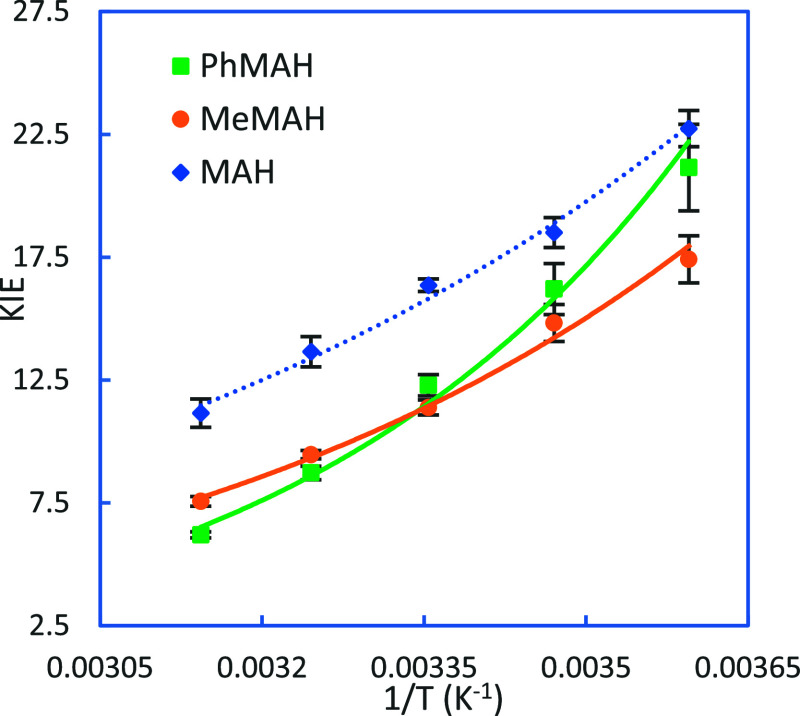
Arrhenius plots of KIEs for the reactions of 9-substituted-10-methylacridines (R)­MAH with [(BnTPEN)­Fe­(IV)=O]­(OTf)_2_ (from 5.0 to 45.0 °C) in acetonitrile. Lines represent nonlinear regression using an Arrhenius-type exponential equation (KIE vs EXP­(1/T)).

## Discussion

### Do the Observed KIE and Δ*E*_a_ Fully Represent the Proton-Transfer Step in the Multistep Reactions (2) and (3)?

Previous results have shown that the second electron-transfer step of the e-H^+^-e transfer mechanism of the reactions (2) and (3) is rapid.^57,58^ Accordingly, *k*_2_ = *k*_et_ × *k*_H^+^_/(*k*_–et_ + *k*_H^+^_), where *k*_et_ and *k*_–et_ are the rate constants for the forward and backward processes of the first electron-transfer step, and *k*_H^+^_ is for the subsequent proton-transfer. If *k*_–et_ ≫ *k*_H^+^_, *k*_2_ ≈ *K*_et_ × *k*_H^+^_, where *K*_et_ is the pre-equilibrium constant for the first electron-transfer step. Under these circumstances, the observed KIE primarily represents the proton-transfer step. In contrast, when *k*_–et_ and *k*_H^+^_ are comparable, the observed KIE is suppressed relative to that for the proton-transfer process. Therefore, the competition between *k*_–et_ and *k*_H^+^_ in reactions (2) and (3) could mask the real KIE and thus the Δ*E*_a_ for the actual proton-transfer step of our interest, i.e., both parameters could be potentially suppressed.

As shown in Table 1, the observed KIEs at 25 °C range from 4.33 to 5.43 for the reactions of DDQ, 8.53 for the reaction of ClQ_4_, and 11.4–16.4 for the reactions of [(BnTBEN)­Fe­(IV)=O]^2+^. These relatively large KIEs suggest that proton transfer constitutes a major contributor to the overall rate limitation. Furthermore, based on the one-electron reduction potentials of the DDQ (0.51 V vs SCE in acetonitrile^56^), Cl_4_Q (0.01 V^56^), and [(BnTBEN)­Fe­(IV)=O]^2+^ (0.49 V^73^), together with the oxidation potentials of the MAH (0.81 V), MeMAH (0.84 V), and PhMAH (0.88 V),^57^ the initial electron-transfer steps are highly endergonic (i.e., *K*_et_ ≪ 1). Consequently, the *k*_–et_ is expected to compete effectively with the *k*_H^+^_, making the rapid pre-equilibrium approximation *k*_–et_ ≫ *k*_p_ plausible, consistent with the literature analysis.^57^

For the reactions of [(BnTBEN)­Fe­(IV)=O]^2+^, the exceptionally large KIEs suggest that the proton-transfer dominates the rate-limiting step, implying a particularly small *k*_H^+^_ relative to *k*_–et_. Therefore, the condition *k*_–et_ ≫ *k*_H^+^_ is well satisfied, and the observed largest KIE and Δ*E*_a_ should closely represent the proton-transfer step. This conclusion is also the same as that from the literature.^58^

The DDQ reactions appear to represent a slightly different situation. Although the DDQ and [(BnTPEN)­Fe­(IV)=O]^2+^ possess similar reduction potentials, the DDQ reactions are approximately 700-fold faster (Table 1). This observation would suggest larger *k*_H^+^_ values, making competition between *k*_H^+^_ and *k*_–et_ likely more significant and increasing the likelihood of the KIE suppression. This interpretation appears consistent with the relatively small observed KIEs (4.33 and 5.43), which are lower than those commonly observed for proton-transfer reactions. It may also explain why the measured Δ*E*_a_ values for the DDQ reactions are smaller than that of the analogous Cl_4_Q reaction, whose substantially larger KIE (8.53) suggests less suppression of the actual proton-transfer KIE.

Taken together, the observed KIEs and Δ*E*_a_’s predominantly represent the proton-transfer step for reactions (3) and the reaction of Cl_4_Q, whereas they may do so for a lesser extent for the DDQ reactions. Nevertheless, the observed DDQ values represent conservative lower-boundestimates of the true values. This conclusion establishes an important trend that the proton-transfer Δ*E*_a_ values in reactions (2) may be comparable to that of the Cl_4_Q reaction and both remain intermediate between those observed for the hydride-transfer reactions in (1) and the proton-transfer steps in (3). Therefore, in the following discussion of the DAD−Δ*E*_a_ relationship, we use the relative ordering of the observed Δ*E*_a_ values.

### Comparison of the Δ*E*_a_’s among Three Series of Reactions

We have previously reported the KIEs and Δ*E*_a_’s for a series one-step hydride-transfer reactions of NADH/NAD^+^ models in acetonitrile.^40–47,49–51^ In these reactions, the KIEs are generally smaller than 6 and Δ*E*_a_ ranges from near 0 to ∼1.4 kcal/mol. As described in the Introduction, we have identified a trend in which more exergonic reactions exhibit smaller Δ*E*_a_ values.^41,45,46,51^ This is consistent with our central hypothesis regarding the DAD–Δ*E*_a_ relationship as a more exergonic reaction could correspond with a tighter CT complex structure at both the reactant and TRS states. Moreover, we have also reported the same for the multistep reactions of MAH and its 10-alkylated acridine derivatives with chloranil (ClQ_4_) in the same solvent.^48^ These reactions typically show larger KIEs and larger Δ*E*_a_’s (Table 1 lists one example with MAH). Such behavior has been interpreted in terms of differences in system tightness, based on the nucleus/proton DAD sampling mechanism. In the multistep mechanism, proton-transfer takes place between two radical ions (MAH^+•^/Cl_4_Q^–•^) which are readily separated due to solvation effects in the polar solvent acetonitrile.

Here in this work, the Δ*E*_a_’s are also larger in the multistep reaction mechanisms. They are 1.14–1.62 kcal/mol for the reactions of RMAH (R = Me and Ph) with DDQ (Reactions 2) and 3.04–5.38 kcal/mol for the reactions of all three RMAH with [(BnTBEN)­Fe­(IV)=O]^2+^ (Reactions 3), as compared to 0.94–1.19 kcal/mol for the one-step hydride-transfer reactions of HEH with RMA^+^ (Reactions 1) (Table 1) and near 0 to ∼1.4 kcal/mol for other one-step reactions of NADH/NAD^+^ models that we have reported.^40–47,49–51^ In the one-step reactions including reactions (1), the covalent CT bonding leads to tight TRSs (Figure 4A). In contrast, when the reactive complex becomes a radical ion-pair, as in reactions (2) and the reactions of Cl_4_Q,^48^ the two radical ions (in RMAH^+•^/DDQ^–•^ and in MAH^+•^/Cl_4_Q^–•^) tend to separate due to solvation, although they may remain loosely associated through electrostatic interactions. This results in a looser TRS compared to CT-complex systems (Figure 4B). In the multistep reactions of [(BnTBEN)­Fe­(IV)=O]^2+^ (3), proton transfer occurs from RMAH^+•^ to the basic O center of [(BnTBEN)­Fe­(III)=O]^+^. Because both species are positively charged, electrostatic repulsion further separates them, leading to proton transfer within a more weakly associated acid–base complex characterized by longer DADs. This effect may be amplified by steric hindrance from the bulky BnTBEN ligand (Figure 4C). Overall, the system rigidity decreases while Δ*E*_a_ increases in progressing from reactions (1–3). This trend is consistent with our hypothesis that looser nucleus-transfer systems give rise to larger Δ*E*_a_ values.

**Figure 4:**
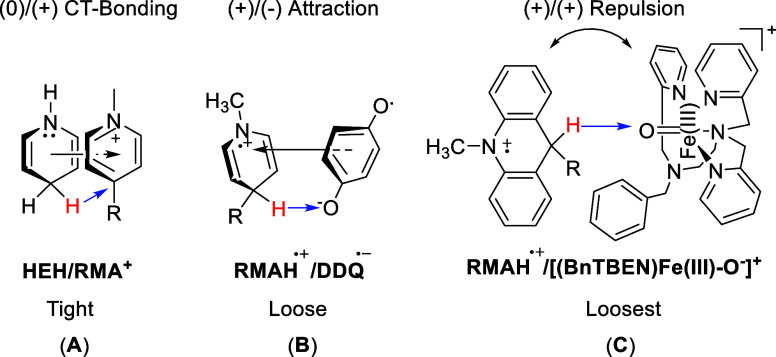
Schematic description of the nucleus donor–acceptor progressive separations from (A–C) (In (A,B), only the reactive rings are drawn). (A) Direct hydride-transfer between NADH/NAD^+^ models, including from neutral HEH to positively charged RMA^+^ in this work, in a tightly covalently bonded π–π CT complex; (B) proton-transfer from positively charged radical cation RMAH^+•^ to negatively charged radical anion of the benzoquinone structures (DDQ^–•^ and Cl_4_Q^–•^) in a solvation-induced separation of radical ion-pair; (C) proton-transfer from positively charged radical cation RMAH^+•^ to the overall positively charged [(BnTPEN)­Fe­(III)=O]^+^. In (C), the steric interaction between R and the bulky BnTPEN ligand is expected to be severe.

### Steric Effects on the Δ*E*_a_’s in Each Series of the Reactions

In all three series of reactions, the reaction rate (*k*_2_) decreases as the bulkiness of the R group increases from MAH to MeMAH to PhMAH (Table 1). For the radical cation intermediates in the reactions (2) and (3), the unimolecular deprotonation rates (*k*_d_) of MAH^+•^, MeMAH^+•^, and PhMAH^+•^ radical cations in acetonitrile have been reported as 6.4, 1.1, and 4.1 s^–1^, respectively.^74^ Notably, the slower bimolecular reaction rates (*k*_2_) observed for PhMAH compared to MeMAH in these multistep processes are opposite to the trend in their corresponding *k*_d_ values. Because the one-electron oxidation potentials of the three RMAHs are comparable so that the equilibrium constants (*K*_eq_) of the first electron-transfer steps of the three reactions are similar,^58^ the *k*_2_ order could reflect the rate order for the actual proton-transfer processes. While the *k*_d_ reflects little steric effect, the discrepancy suggests that the steric effect plays an important role in determining the rates of the proton-transfer processes where the KIE and Δ*E*_a_ values arise.

The Δ*E*_a_ was observed to significantly increase as R changes from H to/or Me to Ph in the reactions (2) and (3) (Table 1). In the reactions (2), the Δ*E*_a_’s are 1.14 and 1.62 kcal/mol for the reactions of MeMAH and PhMAH, respectively. In the reactions (3), they are 3.04 (MAH), 3.71­(MeMAH), and 5.38 kcal/mol (PhMAH). In both series of the reactions, the Δ*E*_a_ is increased by ∼30% from the reactions of MeMAH to PhMAH. Since the R group does not significantly affect the p*K*_a_ of the RMAH^+•^, (for example, the enthalpy changes of the acidic dissociation of MAH^+•^ and PhMAH^+•^ in acetonitrile are 9.2 and 9.0 kcal/mol, respectively^75,76^ ), the observed increase in Δ*E*_a_’s can be explained in terms of the steric augmentation of the donor–acceptor separation. Therefore, these results also suggest that a looser system with broader DAD distributions corresponds with a larger Δ*E*_a_ value.

It should be noted that, of all the reactions from this work, the reaction between PhMAH and [(BnTPEN)­Fe­(IV)=O]^2+^ exhibits the largest Δ*E*_a_ value (5.38 kcal/mol). In this TRS, both the (+)/(+) charge repulsion and the spatial repulsion between the large Ph group from PhMAH and the aromatic groups of the BnTPEN (Figure 4C) would render the loosest TRS and longest DAD.

As compared to the multistep reactions, the Δ*E*_a_ of the one-step hydride-transfer reactions (1) increases only slightly from the reactions of MA^+^ (0.94 kcal/mol) to MeMA^+^ (0.98) to PhMA^+^ (1.19). As mentioned earlier, we have found that in a series of exergonic one-step hydride-transfer reactions between two carbons, Δ*E*_a_ increases as the free energy (Δ*G*°) becomes less negative.^41,45,46,51^ Herein, the literature values of hydride affinities (-Δ*G*°_H_-) of the MA^+^ and PhMA^+^ in acetonitrile are 76.2 and 74.1 kcal/mol, respectively, that of the oxidized form of HE^+^ is 64.4 kcal/mol.^75^ The Δ*G*° of the two reactions are calculated to be −11.8 and −9.7 kcal/mol, respectively. Comparison of the Δ*G*° values suggest that the Δ*E*_*a*_ would be larger for the reaction of PhMA^+^ than MA^+^. This analysis primarily reflects electronic effects. Since steric effects are also expected to increase Δ*E*_a_ in the same direction, the observed increase from MA^+^ to PhMA^+^ is likely only partially attributable to steric augmentation of the donor–acceptor separations.

## Conclusions

The T-dependence of H/D KIEs was determined for the three series of apparent hydride-transfer reactions of NADH/NAD^+^ models in acetonitrile to test our hypothesis that nucleus donor–acceptor separation augments the T-dependence of KIEs (i.e., increases the Δ*E*_a_ values). Donor–acceptor separation was modulated through steric effect augmentation, solvation-induced separation of the radical ion-pair, and the like-charge repulsion effect. Across the three series of reactions, Δ*E*_a_ increases from one-step hydride-transfer reactions (1), which involves a tight CT complex between donor and acceptor, to e-H^+^-e multistep reactions (2), where a proton transfers within a radical ion-pair that can be separated by solvation effect, and finally to multistep reactions (3), which involve like-charged donors and acceptors in proton-transfer step. In the reactions (3), steric hindrance from the bulky BnTBEN ligand could further increase donor–acceptor separation. These observations are consistent with and support our hypothesis.

Thus far, we have investigated the effects of electronics, sterics, solvents, remote heavy group vibrations, as well as donor–acceptor electronic repulsions on the T-dependence of KIEs for the apparent hydride-transfer reactions of NADH/NAD^+^ model compounds. We found that a system of looser interaction between donor and acceptor generally exhibits a larger Δ*E*_a_ value, supporting our central hypothesis regarding the DAD−Δ*E*_a_ relationship. Therefore, the Δ*E*_a_ magnitude of these hydride-transfer reactions, irrespective of one-step and multistep mechanisms, could be an indicator of the *nucleus* tunneling system rigidity or the width of the DAD distributions. These results provide valuable insights for evaluating current H-tunneling models and guiding the development of future theoretical frameworks. For example, a quadratic relationship between Δ*E*_a_ and DAD distribution width for nonadiabatic proton coupled electron transfer reactions predicts that Δ*E*_a_ reaches a maximum and then decreases as the distribution continues to broaden, in contrast to the consistently increasing behavior predicted by the linear approximation treatment.^77^ Whether an analogous mathematic relationship between Δ*E*_a_ and DAD distribution width holds for the more adiabatic hydride and proton transfer reactions remains unknown.

Overall, our results suggest that the T-dependence of KIEs can be used to evaluate the DAD sampling mechanism associated with the protein dynamics in the active site of the enzymes. The observed shift from T-independent KIEs in wild-type enzymes to T-dependent KIEs in mutants could be attributed to increased donor–acceptor separation resulting from weakened protein-mediated compression of DADs in the mutants.

As an additional observation, we found that KIE increases from reaction systems (2) (including the reactions of chloranil) to (3), both of which involve proton transfer processes. Although there is no consensus as to why H-transfer reactions involving biological high-valent iron oxidants and other metal complexes show unusually high KIEs,^60,70^ our results suggest that such high KIEs correlate with relatively loose proton tunneling processes.

## Supplementary Material



## Data Availability

The data underlying this study are available in the published article and its online Supporting Information.

## Notes

Synthesis procedures, rate constants, KIEs, kinetic measurement conditions, and original kinetic data (PDF)
