# Supplementary material for: Donor–Acceptor Separation Augments Temperature Dependence of Kinetic Isotope Effects in NADH Model Hydride Transfer Reactions: Mimicking Enzyme versus Mutant Dynamics
Source: J Phys Chem B. 2026 Jul 7;130(28):7206–14. doi: 10.1021/acs.jpcb.6c02719 (PMC13383721; doi:10.1021/acs.jpcb.6c02719)
Supplement: Supplementary file 1 [file jp6c02719_si_001.pdf]

Supporting Information

Donor–Acceptor Separation Augments Temperature Dependence of Kinetic Isotope Effects in NADH Model Hydride Transfer Reactions: Mimicking Enzyme versus Mutant Dynamics

Nicholas DeGroot, Pratichhya Adhikari, Bibesh Pokhrel, Grishma Singh, Yun Lu*
Department of Chemistry, Southern Illinois University Edwardsville, Edwardsville, Illinois 62026, United States

yulu@siue.edu

Synthesis procedures …… S2
Rate constants and KIEs (Tables S1-S6)…… S3
Kinetic CCD scans of the reaction of [(BnTPEN)Fe(IV)=O]$^{2+}$ with RMAH…… S4
Reference…… S5
Plots of temperature dependence of rates…… S6
Data availability statement…… S7

Synthesis of N-benzyl-N,N'N''-tris(2-pyridylmethyl)-1,2-diaminoethane (BnTPEN)[1]

1.50 grams (10 mmol) of N-benzylethylenediamine in 15 mL of dichloromethane and 5.20 grams (32 mmol) of 2-chloromethylpyridine hydrochloride in 15 mL of water were mixed. This was followed by adding 15 mL 6.4 M aqueous NaOH solution. The reaction solution was stirred vigorously over a week at room temperature. During the reaction, 0.5 grams batches of potassium carbonate were added to the reaction solution every two to three days.

The reaction mixture was extracted with dichloromethane. The BnTPEN product was separated by column chromatography (eluted using a 5% v/v MeOH/Dichloromethane). The resulting solid product was recrystallized using hexanes three times. The yield was 50%. Melting point: 85.0 – 86.0 °C. ^{1}H NMR δ (ppm, CD_3CN): 8.43-8.41 (3H, m), 7.64-7.60 (3H, td), 7.43-7.41 (3H, d), 7.30-7.13 (8H, m), 3.68 (4H, s), 3.63 (2H, s), 3.55 (2H, s), 2.66-2.61 (4H, m)

Synthesis of (N-benzyl-N,N',N''-tris(2-pyridylmethyl)-1,2-diaminoethane-κ^{5}N)iron(II) bis(trifluoromethanesulfonato) ([(BnTPEN)Fe(II)](OTf)$_2$)[1]

2.63 grams (6.22 mmol, 1.1x excess) of BnTPEN ligand and 2.00 grams (5.65 mmol) of Fe(II)(OTf)$_2$ were added to 10mL of acetonitrile. The mixture was stirred overnight. The desired solid product was obtained by concentrating the solution to 2-3 mL, and transferred to 50 mL of diethyl ether. The latter mixture was stirred on an ice bath and the solid precipitate product was filtered. Final product yield was 87%.

Synthesis of Iodosylbenzene (PhIO)[1]

1.53 grams (4.75 mmol) of phenyliodine(III) diacetate solid was added to 25 mL of 6M aqueous NaOH solution, and stirred overnight. A yellowish-white solid product formed overtime. After the reaction was complete, suspended solid product was filtered from the solution and rinsed with chloroform to remove any iodobenzene byproduct. Final product yield was 79%. Melting point was not determined due to the potential to combust upon heating.

Synthesis of [oxo(N-benzyl-N,N',N''-tris(2-pyridylmethyl)-1,2-diaminoethane-κ^{5}N)iron(IV) bis(trifluoromethanesulfonato) ([(BnTPEN)Fe(IV)=O](OTf)$_2$)[1]

The iron (IV) compound was prepared immediately before use for kinetics experiments, which first required using a known and calculated molarity of the iron (II) complex to successfully achieve pseudo-first order kinetics conditions. 0.02046 grams (0.025 mmol) of [Fe(II)BnTPEN](OTf)$_2$ was dissolved in 25 mL of acetonitrile that was freshly distilled under nitrogen (in a 25 mL volumetric flask), yielding a 1 mM iron (II) complex solution. 15-20 mL of the resulting iron (II) complex solution was added to an oven dried scintillation vial containing 0.00610 grams (0.027 mmol, 1.1 molar excess) of PhIO and a spin vane. This vial was then stoppered with a rubber septum. The rubber septum was pierced with a cannula connected to an argon gas tank, then another small needle was placed through the septum to serve as a gas outlet. A minimal flow of argon was bubbled into the solution, and sparged for 5-10 minutes before proceeding with kinetic studies. The degassed solution was vigorously stirred to break up PhIO solid in the vial. The initial solution appeared brown-orange in color, within 10 minutes of reaction the color slowly transitioned to faint green. This solution was allowed to stir for 20-30 minutes, after which it was stored in a freezer to prevent decay of our desired iron (IV) product. Yield was not determined; a stoichiometric conversion is assumed for kinetics conditions. Its characteristic UV-Vis absorption at λ_{max} = 739 nm was observed, which is monitored for kinetic measurements.

Rate constants and KIEs

Table S1. The temperature effects on the rate constants and KIEs of the hydride transfer reaction from HEH to $MeMA^+$ in acetonitrile [a,b]

Temp (°C)	k_{2H} ($M^{-1}s^{-1}$)	k_{2D} ($M^{-1}s^{-1}$)	1° KIE
55.0	7.21(0.01)(0.08) x10	1.85(0.02)(0.02) x10	3.90(0.04)(0.05)
45.0	4.93(0.05)(0.05) x10	1.23(0.01)(0.01) x10	4.02(0.05)(0.05)
35.0	3.30(0.02)(0.02) x10	7.73(0.06)(0.07)	4.27(0.04)(0.02)
25.0	2.12(0.01)(0.03) x10	4.77(0.03)(0.05)	4.45(0.03)(0.04)
15.0	1.33(0.01)(0.01) x10	2.76(0.01)(0.05)	4.80(0.03)(0.08)
	ΔE_a = 0.98 (0.09)(0.03)		

[a] Repeated on three different days with 3 repetitions each day. Numbers in the first parenthesis for each value are the pooled standard deviations S(pooled), numbers in the second parathesis are the standard deviations of the three values from three days of measurements; [b] [HEH] = 0.0075 M, [chloranil] = 0.30 mM. Absorbance growth at 436 nm due to $MeMA^+$ was followed for kinetic measurements.

Table S2. The temperature effects on the rate constants and KIEs of the hydride transfer reaction from MeMAH to DDQ in acetonitrile [a,b]

**Temp (°C)**	**k_{2H} ($M^{-1}s^{-1}$)**	**k_{2D} ($M^{-1}s^{-1}$)**	**1° KIE**
45.0	1.78(0.04)(0.10) x10^5	4.67(0.08)(0.21) x10^4	3.82 (0.10)(0.06)
35.0	1.66(0.03)(0.10) x10^5	4.10(0.05)(0.21) x10^4	4.06 (0.10)(0.03)
25.0	1.54(0.01)(0.08) x10^5	3.55(0.02)(0.17) x10^4	4.33 (0.05)(0.03)
15.0	1.40(0.01)(0.08) x10^5	3.04(0.02)(0.15) x10^4	4.60 (0.05)(0.04)
5.0	1.27(0.01)(0.07) x10^5	2.56(0.02)(0.14) x10^4	4.95 (0.06)(0.05)
	ΔE_a = 1.14 (0.05)(0.07)		

[a] Repeated on three different days with 3 repetitions each day. Numbers in the first parenthesis for each value are the pooled standard deviations S(pooled), numbers in the second parathesis are the standard deviations of the three values from three days of measurements; [b] [MeMAH] = 0.66 mM, [DDQ] = 0.044 mM. Absorbance growth at 358 nm due to $MeMA^+$ was followed for kinetic measurements.

Table S3. The temperature effects on the rate constants and KIEs of the hydride transfer reaction from PhMAH to DDQ in acetonitrile [a,b]

**Temp (°C)**	**k_{2H} ($M^{-1}s^{-1}$)**	**k_{2D} ($M^{-1}s^{-1}$)**	**1° KIE**
45.0	1.49(0.03)(0.04) x10^5	3.29(0.03)(0.10) x10^4	4.54 (0.11)(0.22)
35.0	1.38(0.02)(0.04) x10^5	2.81(0.03)(0.11) x10^4	4.91 (0.09)(0.26)
25.0	1.24(0.01)(0.03) x10^5	2.27(0.02)(0.07) x10^4	5.45 (0.07)(0.26)
15.0	1.10(0.03)(0.02) x10^5	1.82(0.01)(0.04) x10^4	6.03 (0.19)(0.21)
5.0	9.45(0.08)(0.05) x10^4	1.46(0.02)(0.04) x10^4	6.49 (0.09)(0.18)
	ΔE_a = 1.62 (0.13)(0.12)		

[a] Repeated on three different days with 3 repetitions each day. Numbers in the first parenthesis for each value are the pooled standard deviations S(pooled), numbers in the second parathesis are the standard deviations of the three values from three days of measurements; [b] [PhMAH] = 0.44 mM, [Chloranil] = 0.044 mM. Absorbance growth at 358 nm due to MA^+ was followed for kinetic measurements.

Table S4. The temperature effects on the rate constants and KIEs of the hydride transfer reaction from MAH to $[(BnTBEN)Fe(IV)=O](OTf)_2$ in acetonitrile [a,b]

**Temp (°C)**	**k_{2H} ($M^{-1}s^{-1}$)**	**k_{2D} ($M^{-1}s^{-1}$)**	**1° KIE**
45.0	5.39(0.19)(0.24) x10^3	4.84(0.17)(0.12) x10^2	11.15(0.58)(0.72)
35.0	3.95(0.17)(0.22) x10^3	2.90(0.03)(0.10) x10^2	13.65(0.62)(0.93)
25.0	2.76(0.03)(0.13) x10^3	1.69(0.02)(0.03) x10^2	16.36(0.26)(0.55)
15.0	1.85(0.05)(0.05) x10^3	9.98(0.20)(0.19) x10^1	18.50(0.61)(0.45)
5.0	1.16(0.04)(0.03) x10^3	5.09(0.05)(0.08) x10^1	22.73(0.74)(0.18)
	ΔE_a = 3.04 (0.23)(0.29)		

[a] Trials were repeated on three different days with six repetitions each day. Numbers in the first parenthesis for each value are the pooled standard deviations S(pooled), numbers in the second parenthesis are the standard deviations of the three average measurements from three separate days; [b] $[Fe(IV)OBnTPEN](OTf)_2$ = 0.5 mM, [MAHH] = 5.0 mM. Absorbance decay was monitored at 739 nm due to consumption of Fe(IV)=O species for kinetic measurements.

Table S5. The temperature effects on the rate constants and KIEs of the hydride transfer reaction from MeMAH to $[(BnTBEN)Fe(IV)=O](OTf)_2$ in acetonitrile [a,b]

**Temp (°C)**	**k_{2H} ($M^{-1}s^{-1}$)**	**k_{2D} ($M^{-1}s^{-1}$)**	**1° KIE**
45.0	5.73(0.13)(0.22) x10^2	7.59(0.07)(0.25) x10^1	7.56(0.20)(0.25)
35.0	3.72(0.03)(0.07) x10^2	3.93(0.05)(0.13) x10^1	9.46(0.17)(0.22)
25.0	2.35(0.05)(0.03) x10^2	2.07(0.03)(0.08) x10^1	11.38(0.31)(0.32)

15.0	1.45(0.05)(0.06) x10^2	9.79(0.34)(0.18) x10^0	14.82(0.75)(0.32)
5.0	8.44(0.15)(0.12) x10^1	4.87(0.29)(0.40) x10^0	17.41(0.96)(1.17)
	ΔE_a = 3.71 (0.14)(0.28)		

[a] Trials were repeated on three different days with six repetitions each day. Numbers in the first parenthesis for each value are the pooled standard deviations S(pooled), numbers in the second parenthesis are the standard deviations of the three average measurements from three separate days; [b] [Fe(IV)OBnTPEN]$(OTf)_2$ = 0.5 mM, [DMAH] = 5.0 mM. Absorbance decay was monitored at 739 nm due to consumption of Fe(IV)=O species for kinetic measurements.

Table S6. The temperature effects on the rate constants and KIEs of the hydride transfer reaction from PhMAH to [(BnTBEN)Fe(IV)=O]$(OTf)_2$ in acetonitrile [a,b]

**Temp (°C)**	k_{2H} **($M^{-1}s^{-1}$)**	k_{2D} **($M^{-1}s^{-1}$)**	**1° KIE**
45.0	3.73(0.05)(0.13) x10^2	6.03(0.07)(0.10) x10^1	6.19(0.12)(0.17)
35.0	2.51(0.08)(0.03) x10^2	2.89(0.03)(0.03) x10^1	8.71(0.28)(0.15)
25.0	1.74(0.12)(0.02) x10^2	1.42(0.04)(0.06) x10^1	12.28(0.43)(0.50)
15.0	1.04(0.05)(0.02) x10^2	6.45(0.24)(0.31) x10^0	16.20(1.04)(0.85)
5.0	6.25(0.29)(0.36) x10^1	2.97(0.17)(0.28) x10^0	21.14(1.76)(2.18)
	ΔE_a = 5.38 (0.35)(0.42)		

[a] Trials were repeated on three different days with three to six repetitions each day. Numbers in the first parenthesis for each value are the pooled standard deviations S(pooled), numbers in the second parenthesis are the standard deviations of the three average measurements from three separate days; [b] [Fe(IV)OBnTPEN]$(OTf)_2$ = 0.5 mM, [PhMAH] = 5.0 mM. Absorbance decay was monitored at 739 nm due to consumption of Fe(IV)=O species for kinetic measurements.

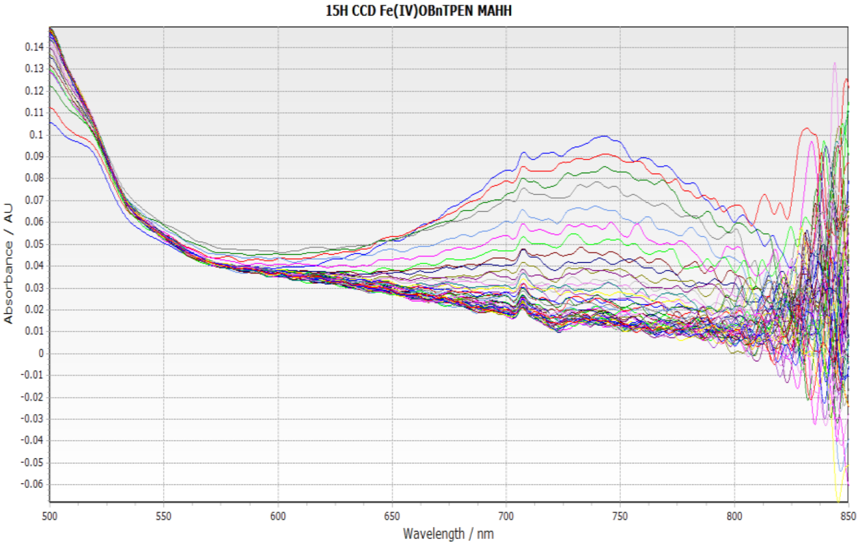

Figure S1. Kinetics CCD scans (using tungsten lamp) of the decay of [(BnTPEN)Fe(IV)=O]$^{2+}$ resulting from reaction with MAH at 15 °C in acetonitrile ([Fe(IV)=O] = 0.50 mM, [MAH] = 5.0 mM).[1]

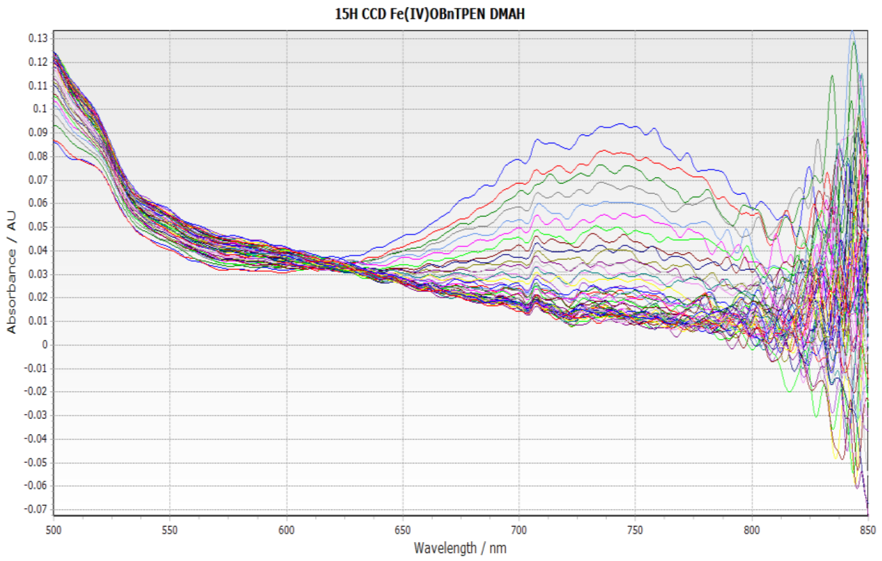

Figure S2. Kinetics CCD scans (using tungsten lamp) of the decay of $[(BnTPEN)Fe(IV)=O]^{2+}$ resulting from reaction with MeMAH at 15 °C in acetonitrile ([Fe(IV)=O] = 0.50 mM, [MAH] = 5.0 mM).[1]

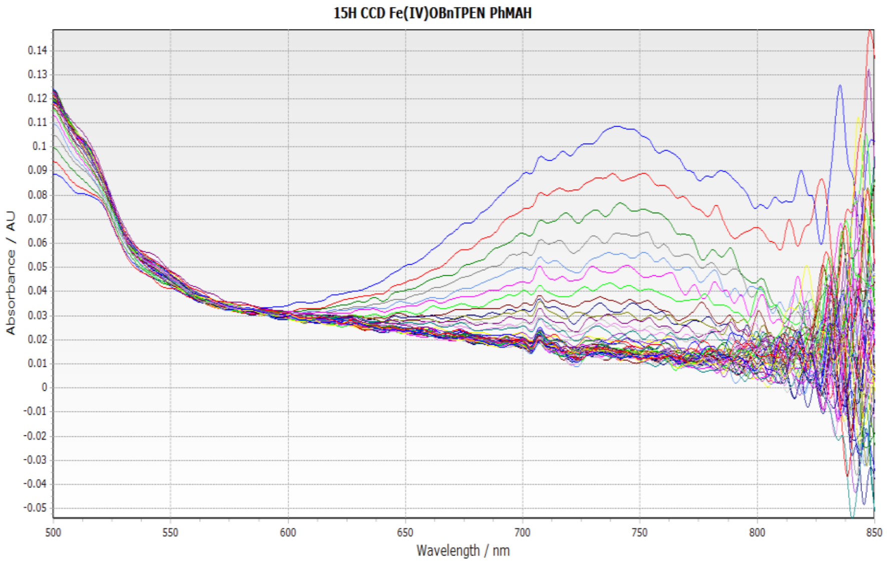

Figure S3. Kinetics CCD scans (using tungsten lamp) of the decay of $[(BnTPEN)Fe(IV)=O]^{2+}$ resulting from reaction with PhMAH at 15 °C in acetonitrile ([Fe(IV)=O] = 0.50 mM, [MAH] = 5.0 mM).[1]

Reference

1. Nicholas DeGroot, Masters Thesis, "*Structural Effects on the Temperature Dependence of Kinetic Isotopic Effects on Multi-Step and Endergonic Hydride Transfer Reactions of NADH Models in Acetonitrile*" Southern Illinois University Edwardsville, United States, January **2026**.

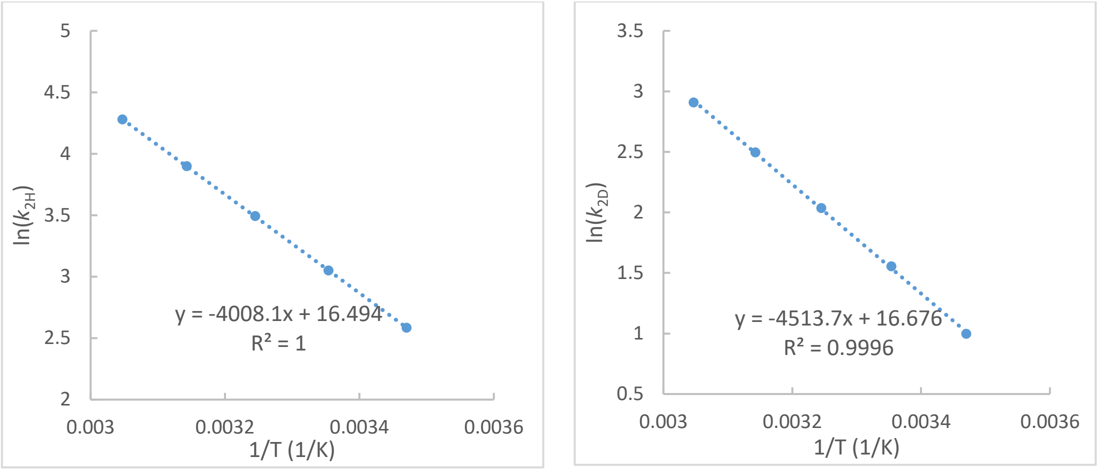

Figure S4. Exemplified Arrhenius plots for hydride (left) and deuteride (right) transfer reactions from HEH and HEH-d_2 (0.0075 M) to $MeMA^+$ (0.30 mM) in acetonitrile (temperatures are 15, 25, 35, 45, and 55 °C, respectively). Data are from Table S1 Day 1 experiment (from the subsequent Data Availability Statement). The same plots for other reactions can be drawn using the data in the Data Availability Statement.

Data Availability Statement

Primary kinetic data for Tables S1-S6 are presented below. We directly copied the original data from the corresponding excel data file. Due to the decimal point place difference in between the two places, data may slightly differ at the last digit of their numbers. Meanwhile, we provide the *Abs* – time (t) data (plots) for the measurements of the pseudo first-order rate constants (k^{pfo}'s).

Primary kinetic data for the rate constants in Table S1

Day 1 data (December 16, 2020)

Pseudo-first-order rate constants

k^{pfo} (s^{-1})

**Temp (°C)**	**Trial H1**	**Trial H2**	**Trial H3**	**Average k_H^{pfo} (s^{-1})**	**Stdev**	**k_{2H} ($M^{-1}s^{-1}$)**	**Stdev [a]**
55	0.5428	0.54265	0.5355	0.540317	0.000112	7.20E+01	0.01499
45	0.36817	0.36626	0.37532	0.369917	0.004776	4.93E+01	0.63678
35	0.24544	0.24849	0.24458	0.24617	0.002055	3.28E+01	0.27396
25	0.1573	0.15792	0.15936	0.158193	0.001057	2.11E+01	0.14091
15	0.09936	0.09951	0.09929	0.099387	0.000112	1.33E+01	0.01499

**Temp (°C)**	**Trial D1**	**Trial D2**	**Trial D3**	**Average k_D^{pfo} (s^{-1})**	**Stdev**	**k_{2D} ($M^{-1}s^{-1}$)**	**Stdev [a]**
55	0.13687	0.13724	0.13778	0.1373	0.000458	1.83E+01	0.06102
45	0.09218	0.09006	0.09077	0.0910	0.001079	1.21E+01	0.14388
35	0.05696	0.05736	0.05804	0.0575	0.000546	7.66E+00	0.07280
25	0.03541	0.03528	0.03561	0.0354	0.000166	4.72E+00	0.02216
15	0.02029	0.02033	0.02025	0.0203	4E-05	2.71E+00	0.00533

[a] = (Stdev(for k^{pfo})/k^{pfo})*k_2

Three kinetic runs together with the averaged data	First-order kinetic fit of the averaged data as an example
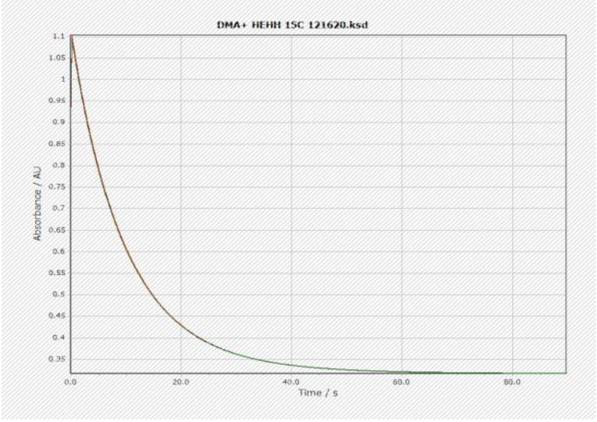	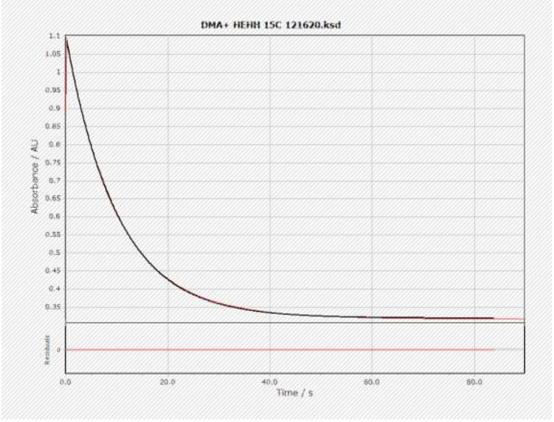
15°C H	

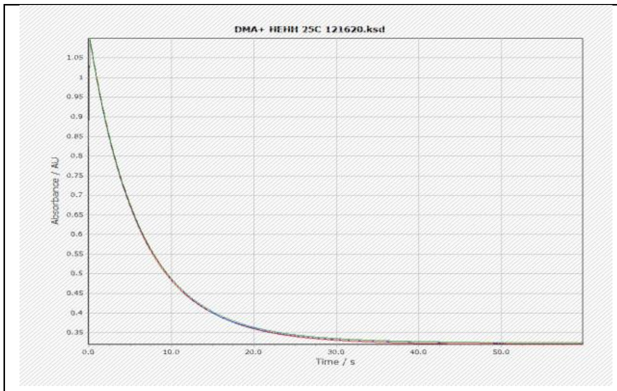
DMA+ HEHH 25C 121620.ksd
Absorbance / AU
Time / s

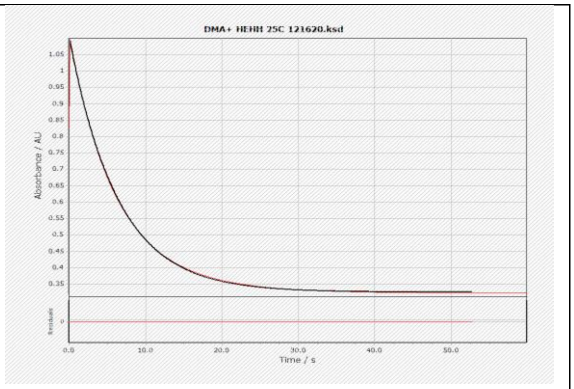
DMA+ HEHH 25C 121620.ksd
Absorbance / AU
Residuals
Time / s

25℃ H

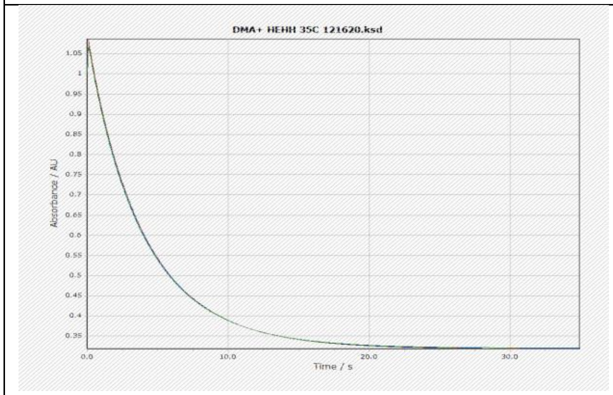
DMA+ HEHH 35C 121620.ksd
Absorbance / AU
Time / s

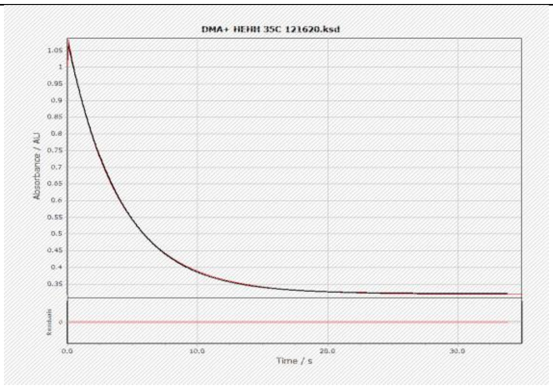
DMA+ HEHH 35C 121620.ksd
Absorbance / AU
Residuals
Time / s

35℃ H

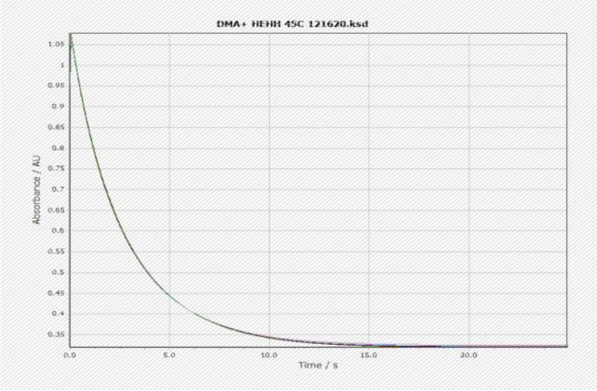
DMA+ HEHH 45C 121620.ksd
Absorbance / AU
Time / s

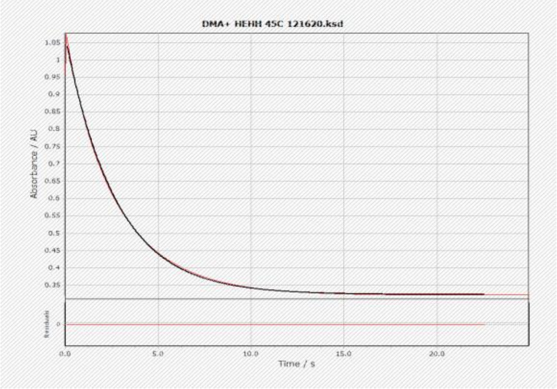
DMA+ HEHH 45C 121620.ksd
Absorbance / AU
Residuals
Time / s

45℃ H

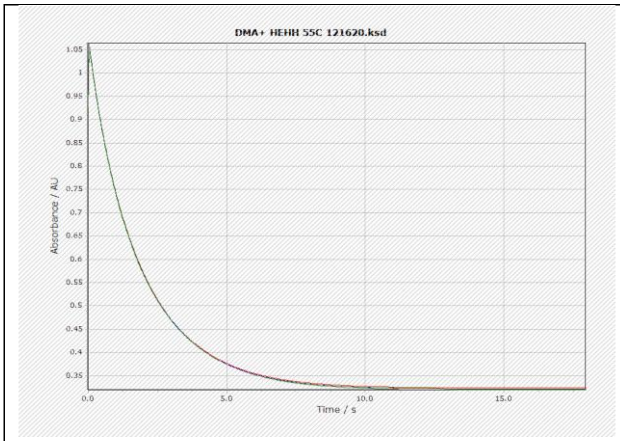
DMA+ HEHH 55C 121620.ksd
Absorbance / AU
Time / s

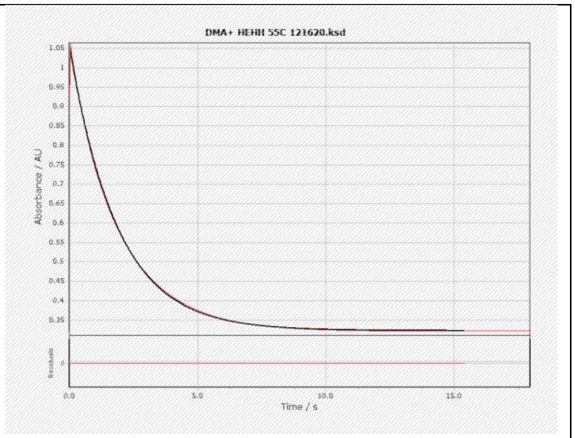
DMA+ HEHH 55C 121620.ksd
Absorbance / AU
Residuals
Time / s

55℃ H

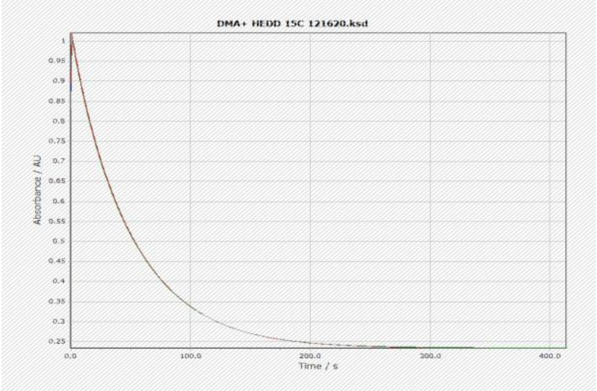
DMA+ HEDD 15C 121620.ksd
Absorbance / AU
Time / s

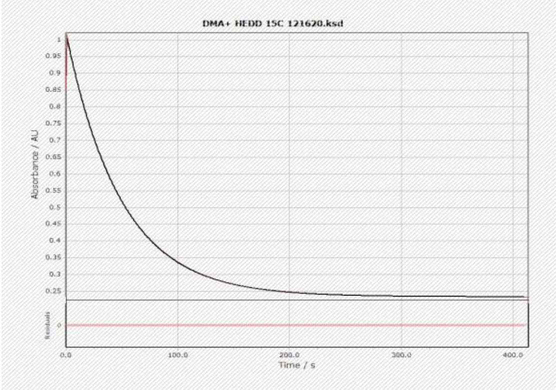
DMA+ HEDD 15C 121620.ksd
Absorbance / AU
Residuals
Time / s

15℃ D

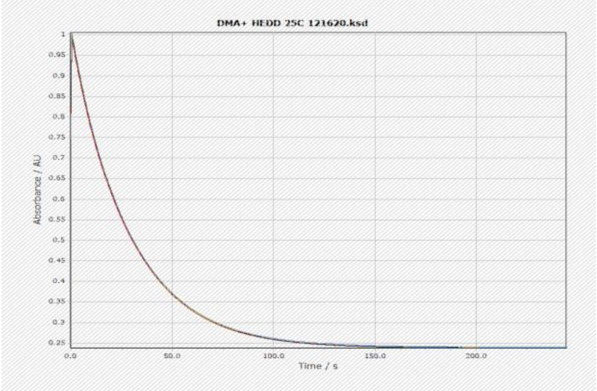
DMA+ HEDD 25C 121620.ksd
Absorbance / AU
Time / s

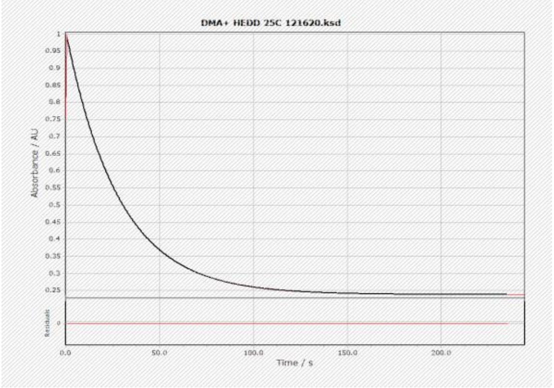
DMA+ HEDD 25C 121620.ksd
Absorbance / AU
Residuals
Time / s

25℃ D

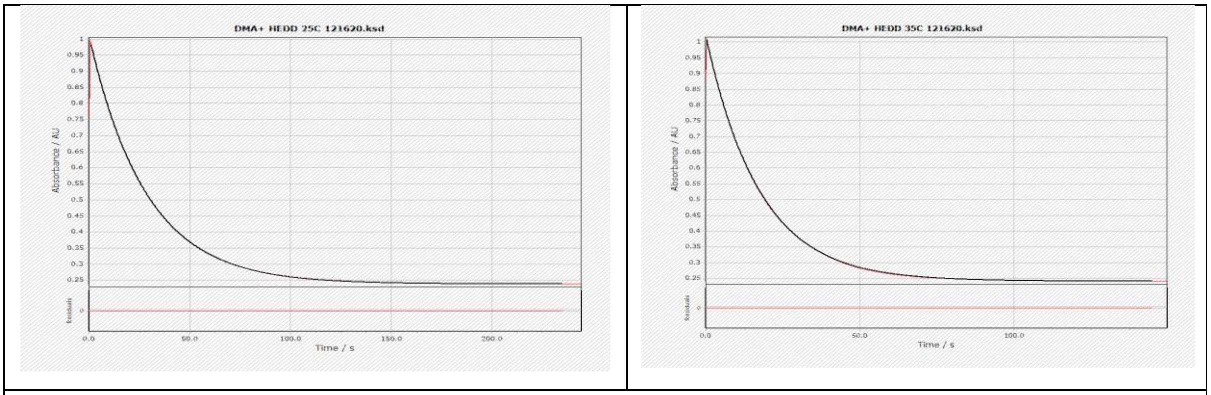

35℃ D

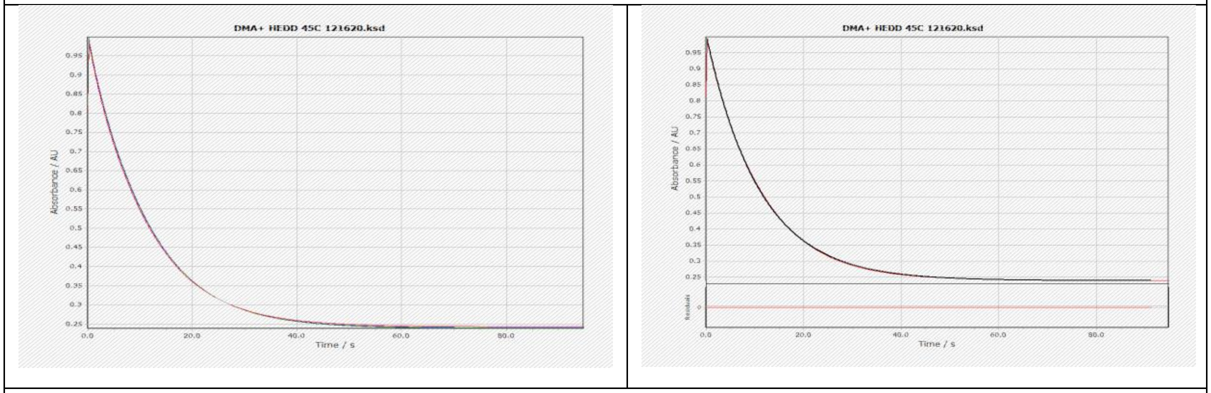

45℃ D

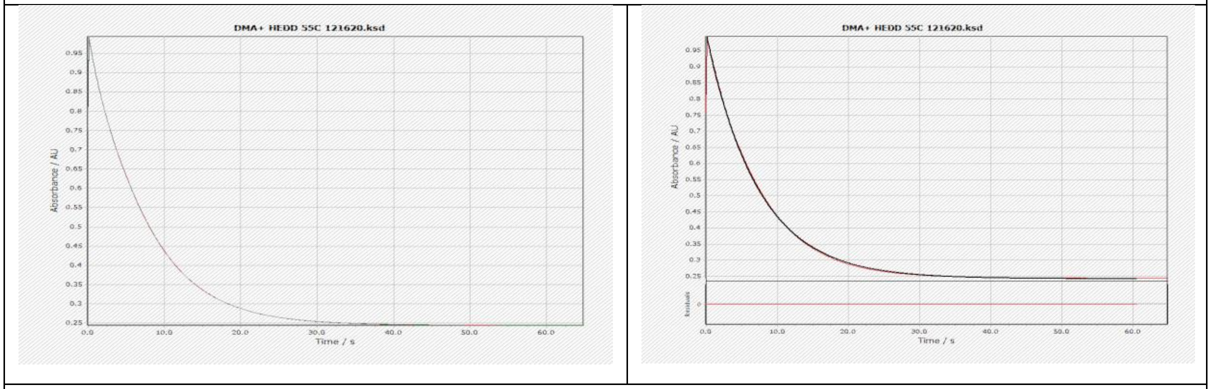

55℃ D

Day 2 data (December 17, 2020)

	Pseudo-first-order rate constants k^{pfo} (s^{-1})						
**Temp (°C)**	**Trial H1**	**Trial H2**	**Trial H3**	**Average k_H^{pfo} (s^{-1})**	**Stdev**	**k_{2H} ($M^{-1}s^{-1}$)**	**Stdev [a]**
55	0.54087	0.53497	0.52962	0.535153	0.00043	7.14E+01	0.05735
45	0.36199	0.36847	0.36777	0.366077	0.003556	4.88E+01	0.47419
35	0.24722	0.24832	0.24731	0.247617	0.000611	3.30E+01	0.08144
25	0.15771	0.15817	0.15817	0.158017	0.000266	2.11E+01	0.03541
15	0.09903	0.09872	0.09957	0.099107	0.00043	1.32E+01	0.05735

Temp (°C)	Trial D1	Trial D2	Trial D3	Average k_D^{pfo} (s^{-1})	Stdev	k_{2D} ($M^{-1}s^{-1}$)	Stdev [a]
55	0.13831	0.13955	0.13935	0.1391	0.000666	1.85E+01	0.08876
45	0.09261	0.09226	0.09173	0.0922	0.000443	1.23E+01	0.05907
35	0.05783	0.05782	0.05829	0.0580	0.000269	7.73E+00	0.03580
25	0.03611	0.03585	0.0358	0.0359	0.000166	4.79E+00	0.02219
15	0.02101	0.02077	0.02096	0.0209	0.000127	2.79E+00	0.01688

[a] = (Stdev(for k^{pfo})/k^{pfo})*k_2

Three kinetic runs together with the averaged data | First-order kinetic fit of the averaged data as an example

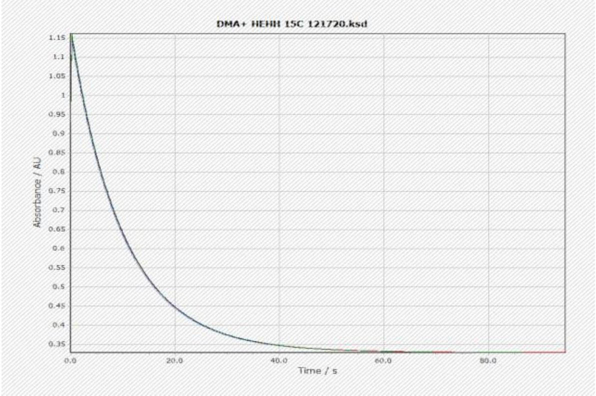

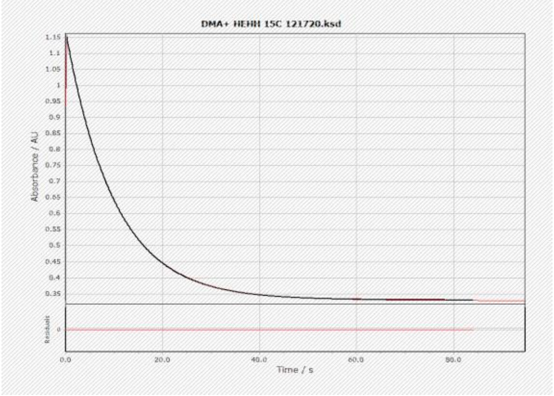

15℃ H

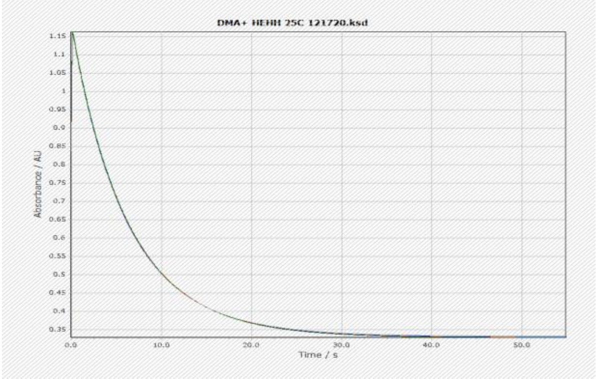

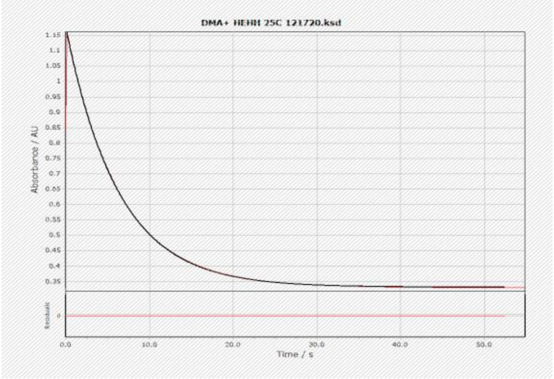

25℃ H

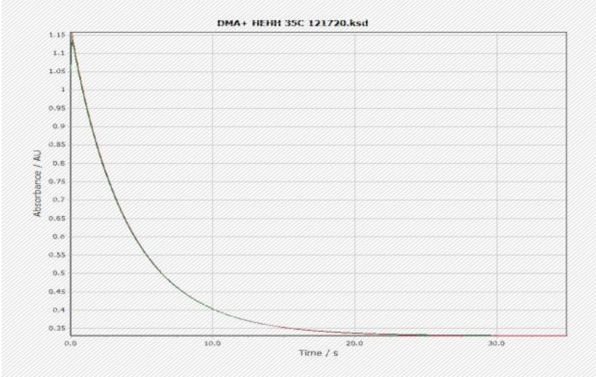

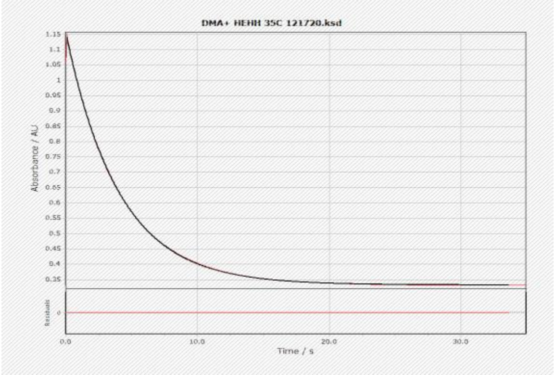

35℃ H

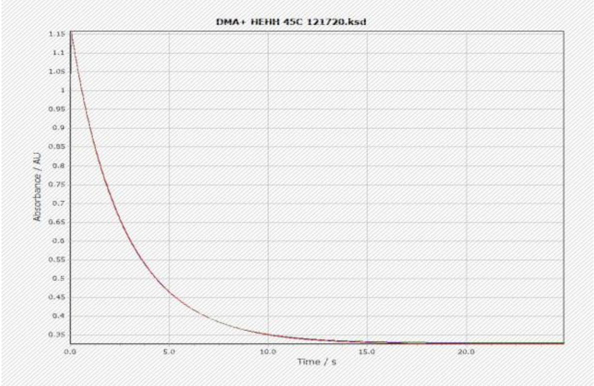

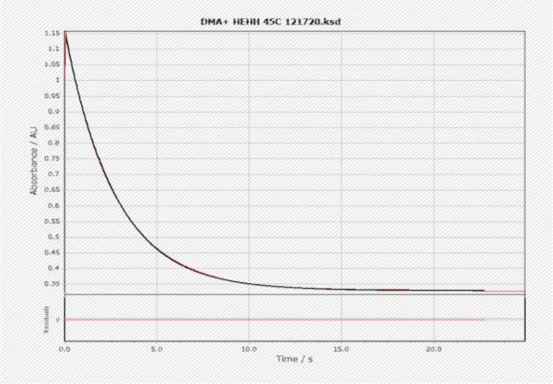

45°C H

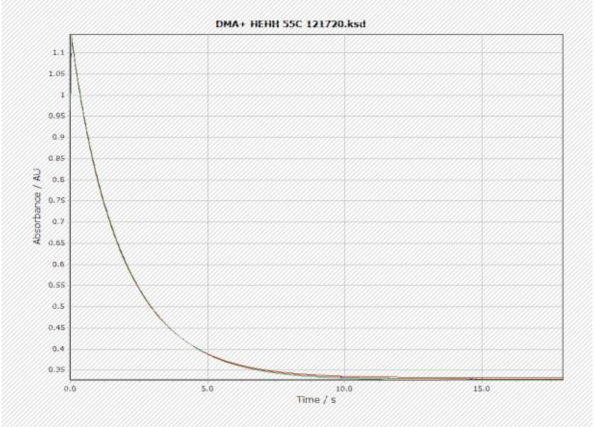

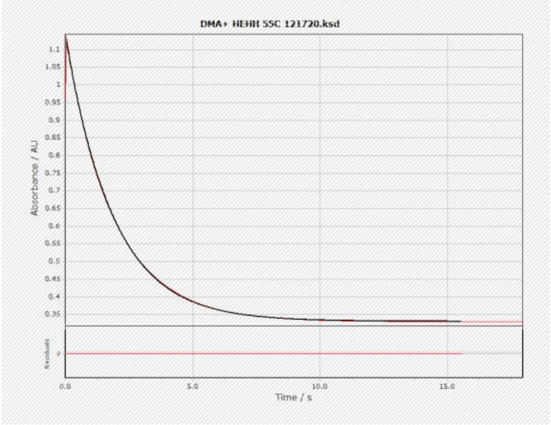

55°C H

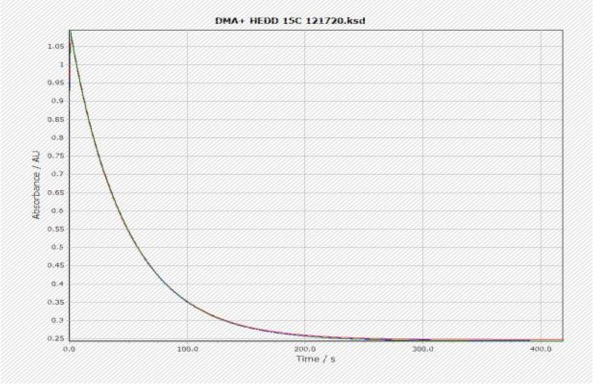

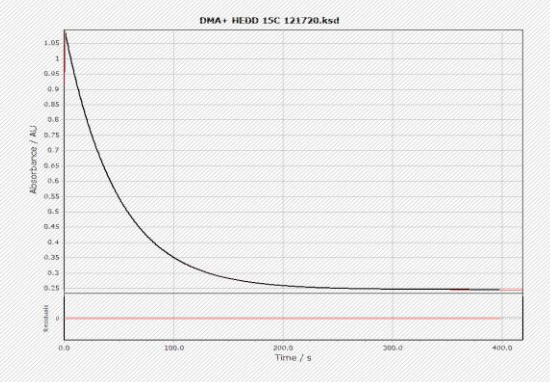

15°C D

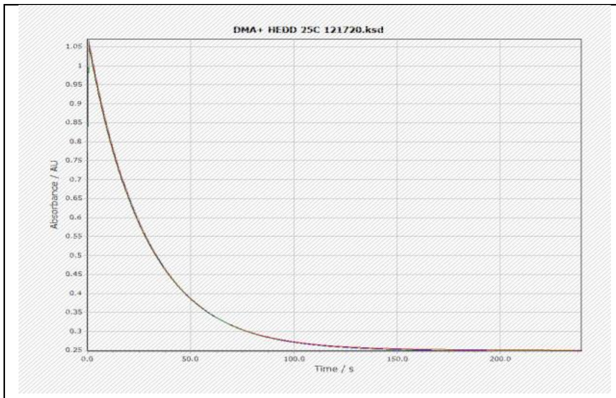

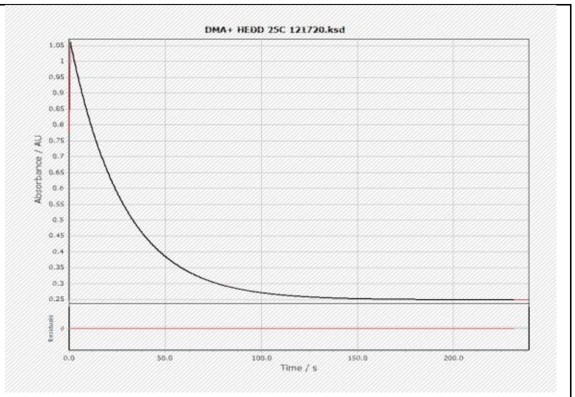

25℃ D

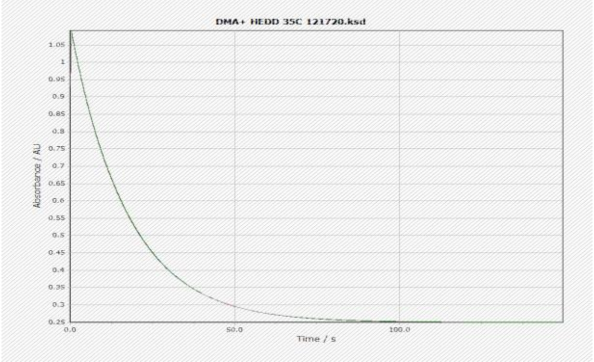

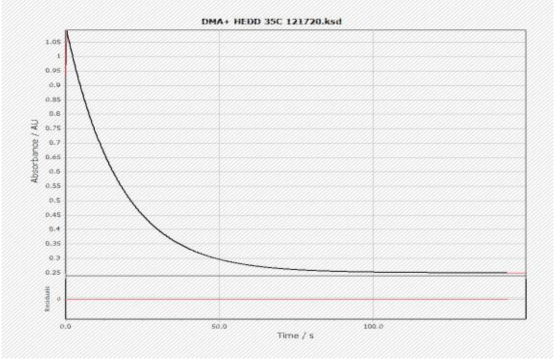

35℃ D

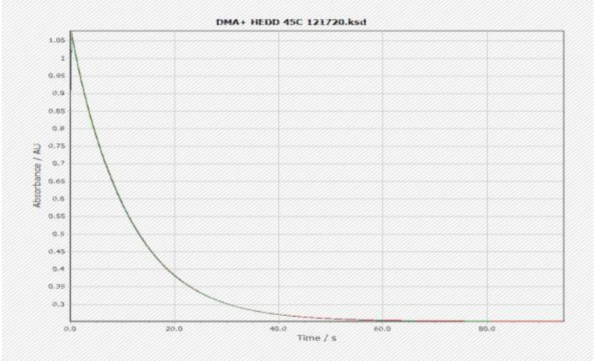

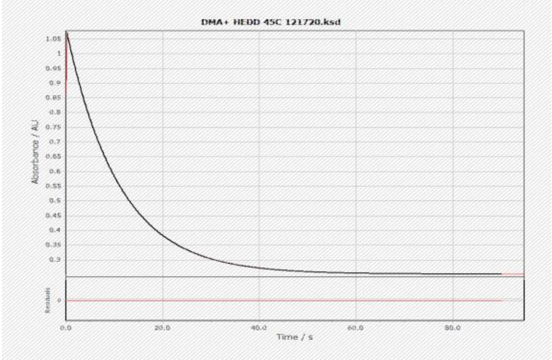

45℃ D

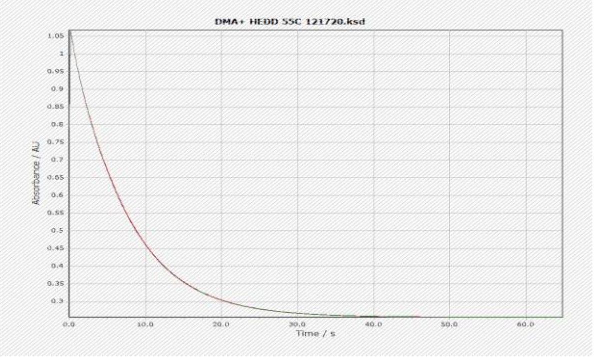

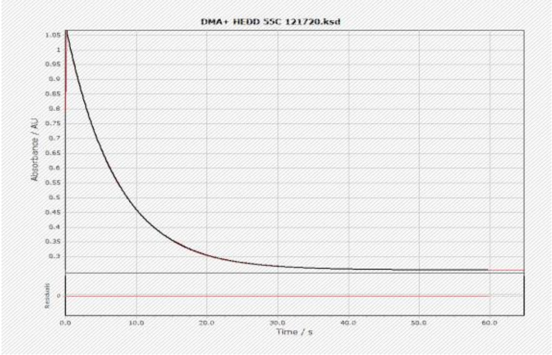

55℃ D

Day 3 data (December 18, 2020)

Temp (°C)	Trial H1	Trial H2	Trial H3	Average k_H^{pfo} (s^{-1})	Stdev	k_{2H} ($M^{-1}s^{-1}$)	Stdev [a]
	Pseudo-first-order rate constants k^{pfo} (s^{-1})						
55	0.54601	0.54714	0.54693	0.540317	0.000112	7.20E+01	0.01499
45	0.37361	0.37056	0.37518	0.369917	0.004776	4.93E+01	0.63678
35	0.24957	0.2465	0.25114	0.24617	0.002055	3.28E+01	0.27396
25	0.16091	0.16089	0.16227	0.158193	0.001057	2.11E+01	0.14091
15	0.09962	0.09925	0.10082	0.099387	0.000112	1.33E+01	0.01499

Temp (°C)	Trial D1	Trial D2	Trial D3	Average k_D^{pfo} (s^{-1})	Stdev	k_{2D} ($M^{-1}s^{-1}$)	Stdev [a]
55	0.13763	0.14176	0.1395	0.1396	0.002068	1.86E+01	0.27574
45	0.09217	0.09265	0.09326	0.0927	0.000546	1.24E+01	0.07284
35	0.05816	0.05855	0.05893	0.0585	0.000385	7.81E+00	0.05133
25	0.03591	0.03641	0.03593	0.0361	0.000283	4.81E+00	0.03774
15	0.02099	0.02091	0.02089	0.0209	5.29E-05	2.79E+00	0.00706

[a] = (Stdev(for k^{pfo})/k^{pfo})*k_2

Three kinetic runs together with the averaged data	First-order kinetic fit of the averaged data as an example
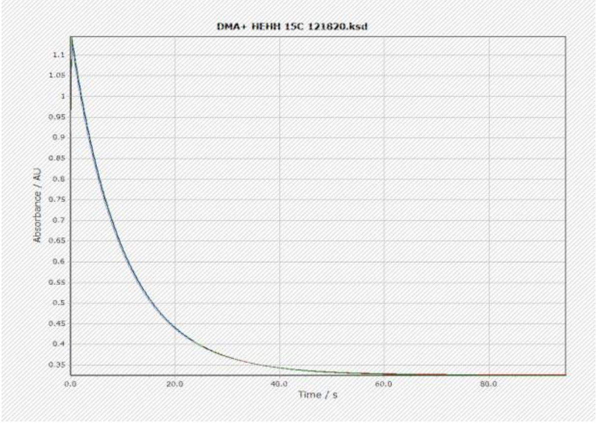	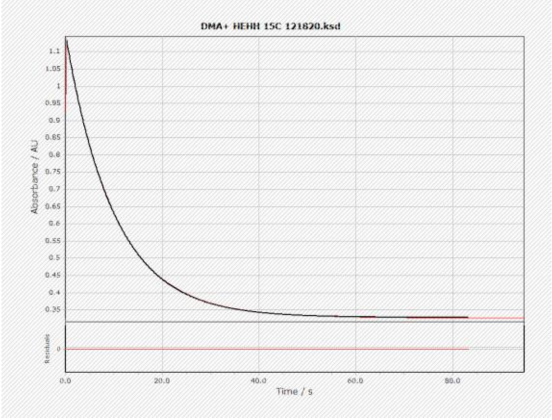
15°C H	
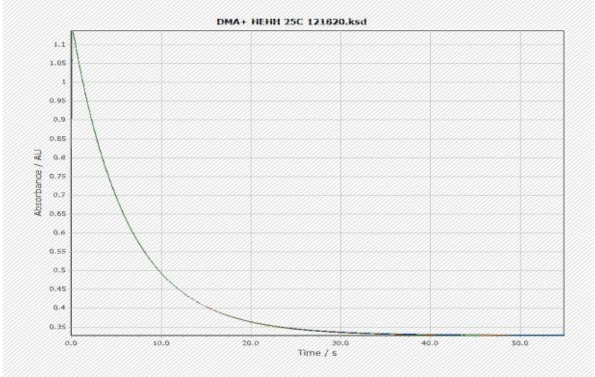	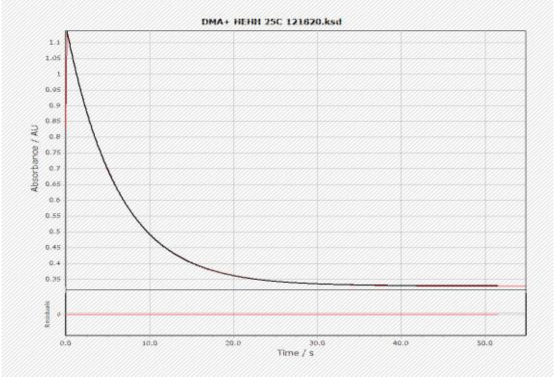
25°C H	

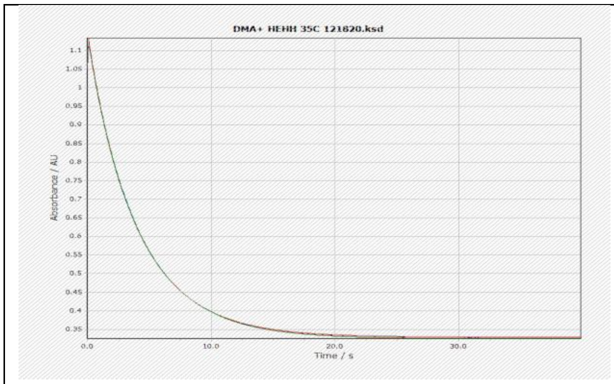
DMA+ HEHH 35C 121620.ksd
Absorbance / AU
Time / s

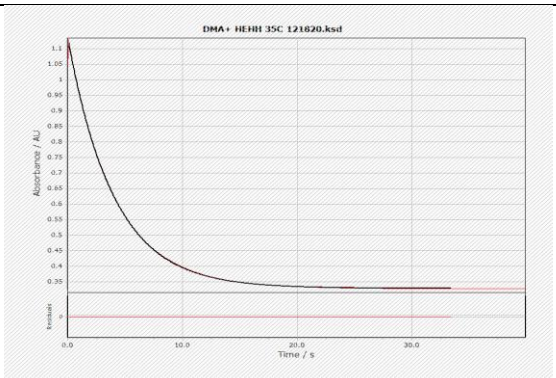
DMA+ HEHH 35C 121620.ksd
Absorbance / AU
Residuals
Time / s

35℃ H

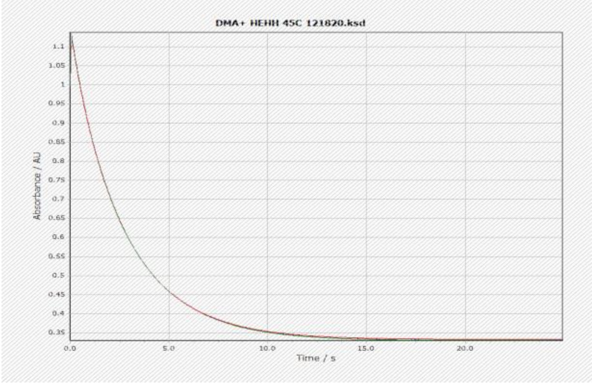
DMA+ HEHH 45C 121620.ksd
Absorbance / AU
Time / s

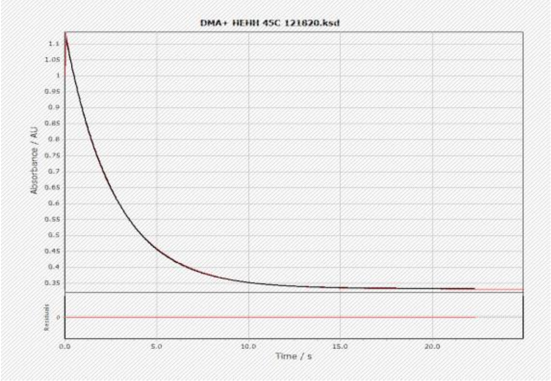
DMA+ HEHH 45C 121620.ksd
Absorbance / AU
Residuals
Time / s

45℃ H

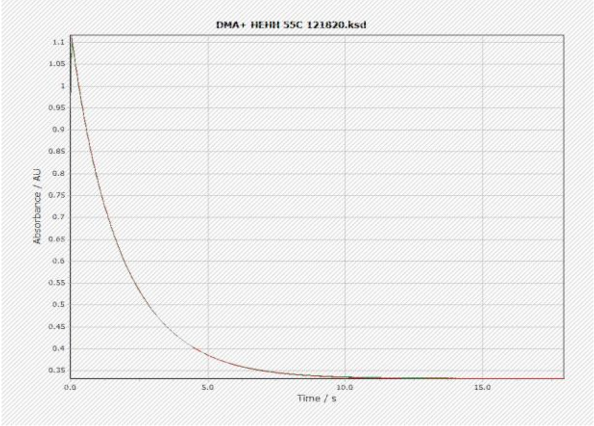
DMA+ HEHH 55C 121620.ksd
Absorbance / AU
Time / s

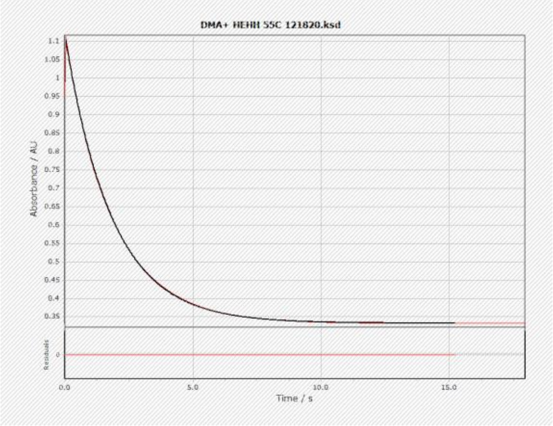
DMA+ HEHH 55C 121620.ksd
Absorbance / AU
Residuals
Time / s

55℃ H

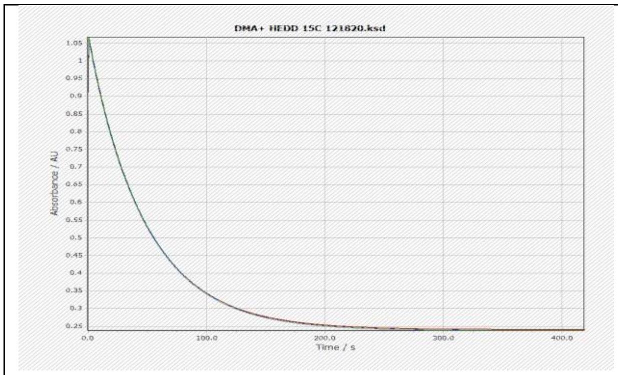

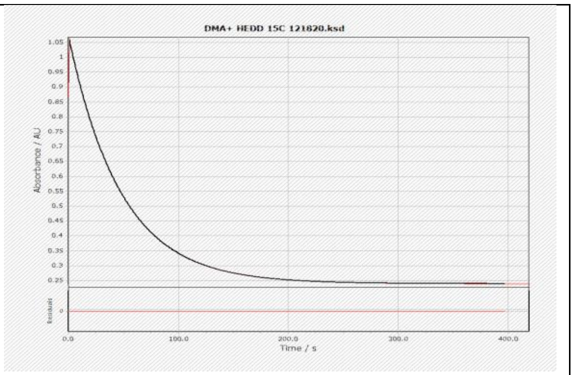

15℃ D

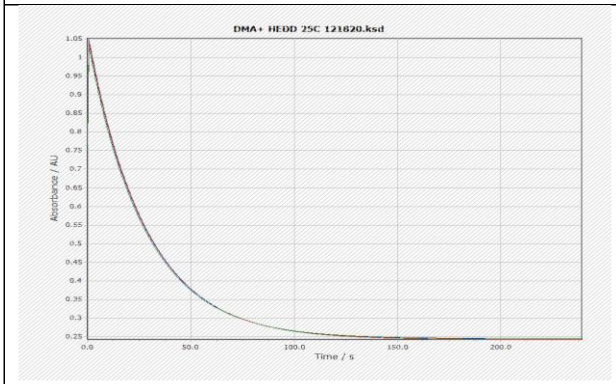

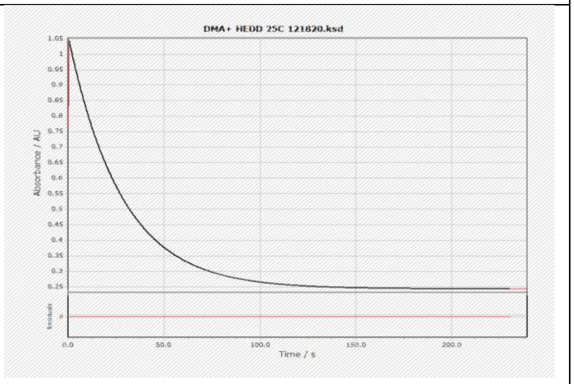

25℃ D

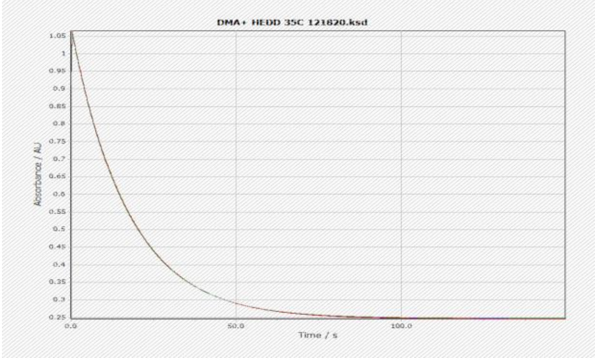

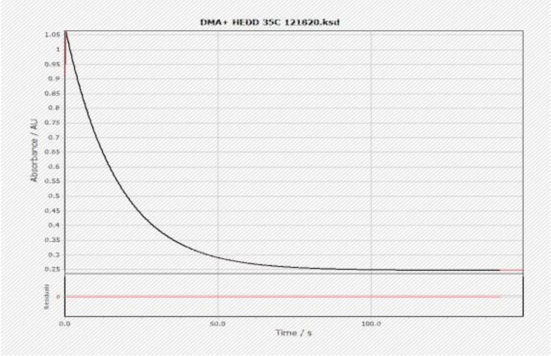

35℃ D

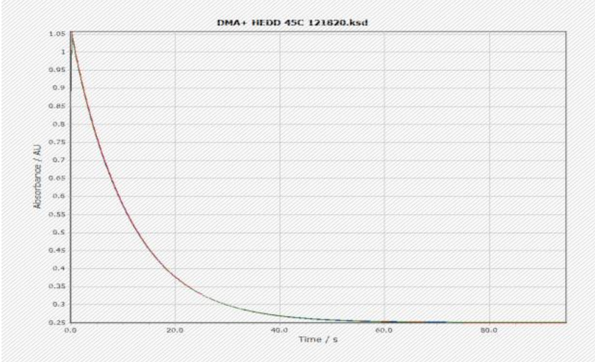

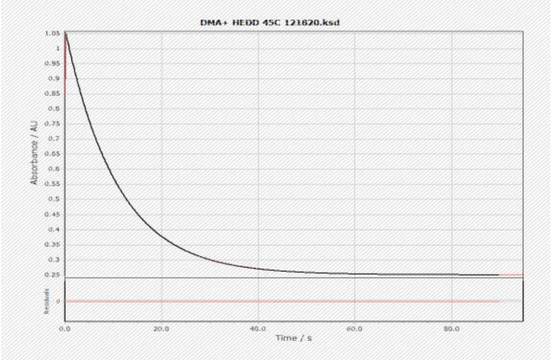

45℃ D

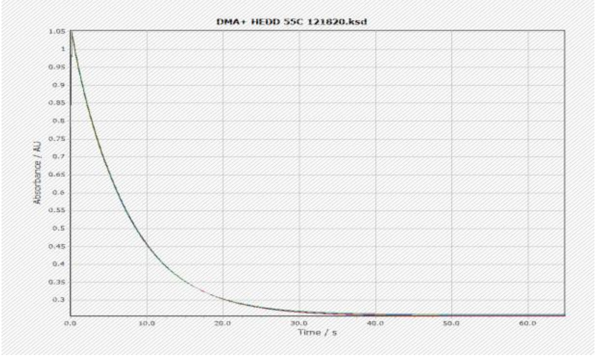

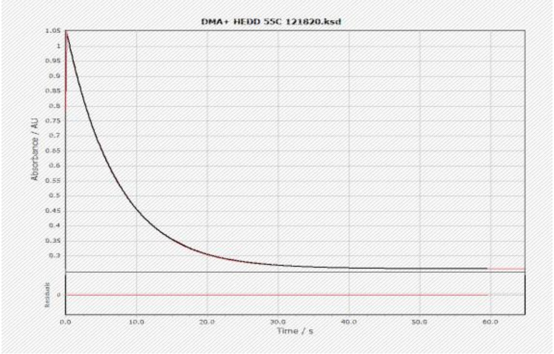

55°C D

Primary kinetic data for the rate constants in Table S2

Day 1 data (February 19, 2024)

	Pseudo-first-order rate constants k^{pfo} (s^{-1})									
**Temp (°C)**	**Trial H1**	**Trial H2**	**Trial H3**	**Trial H4**	**Trial H5**	**Trial H6**	**Average k_H^{pfo} (s^{-1})**	**Stdev**	**k_{2H} ($M^{-1}s^{-1}$)**	**Stdev [a]**
45	125.575	121.775	127.973	123.938	127.243	123.207	124.9521	2.40527	1.89E+05	3644.35450
35	115.982	114.217	123.188	115.575	113.455	118.136	116.7592	3.53856	1.77E+05	5361.46301
25	105.320	107.224	108.624	107.563	106.413	108.681	107.3045	1.29940	1.63E+05	1968.80009
15	98.5450	99.0775	98.241	98.918	98.801	97.977	98.5937	0.42162	1.49E+05	638.82667
5	89.2932	87.7573	89.1228	89.110	89.576	87.811	88.77873	0.78852	1.35E+05	1194.72952
**Temp (°C)**	**Trial D1**	**Trial D2**	**Trial D3**	**Trial D4**	**Trial D5**	**Trial D6**	**Average k_D^{pfo} (s^{-1})**	**Stdev**	**k_{2D} ($M^{-1}s^{-1}$)**	**Stdev [a]**
45	31.887	33.280	32.459	32.163	31.251	31.919	32.16042	0.6786	4.87E+04	1028.20048
35	28.382	28.150	28.152	28.889	28.911	28.869	28.55931	0.3722	4.33E+04	564.02736
25	24.610	24.867	24.740	24.676	24.669	24.948	24.75205	0.1298	3.75E+04	196.75136
15	21.315	21.473	21.216	21.078	21.394	20.973	21.24216	0.1907	3.22E+04	289.01657
5	17.886	18.067	17.872	17.985	17.924	17.947	17.94722	0.0715	2.72E+04	108.45737

[a] = (Stdev(for k^{pfo})/k^{pfo})*k_{2H}

Six kinetic runs together with the averaged data	First-order kinetic fit of the averaged data as an example
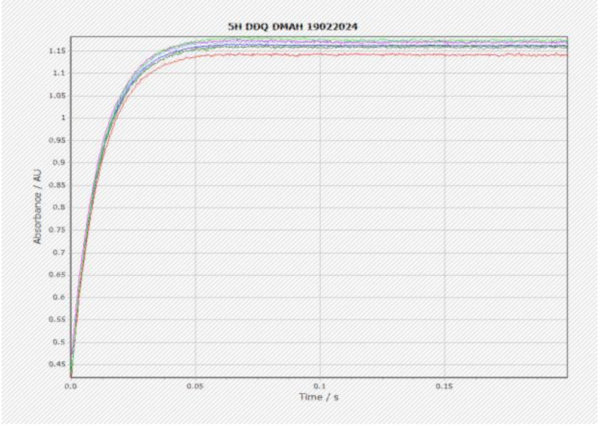	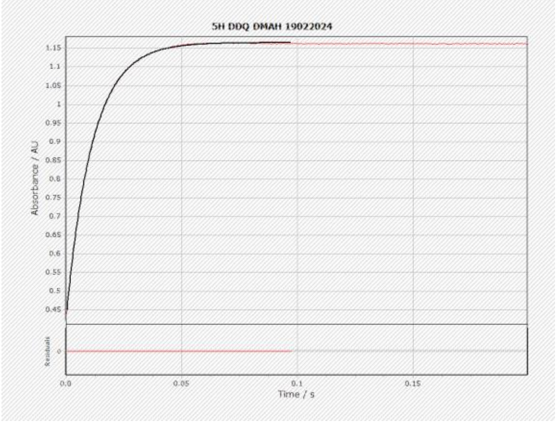

5°C H

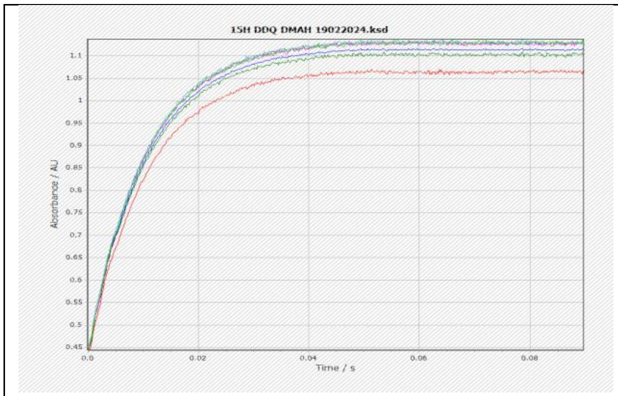
15H DDQ DMAH 19022024.ksd
Absorbance / AU
Time / s

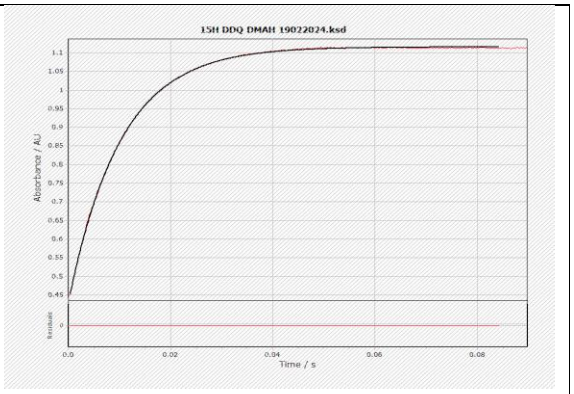
15H DDQ DMAH 19022024.ksd
Absorbance / AU
Residuals
Time / s

15℃ H

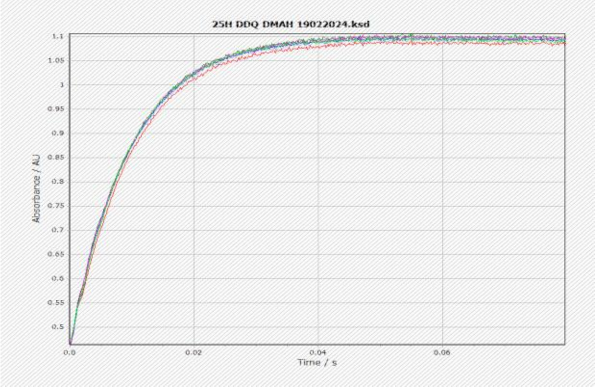
25H DDQ DMAH 19022024.ksd
Absorbance / AU
Time / s

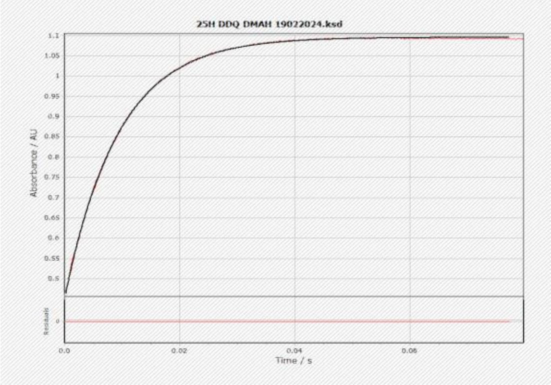
25H DDQ DMAH 19022024.ksd
Absorbance / AU
Residuals
Time / s

25℃ H

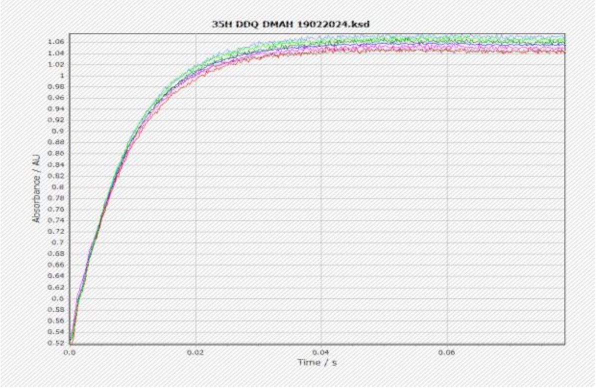
35H DDQ DMAH 19022024.ksd
Absorbance / AU
Time / s

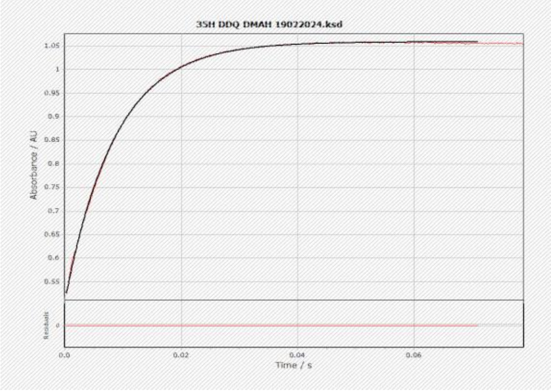
35H DDQ DMAH 19022024.ksd
Absorbance / AU
Residuals
Time / s

35℃ H

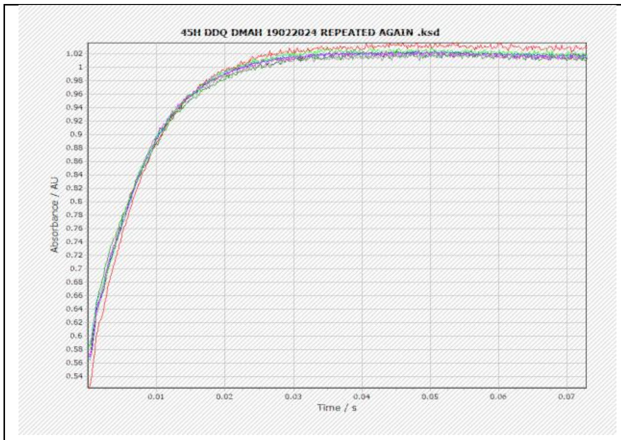

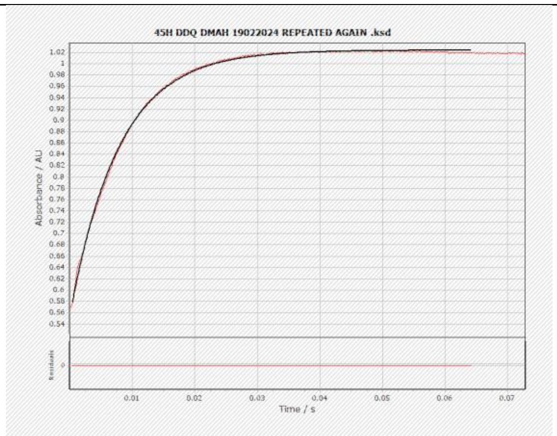

45°C H

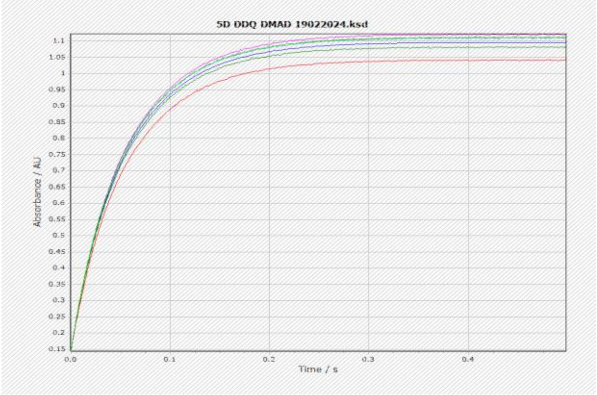

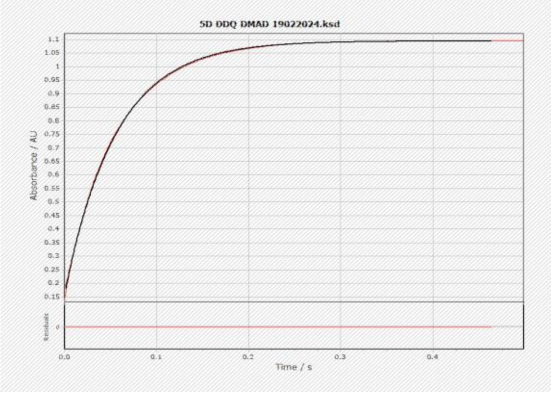

5°C D

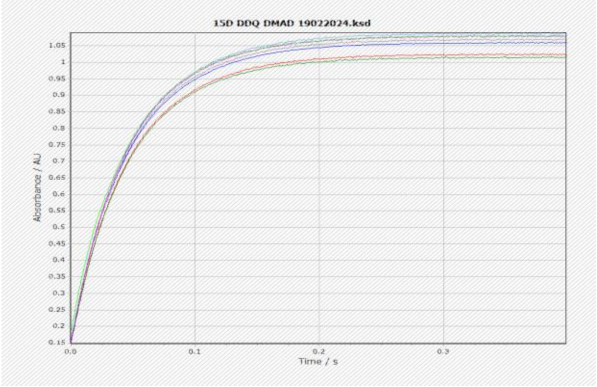

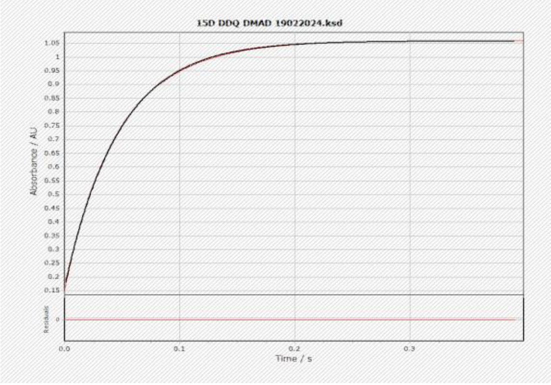

15°C D

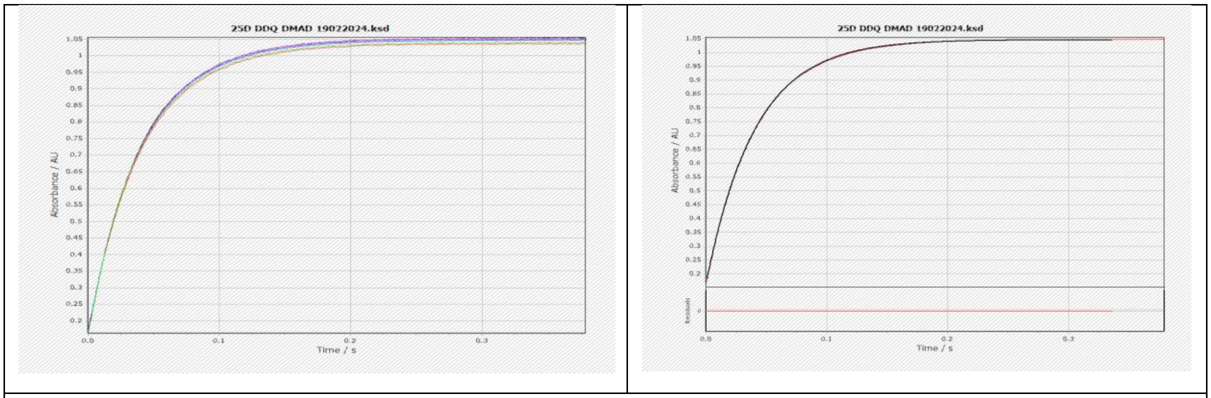

25°C D

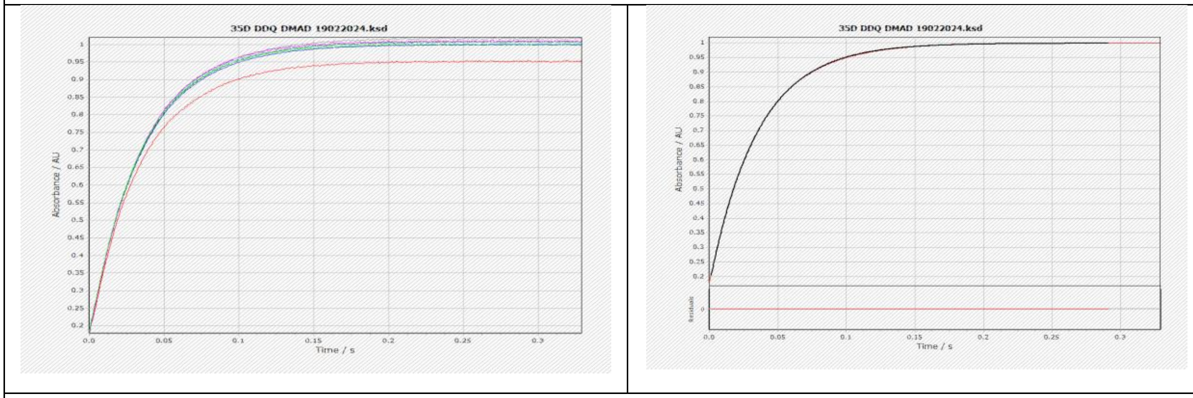

35°C D

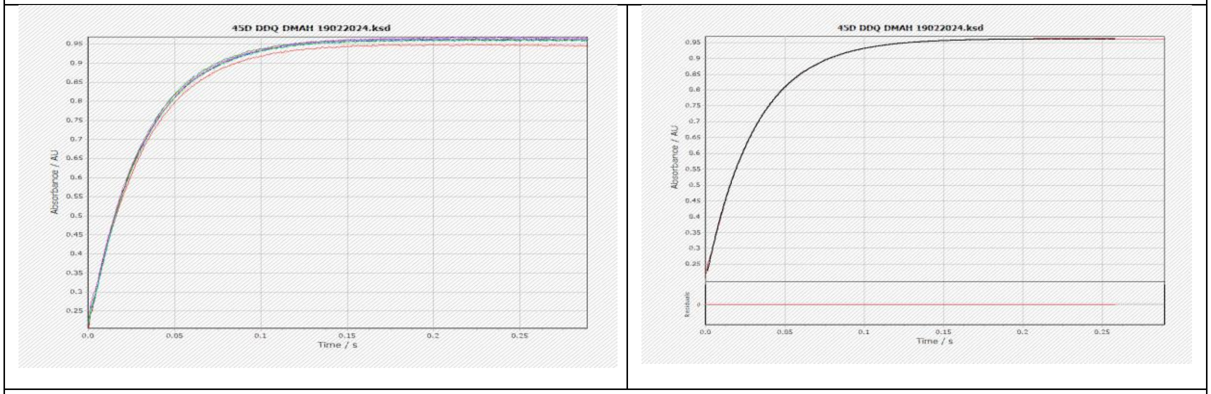

45°C D

Day 2 data (February 23, 2024)

	Pseudo-first-order rate constants									
	k^{pfo} (s^{-1})									
**Temp (°C)**	**Trial H1**	**Trial H2**	**Trial H3**	**Trial H4**	**Trial H5**	**Trial H6**	**Average k_H^{pfo} (s^{-1})**	**Stdev**	**k_{2H} ($M^{-1}s^{-1}$)**	**Stdev [a]**
45	79.079	76.826	77.547	78.733	77.540	75.720	1.76E+05	2803.457	12.07998	20.56952
35	72.554	72.235	72.754	71.441	74.208	70.557	1.64E+05	2818.408	12.00945	17.79677
25	66.641	67.363	66.222	67.159	66.800	66.762	1.52E+05	910.6187	11.93081	15.31902
15	59.047	60.398	60.812	60.786	60.357	60.797	1.37E+05	1542.264	11.82917	13.11097
5	55.983	55.378	54.812	54.932	55.286	55.406	1.26E+05	941.088	11.74151	11.04951

**Temp (°C)**	**Trial D1**	**Trial D2**	**Trial D3**	**Trial D4**	**Trial D5**	**Trial D6**	**Average k_D^{pfo} (s^{-1})**	**Stdev**	**k_{2D} ($M^{-1}s^{-1}$)**	**Stdev [a]**
45	20.620	20.162	20.294	20.837	20.894	20.607	20.56952	0.290898	4.67E+04	661.13177
35	17.560	17.598	18.071	17.775	17.984	17.789	17.79677	0.203232	4.04E+04	461.88981
25	15.322	15.284	15.558	15.262	15.319	15.165	15.31902	0.130827	3.48E+04	297.33488
15	13.138	13.042	12.968	13.307	13.082	13.126	13.11097	0.114564	2.98E+04	260.37365
5	10.893	11.076	11.063	11.122	11.092	11.048	11.04951	0.080792	2.51E+04	183.61929

[a] = (Stdev(for k^{pfo})/k^{pfo})*k_{2H}

Six kinetic runs together with the averaged data	First-order kinetic fit of the averaged data as an example
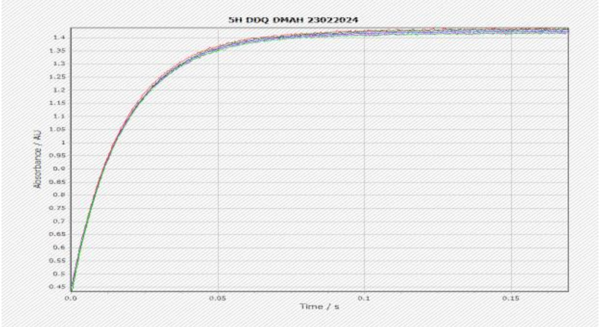	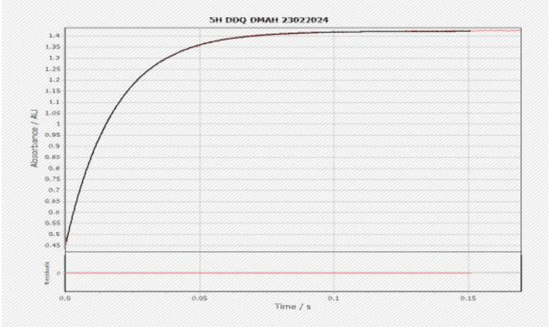
5℃ H	
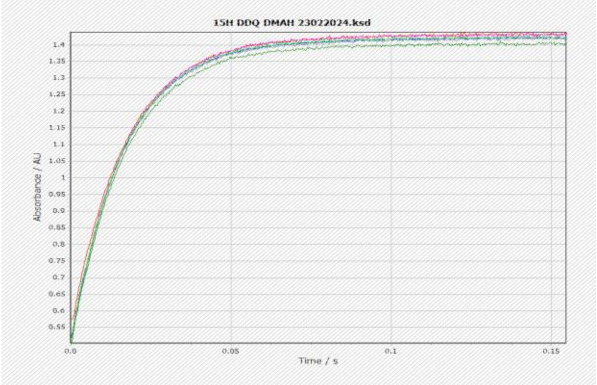	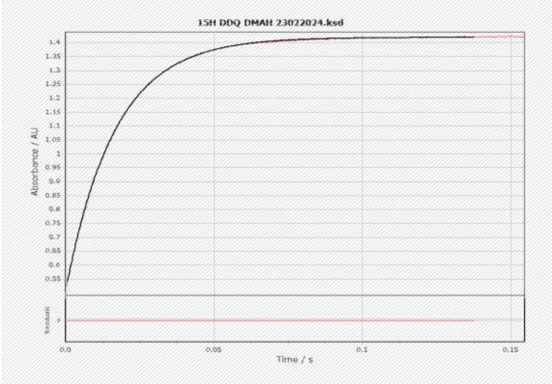
15℃ H	
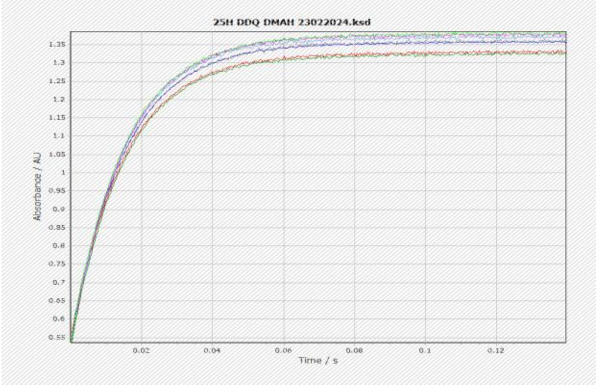	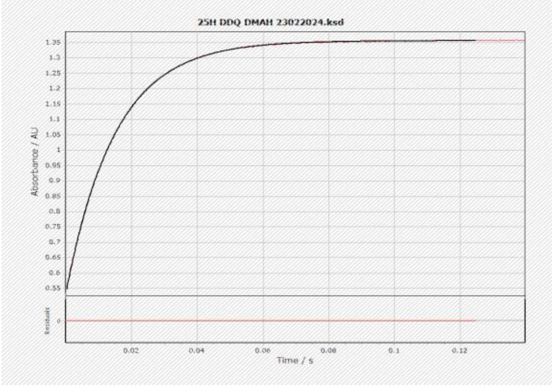
25℃ H	

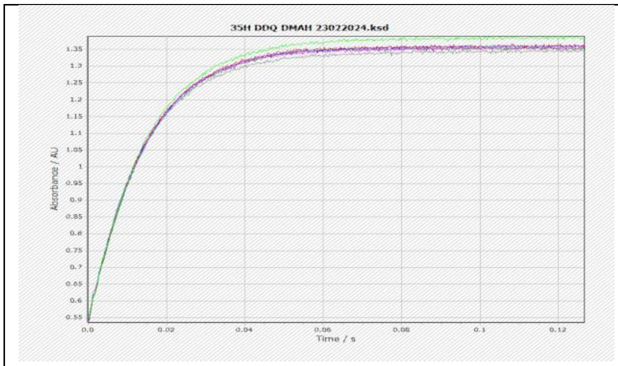

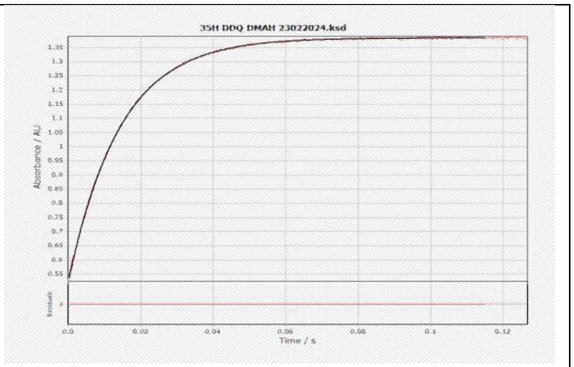

35℃ H

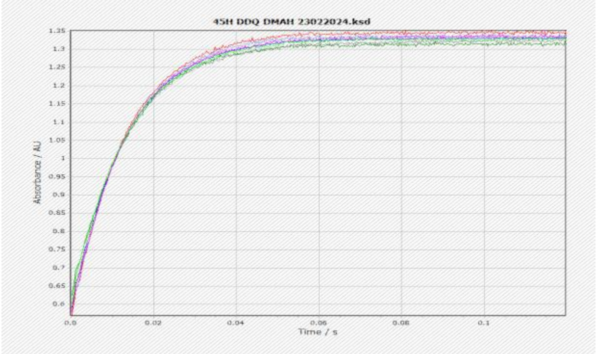

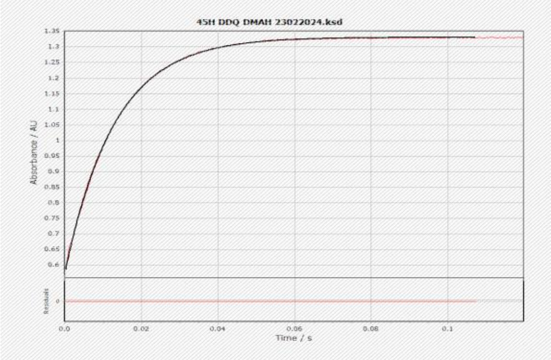

45℃ H

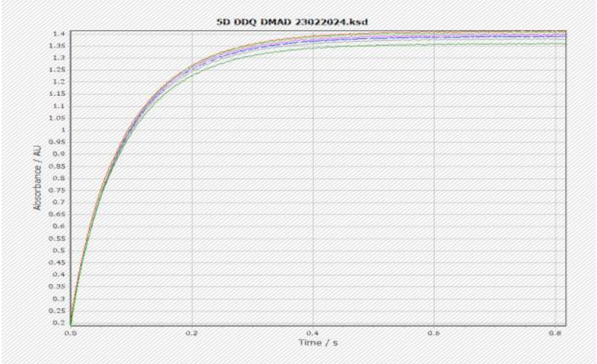

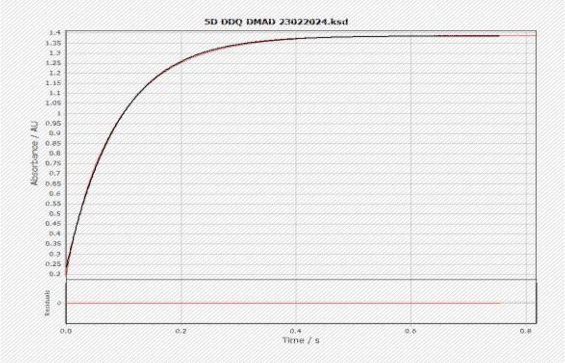

5℃ D

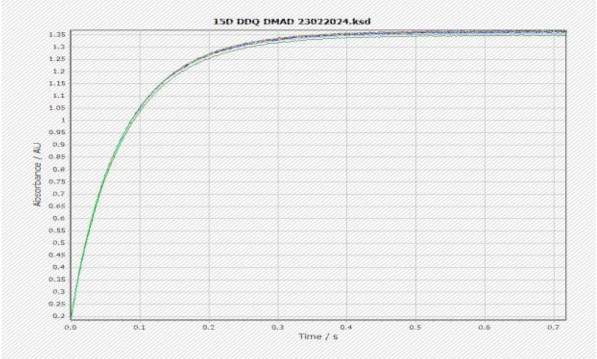

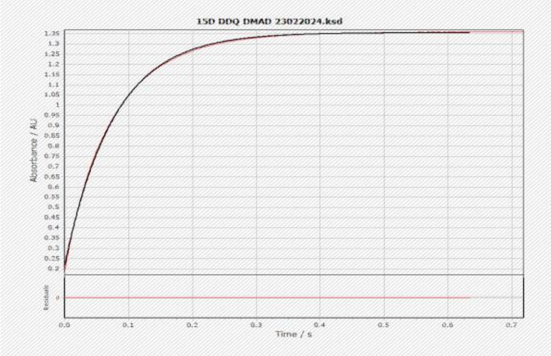

15℃ D

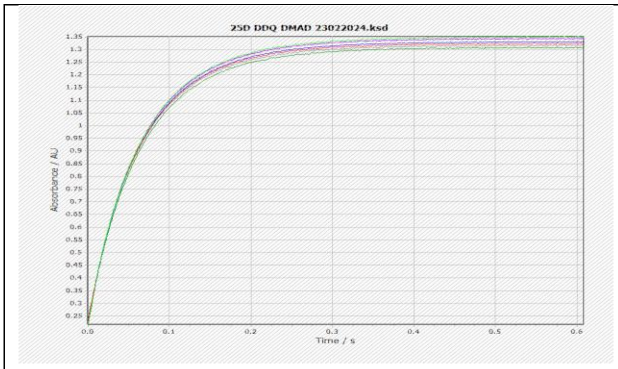

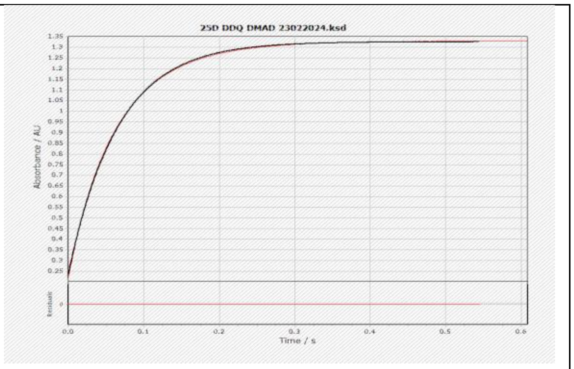

25℃ D

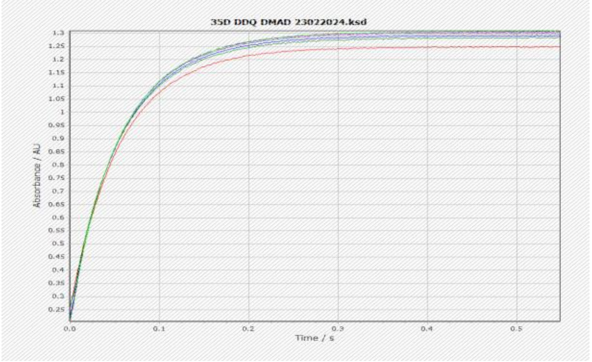

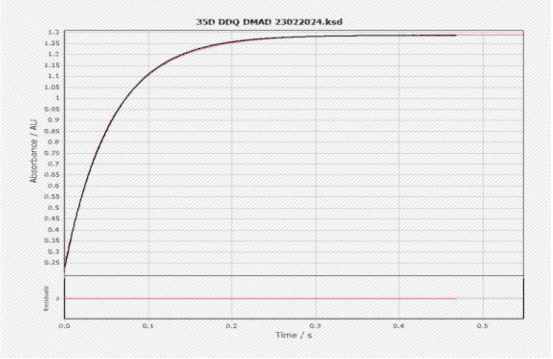

35℃ D

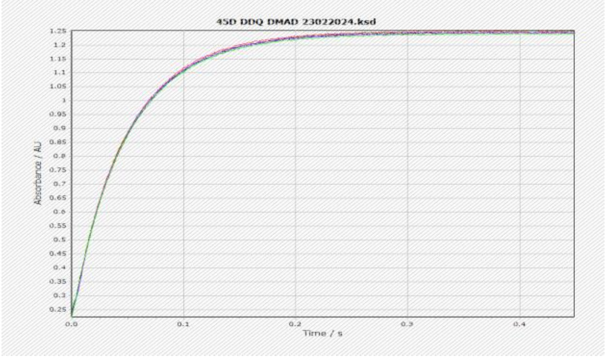

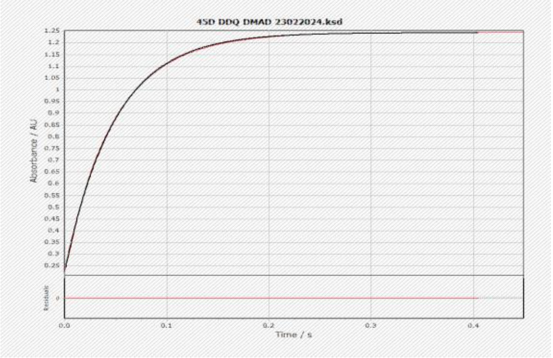

45℃ D

Day 3 data (February 26, 2024)

	Pseudo-first-order rate constants k^{pfo} (s^{-1})									
**Temp (°C)**	**Trial H1**	**Trial H2**	**Trial H3**	**Trial H4**	**Trial H5**	**Trial H6**	**Average k_H^{pfo} (s^{-1})**	**Stdev**	**k_{2H} ($M^{-1}s^{-1}$)**	**Stdev [a]**
45	77.461	75.571	72.166	74.715	74.2171	71.604	74.28941	2.172957	1.69E+05	4938.53813
35	69.797	68.642	69.409	69.668	69.3972	69.700	69.43603	0.420912	1.58E+05	956.61819
25	65.741	64.797	64.451	64.078	64.8360	64.853	64.79298	0.553505	1.47E+05	1257.96693
15	59.199	58.771	59.569	58.623	58.5140	58.122	58.80001	0.514789	1.34E+05	1169.97560
5	52.762	53.470	52.763	53.380	53.7382	52.245	53.06013	0.560683	1.21E+05	1274.27895

Temp (°C)	Trial D1	Trial D2	Trial D3	Trial D4	Trial D5	Trial D6	Average k_D^{pfo} (s^{-1})	Stdev	k_{2D} ($M^{-1}s^{-1}$)	Stdev [a]
45	19.817	19.818	19.516	19.469	19.573	19.287	19.58065	0.207508	4.45E+04	471.60986
35	16.972	17.318	17.165	17.225	17.556	17.382	17.27005	0.19922	3.93E+04	452.77328
25	15.017	15.120	15.007	15.063	15.151	15.099	15.07645	0.057479	3.43E+04	130.63390
15	13.012	12.831	12.934	12.859	12.817	12.850	12.88419	0.074775	2.93E+04	169.94289
5	11.019	10.762	10.902	10.804	10.777	10.723	10.83170	0.110136	2.46E+04	250.30886

[a] = (Stdev(for k^{pfo})/k^{pfo})*k_{2H}

Six kinetic runs together with the averaged data	First-order kinetic fit of the averaged data as an example

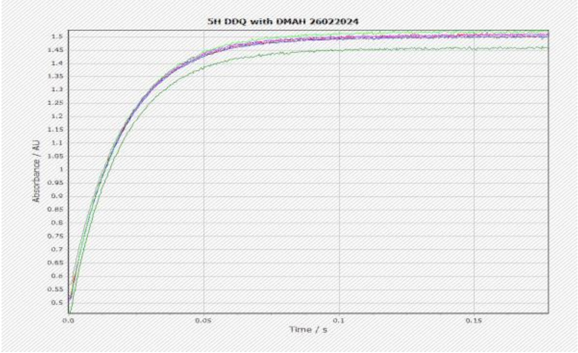

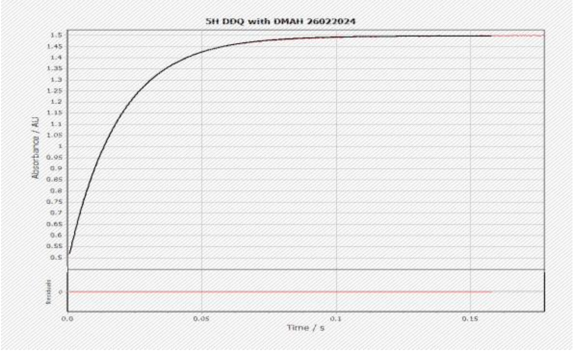

5℃ H

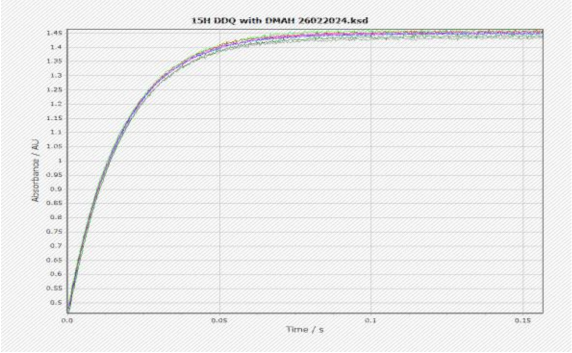

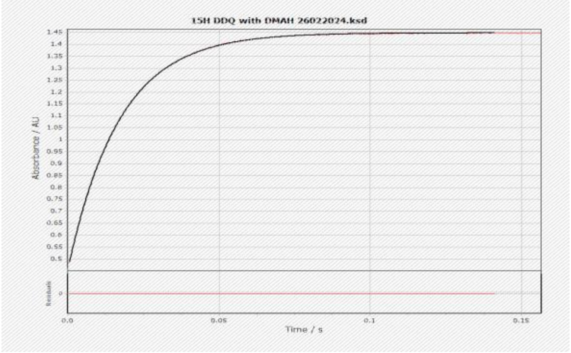

15℃ H

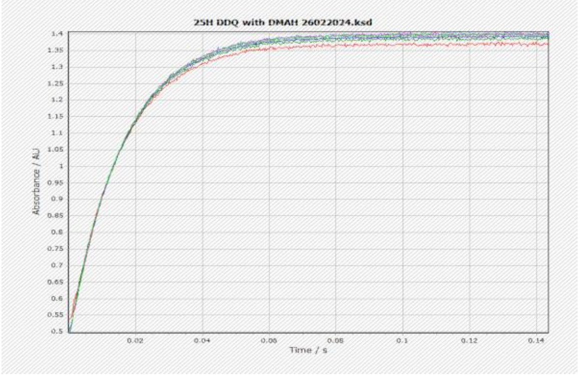

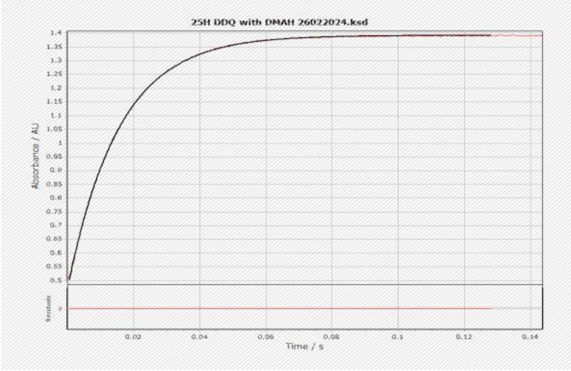

25℃ H

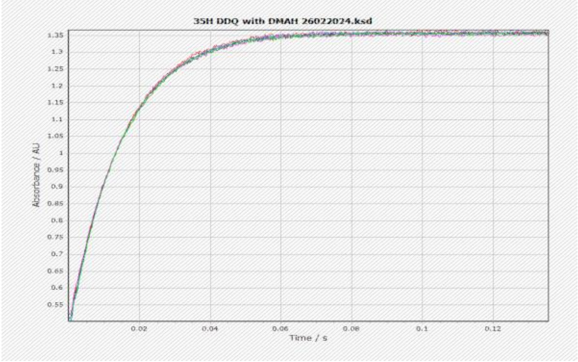

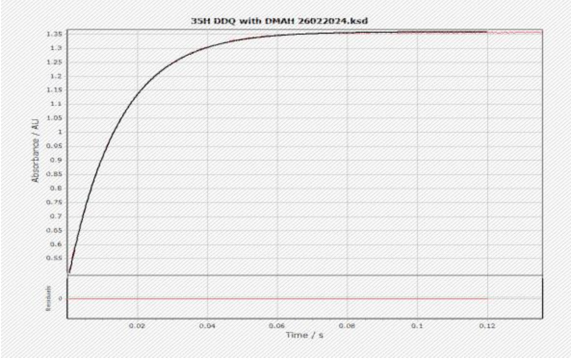

35°C H

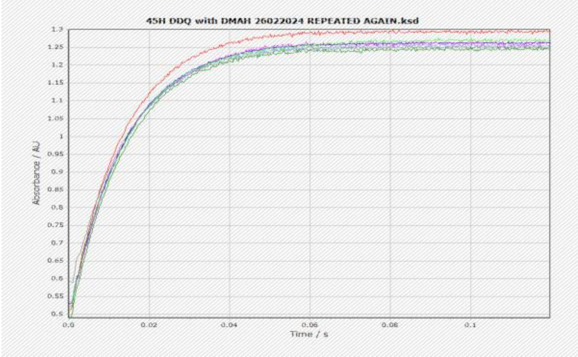

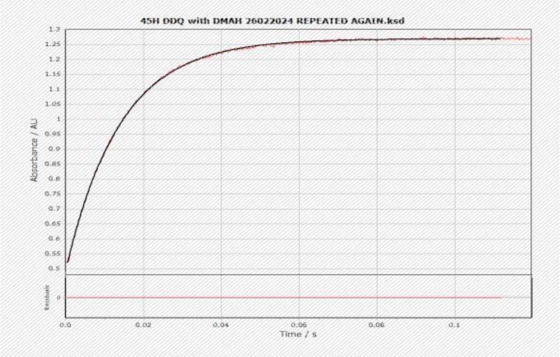

45°C H

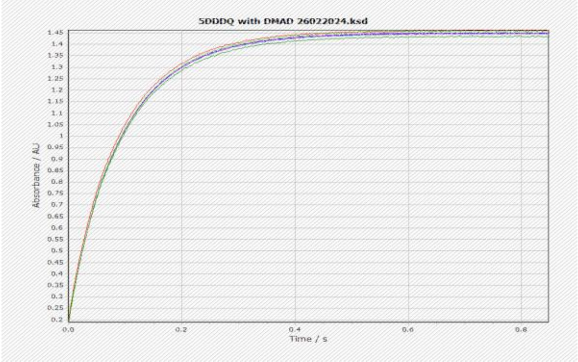

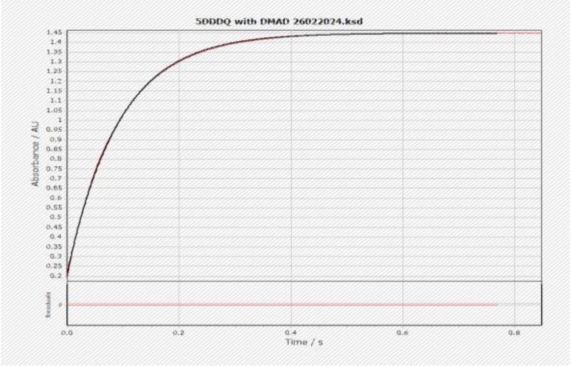

5°C D

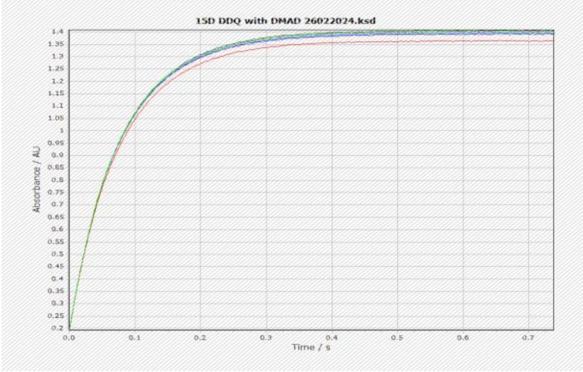

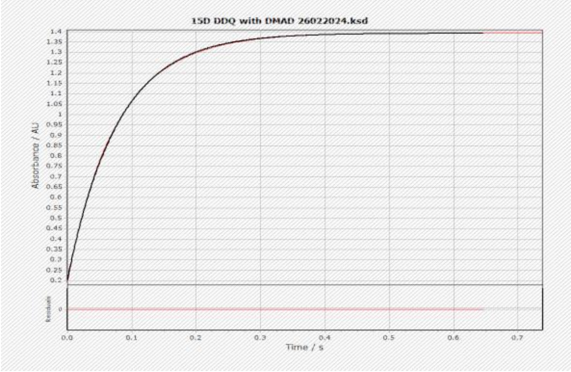

15°C D

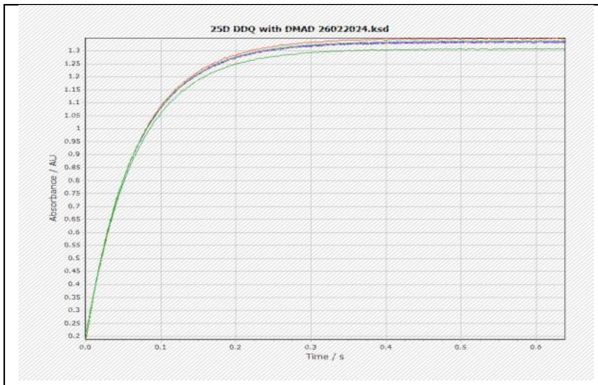

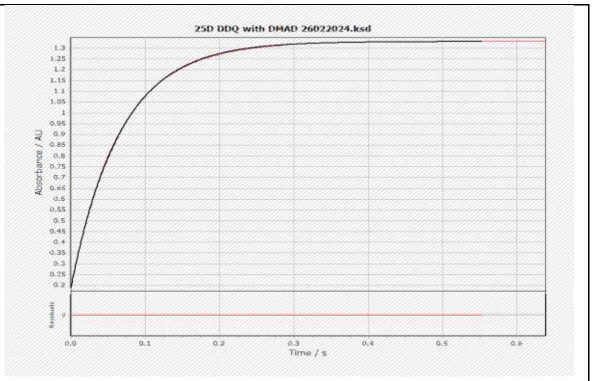

25°C D

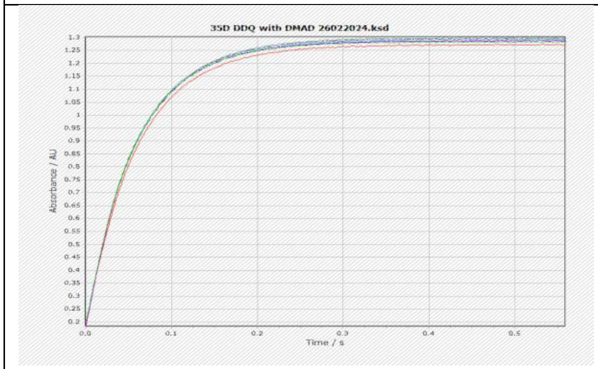

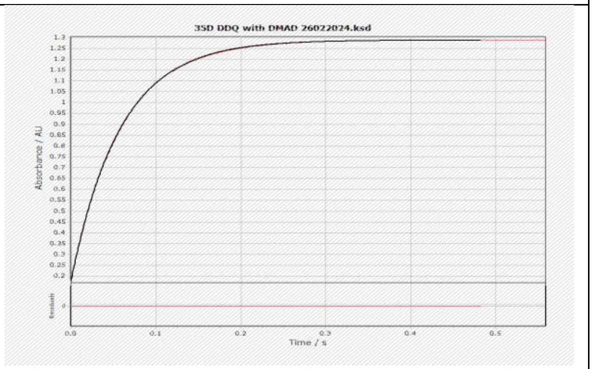

35°C D

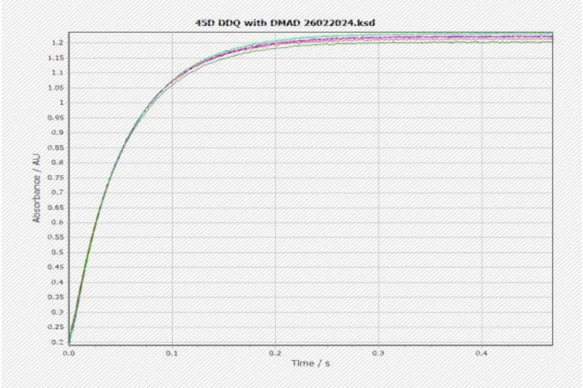

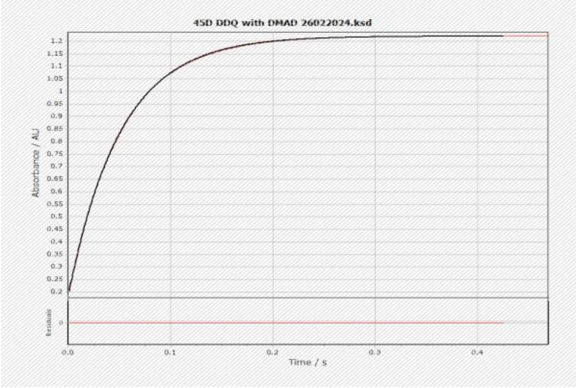

45°C D

Primary kinetic data for the rate constants in Table S3

Day 1 data (September 2, 2020)

	Pseudo-first-order rate constants k^{pfo} (s^{-1})									
**Temp (°C)**	**Trial H1**	**Trial H2**	**Trial H3**	**Trial H4**	**Trial H5**	**Trial H6**	**Average k_H^{pfo} (s^{-1})**	**Stdev**	**k_{2H} ($M^{-1}s^{-1}$)**	**Stdev [a]**
45	66.980	66.156	66.818	65.920	65.480	65.673	66.17162	0.610225	1.50E+05	1386.87476
35	62.799	62.229	62.117	61.370	60.412	61.358	61.71459	0.841945	1.40E+05	1913.51096
25	55.154	55.049	55.778	55.905	55.110	54.749	55.29122	0.451397	1.26E+05	1025.90280
15	49.573	49.204	48.687	49.055	49.165	48.686	49.0623	0.338851	1.12E+05	770.11627
5	42.236	41.973	41.377	41.136	41.291	41.537	41.59207	0.426052	9.45E+04	968.30048

Temp (°C)	Trial D1	Trial D2	Trial D3	Trial D4	Trial D5	Trial D6	Average k_D^{pfo} (s^{-1})	Stdev	k_{2D} ($M^{-1}s^{-1}$)	Stdev [a]
45	22.103	22.166	22.404	22.106	22.5263	22.431	22.28984	0.18588	3.35E+04	279.38274
35	18.251	18.623	19.029	18.587	18.8365	18.832	18.69354	0.269961	2.81E+04	405.75925
25	15.262	15.079	15.293	15.296	15.4373	15.492	15.31020	0.145403	2.30E+04	218.54426
15	12.360	12.252	12.195	12.134	12.2420	12.370	12.25925	0.092216	1.84E+04	138.60312
5	10.105	9.797	9.8581	9.826	9.79861	9.8402	9.87111	0.11706	1.48E+04	175.94440

[a] = (Stdev(for k^{pfo})/k^{pfo})*k_{2H}

<table>
<tr><td>Six kinetic runs together with the averaged data</td><td>First-order kinetic fit of the averaged data as an example</td></tr>
<tr><td>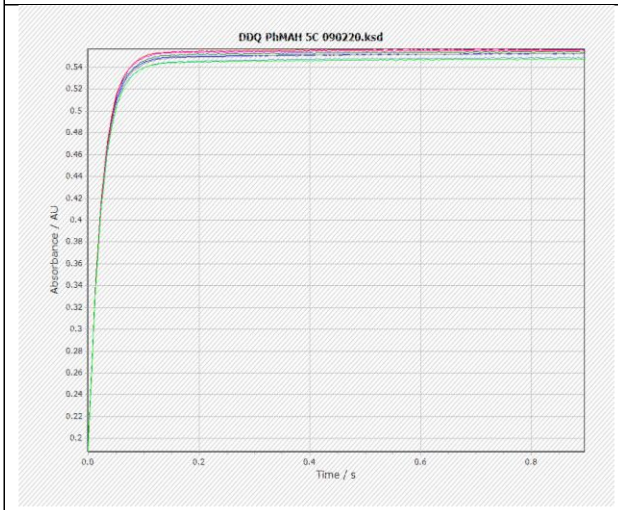
</td><td>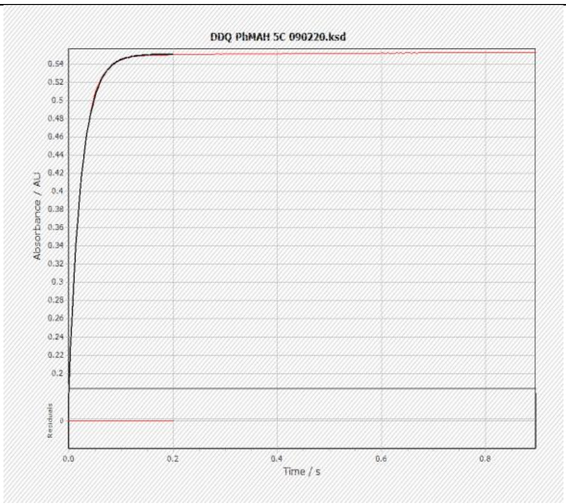
</td></tr>
<tr><td colspan="2">5℃ H</td></tr>
<tr><td>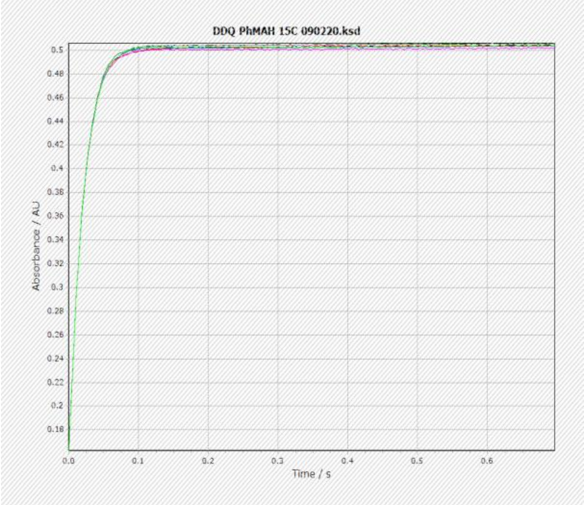
</td><td>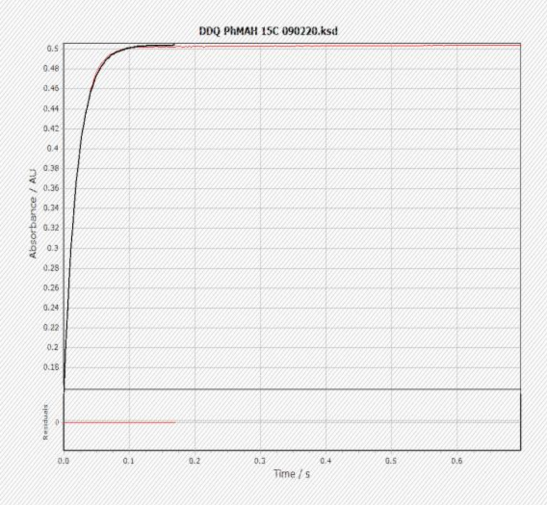
</td></tr>
<tr><td colspan="2">15℃ H</td></tr>
</table>

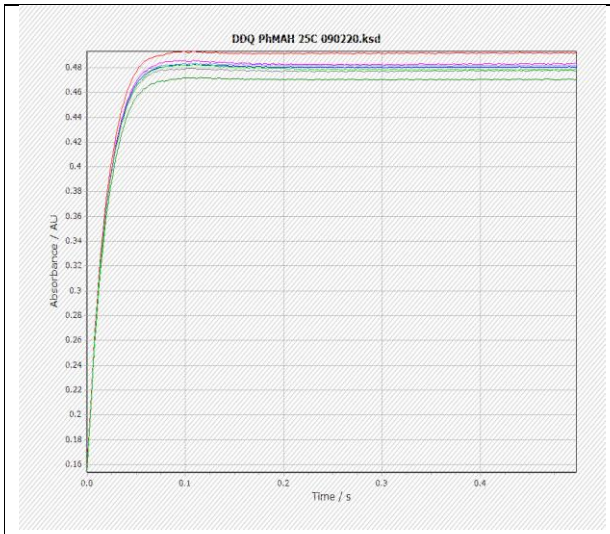

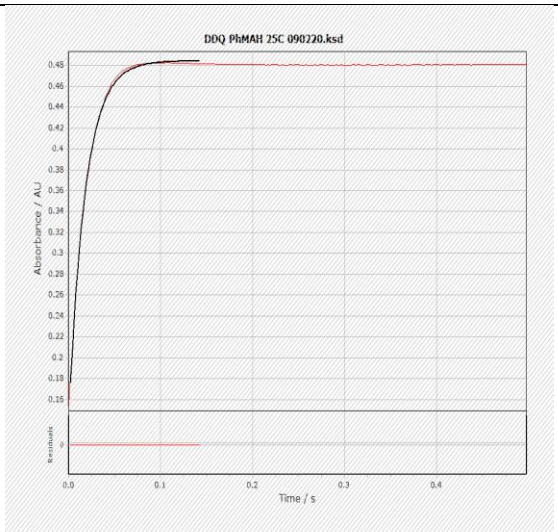

25°C H

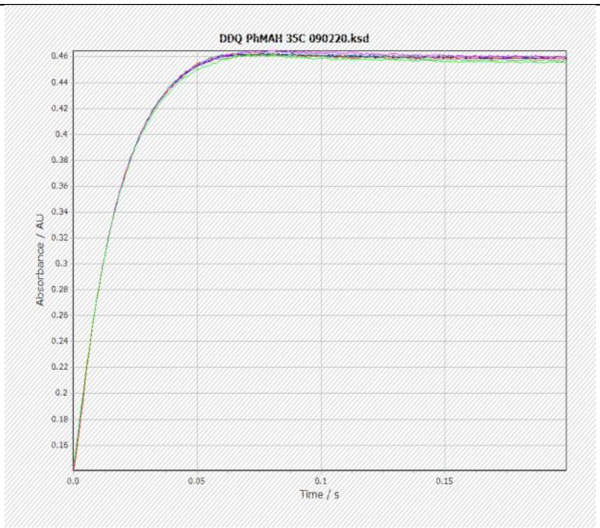

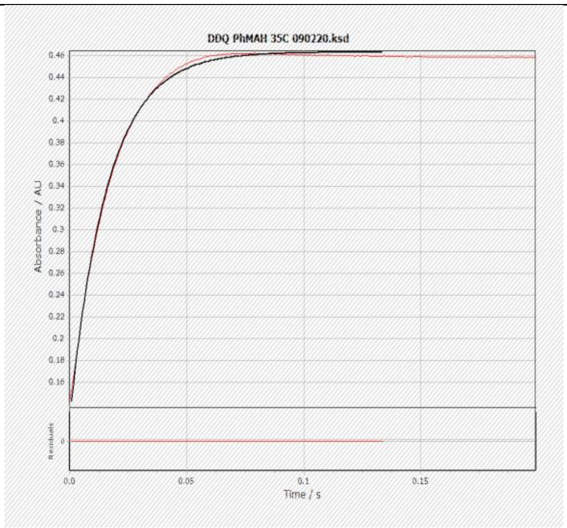

35°C H

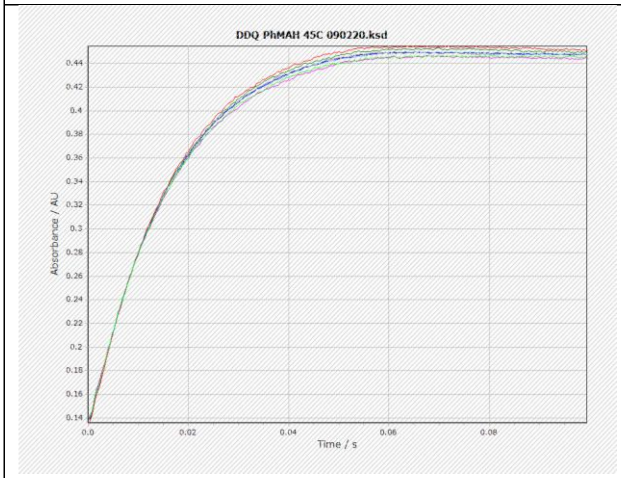

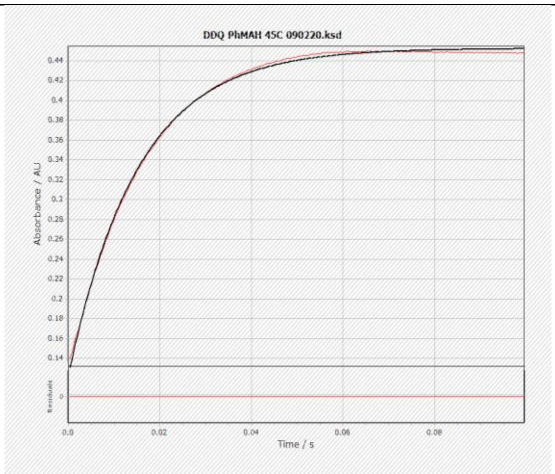

45°C H

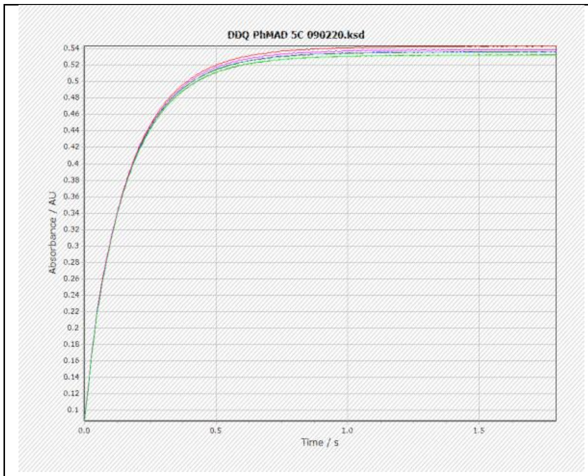

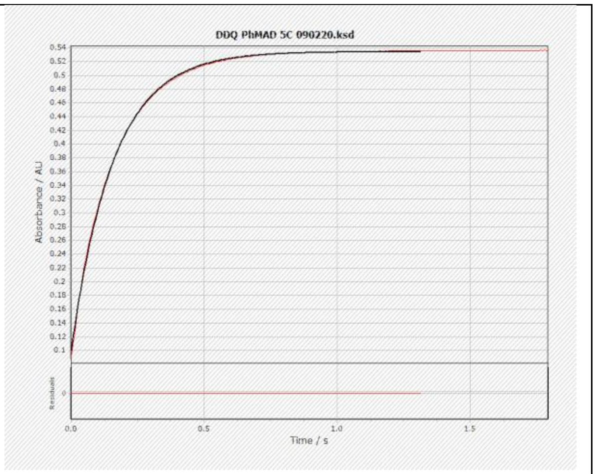

5°C D

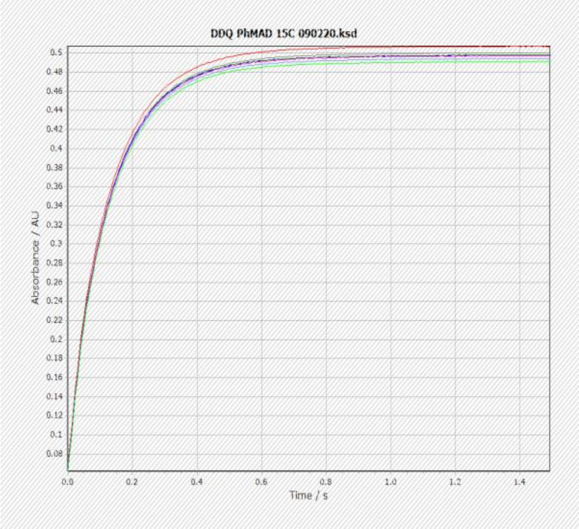

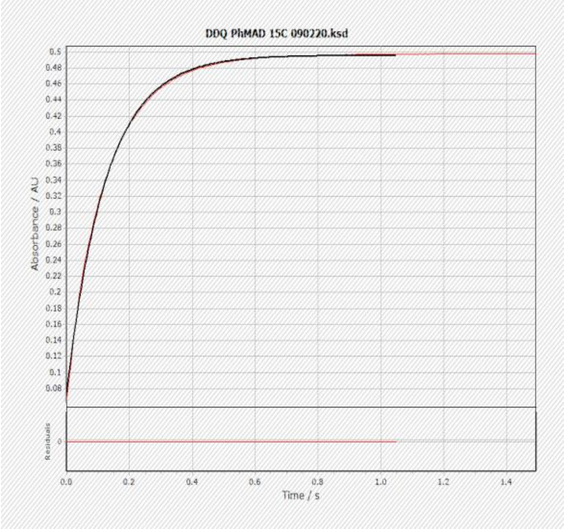

15°C D

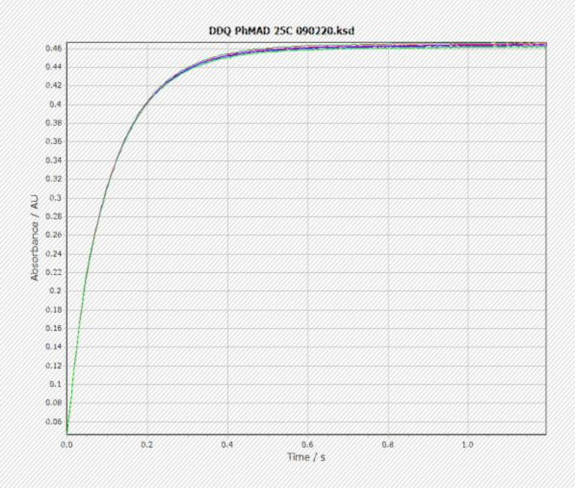

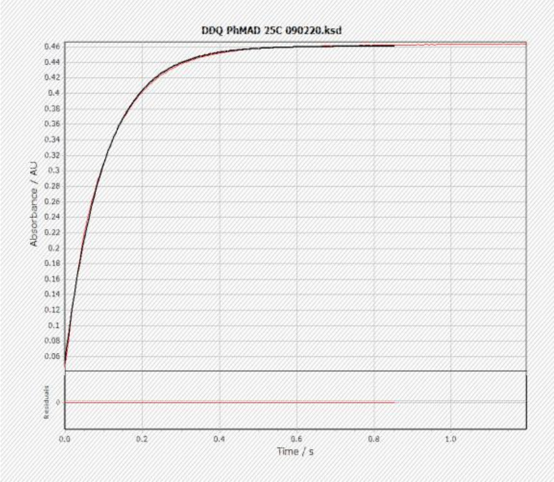

25°C D

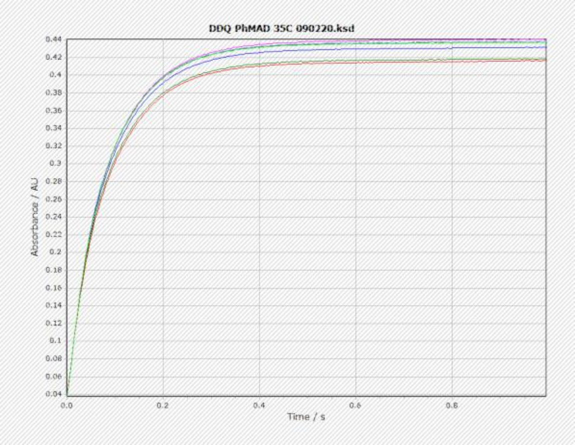

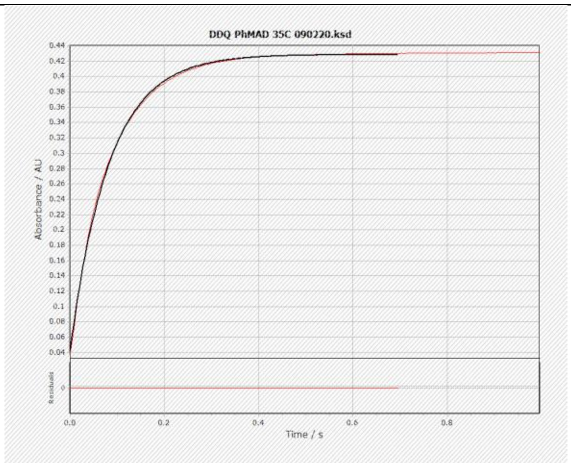

35°C D

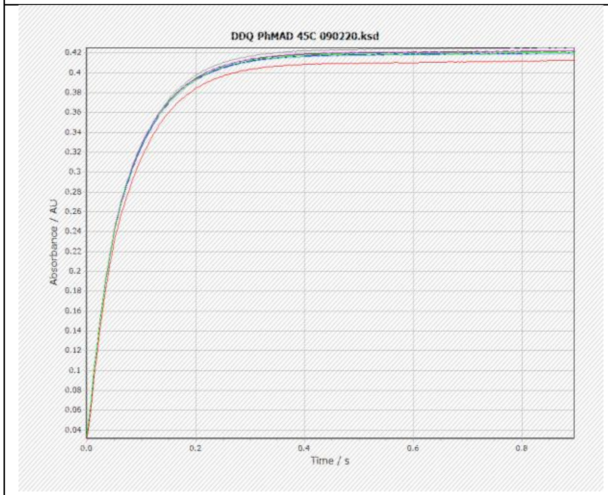

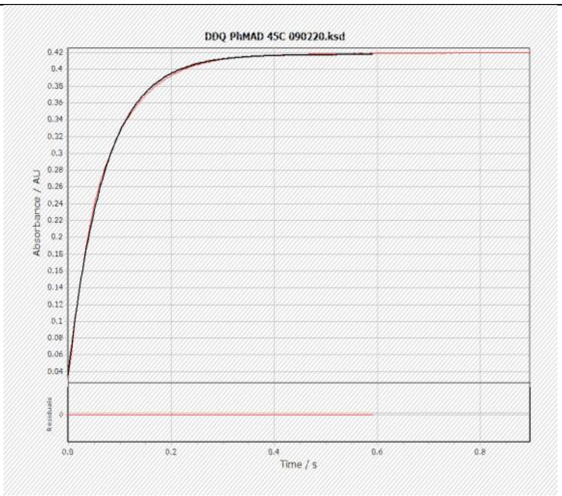

45°C D

Day 2 data (September 3, 2020)

	Pseudo-first-order rate constants									
	k^{pfo} (s^{-1})									
**Temp (°C)**	**Trial H1**	**Trial H2**	**Trial H3**	**Trial H4**	**Trial H5**	**Trial H6**	**Average k_H^{pfo} (s^{-1})**	**Stdev**	**k_{2H} ($M^{-1}s^{-1}$)**	**Stdev [a]**
45	99.131	95.867	95.694	95.471	93.668	92.540	95.39541	2.254137	1.45E+05	3415.35842
35	89.950	88.344	88.239	86.401	85.8907	85.639	87.41111	1.701522	1.32E+05	2578.06361
25	80.160	79.137	78.837	79.479	79.5285	80.089	79.53895	0.518844	1.21E+05	786.12692
15	78.550	70.766	71.333	69.925	69.9115	70.926	71.90254	3.30566	1.09E+05	5008.57503
5	61.401	61.580	61.617	62.255	62.6776	62.819	62.05867	0.609371	9.40E+04	923.28972
**Temp (°C)**	**Trial D1**	**Trial D2**	**Trial D3**	**Trial D4**	**Trial D5**	**Trial D6**	**Average k_D^{pfo} (s^{-1})**	**Stdev**	**k_{2D} ($M^{-1}s^{-1}$)**	**Stdev [a]**
45	13.917	14.311	14.101	14.347	14.247	14.297	14.20366	0.164452	3.16E+04	365.95300
35	11.934	12.115	12.063	12.227	12.002	12.248	12.09862	0.123739	2.69E+04	275.35424
25	9.727	9.783	9.826	9.915	9.86815	9.914	9.83926	0.074855	2.19E+04	166.57351
15	7.942	8.024	7.947	8.012	7.97449	8.036	7.98964	0.040341	1.78E+04	89.77000
5	6.235	6.343	6.357	6.364	6.35859	6.443	6.35062	0.066646	1.41E+04	148.30718

[a] [DDQ] = 0.044 mM, [PhMAH] = 0.66 mM; [b] = (Stdev(for k^{pfo})/k^{pfo})*k_{2H}

Six kinetic runs together with the averaged data	First-order kinetic fit of the averaged data as an example
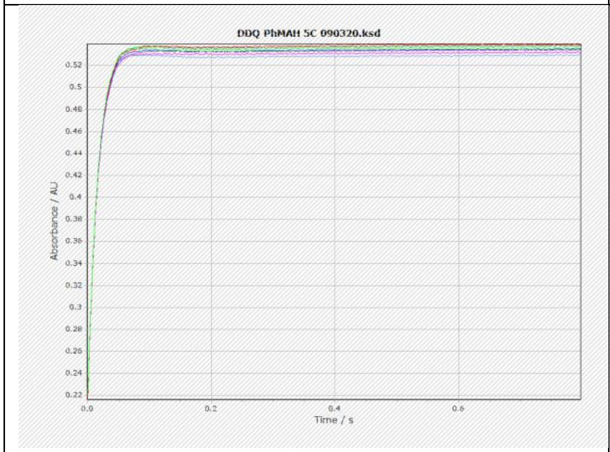	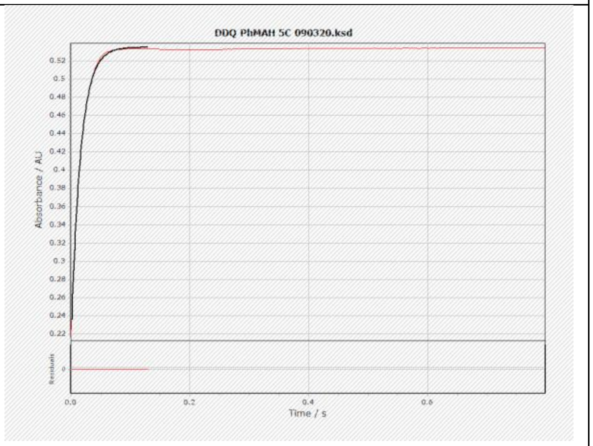
5°C H	
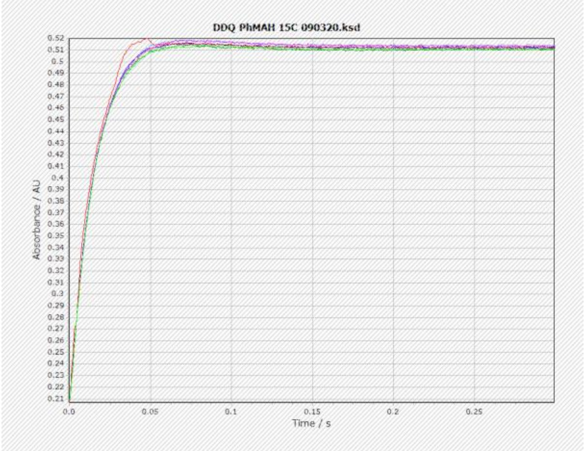	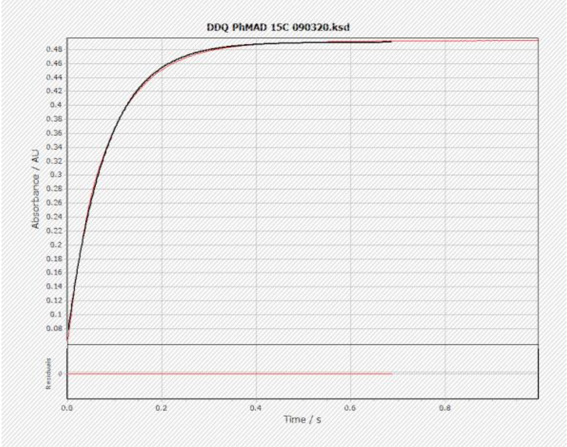
15°C H	
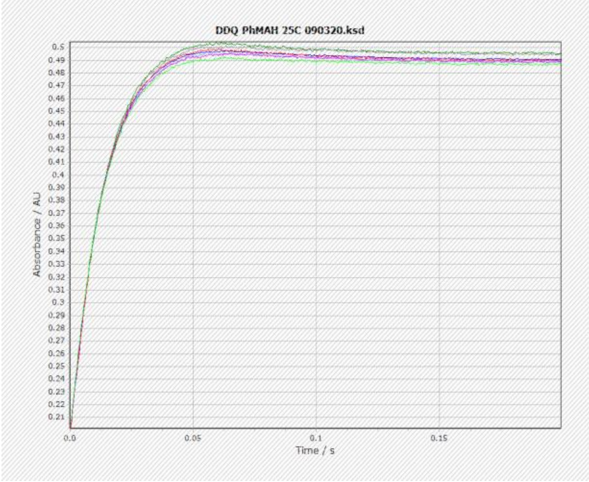	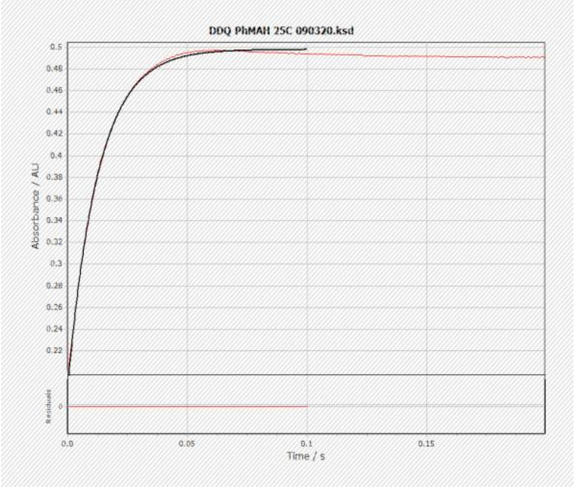
25°C H	

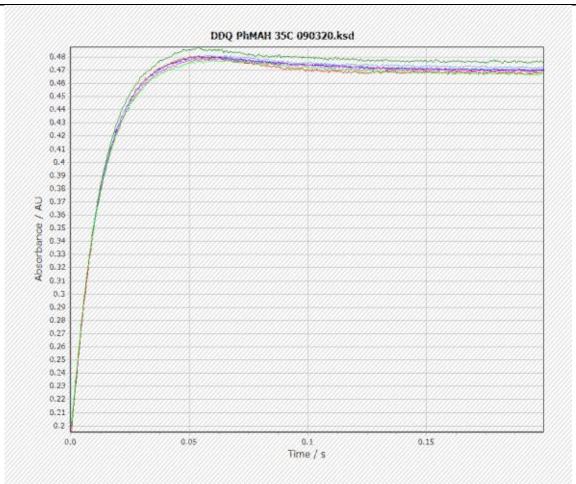
DDQ PhMAH 35C 090320.ksd
Absorbance / AU
Time / s

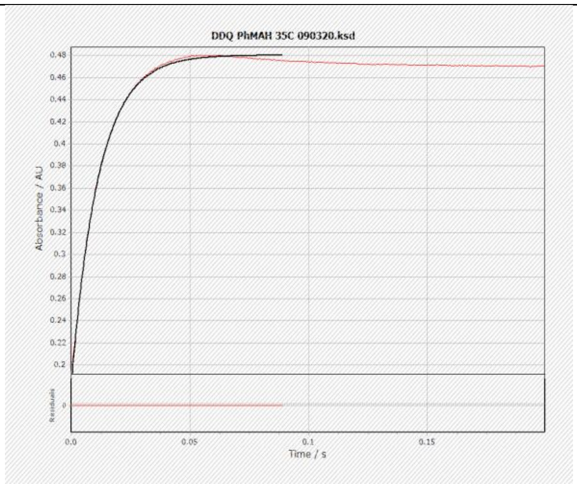
DDQ PhMAH 35C 090320.ksd
Absorbance / AU
Residuals
Time / s

35℃ H

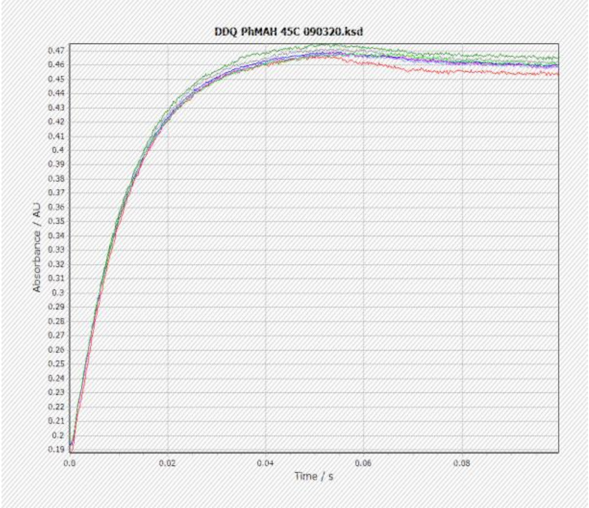
DDQ PhMAH 45C 090320.ksd
Absorbance / AU
Time / s

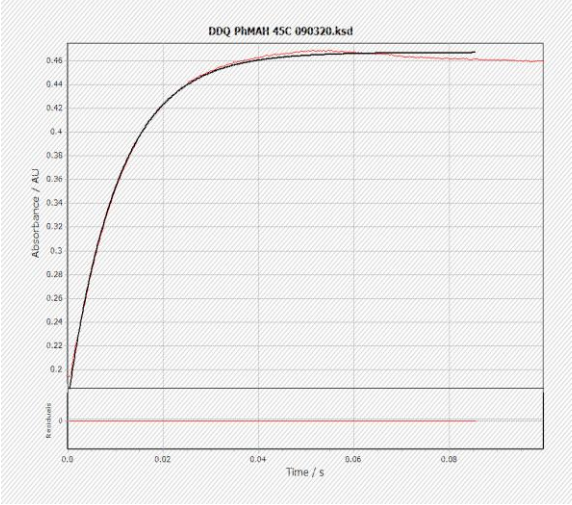
DDQ PhMAH 45C 090320.ksd
Absorbance / AU
Residuals
Time / s

45℃ H

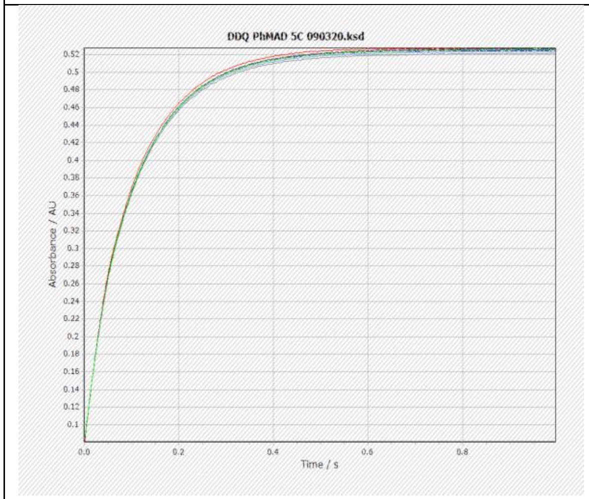
DDQ PhMAD 5C 090320.ksd
Absorbance / AU
Time / s

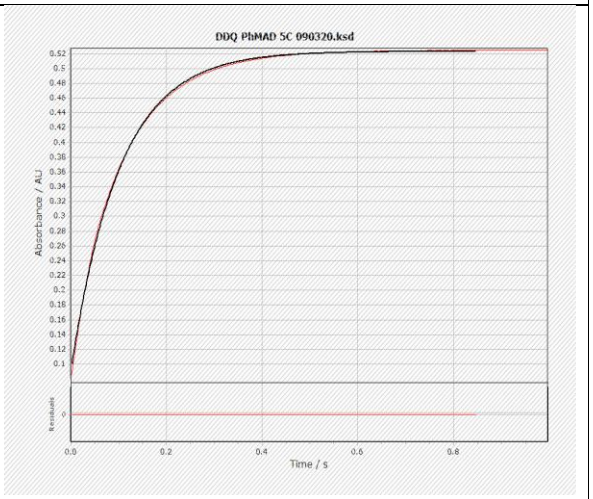
DDQ PhMAD 5C 090320.ksd
Absorbance / AU
Residuals
Time / s

5℃ D

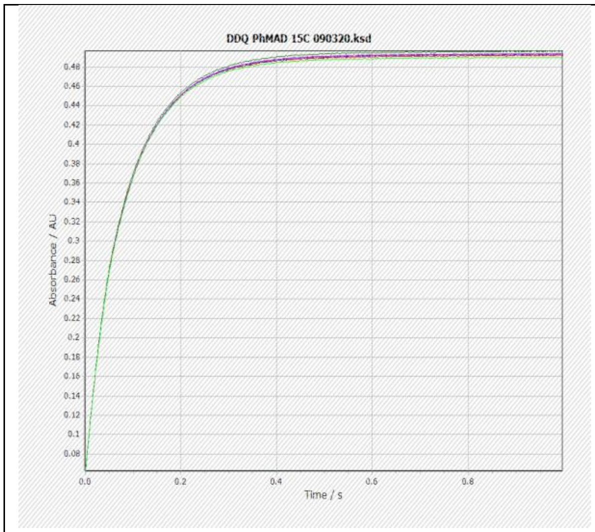

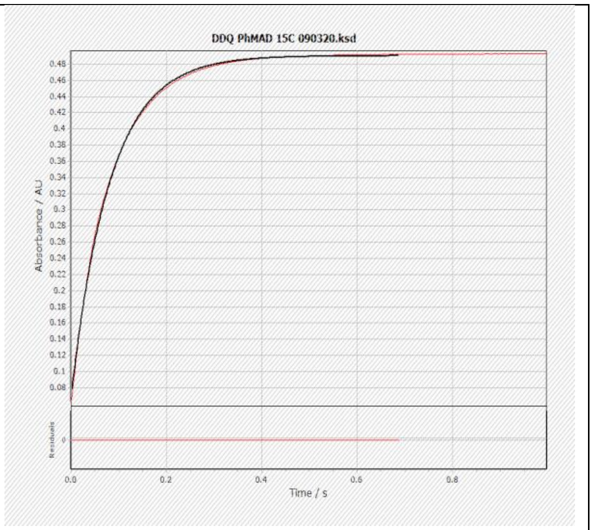

15°C D

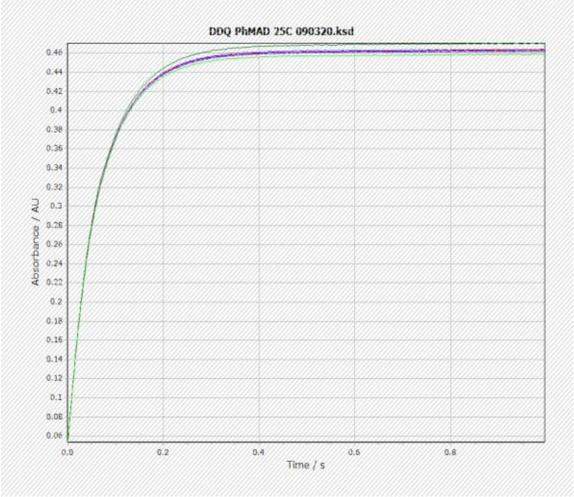

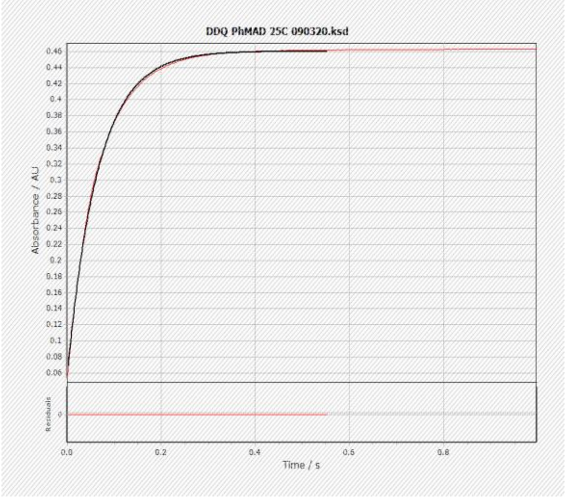

25°C D

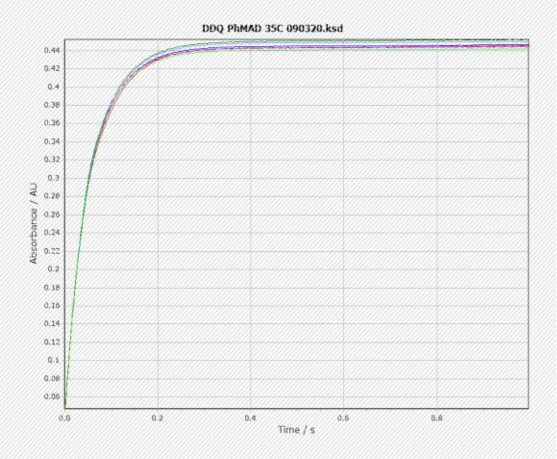

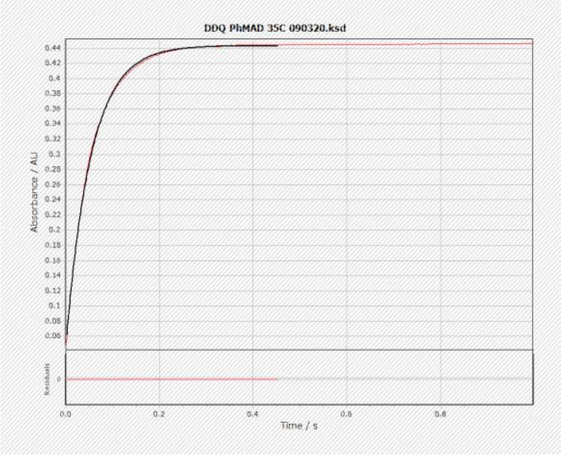

35°C D

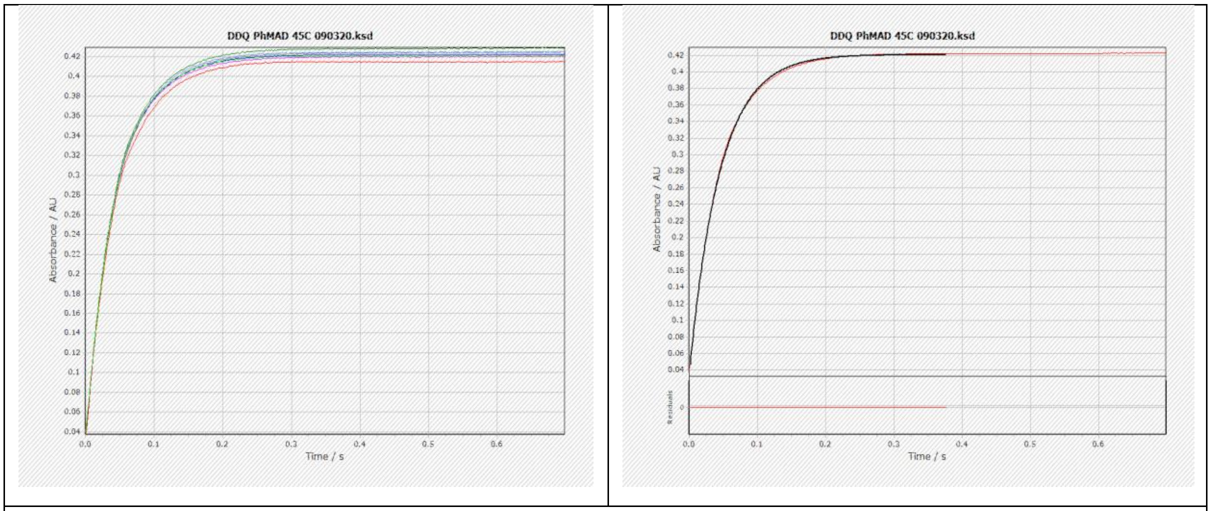

45°C D

Day 3 data (September 8, 2020)

Pseudo-first-order rate constants

Temp (°C)	Trial H1	Trial H2	Trial H3	Trial H4	Trial H5	Trial H6	Average k_H^{pfo} (s^{-1})	Stdev	k_{2H} ($M^{-1}s^{-1}$)	Stdev [a]
	k^{pfo} (s^{-1})									
45	69.873	67.716	66.864	66.574	65.538	64.995	66.92727	1.737427	1.52E+05	3948.69857
35	63.014	61.730	61.368	62.019	61.018	60.890	61.67347	0.781808	1.40E+05	1776.83632
25	55.251	55.748	55.188	54.702	54.611	53.640	54.85716	0.724411	1.25E+05	1646.38853
15	45.490	48.605	48.015	48.329	48.229	48.134	47.80075	1.14943	1.09E+05	2612.34155
5	41.751	41.298	41.817	41.811	42.015	42.042	41.78944	0.26805	9.50E+04	609.20418

Temp (°C)	Trial D1	Trial D2	Trial D3	Trial D4	Trial D5	Trial D6	Average k_D^{pfo} (s^{-1})	Stdev	k_{2D} ($M^{-1}s^{-1}$)	Stdev [a]
45	13.968	14.051	14.065	14.163	14.302	14.381	14.15557	0.159331	3.35E+04	376.94766
35	12.296	12.289	12.231	12.375	12.306	12.447	12.32445	0.075993	2.92E+04	179.78557
25	9.654	9.8064	9.681	9.7950	9.8726	9.868	9.77986	0.092469	2.31E+04	218.76370
15	7.671	7.7191	7.8231	7.8563	7.8113	7.8796	7.79359	0.081136	1.84E+04	191.95336
5	6.223	6.1733	6.1450	6.2420	6.3329	6.2987	6.23588	0.071749	1.48E+04	169.74446

[a] = (Stdev(for k^{pfo})/k^{pfo})*k_{2H}

<table>
<tr><th>Six kinetic runs together with the averaged data</th><th>First-order kinetic fit of the averaged data as an example</th></tr>
<tr><td>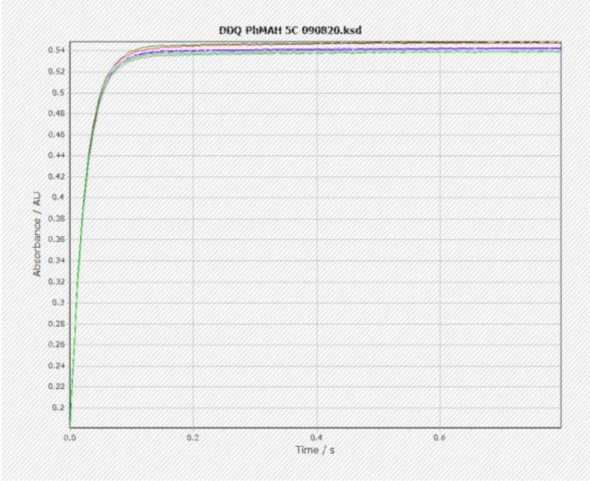
</td><td>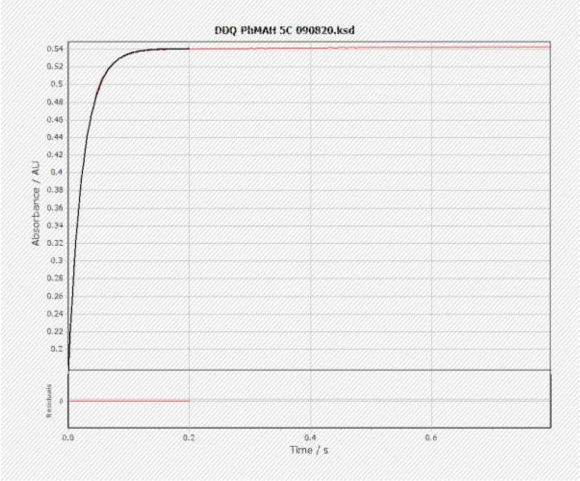
</td></tr>
<tr><td colspan="2">5℃ H</td></tr>
<tr><td>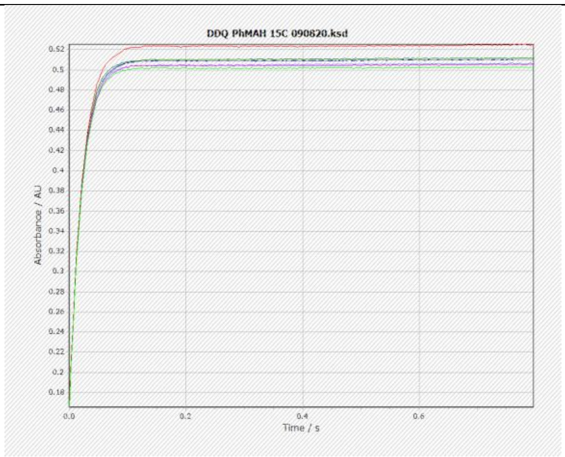
</td><td>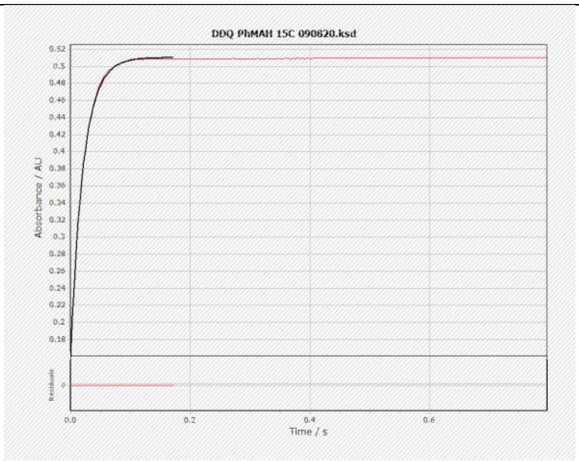
</td></tr>
<tr><td colspan="2">15℃ H</td></tr>
<tr><td>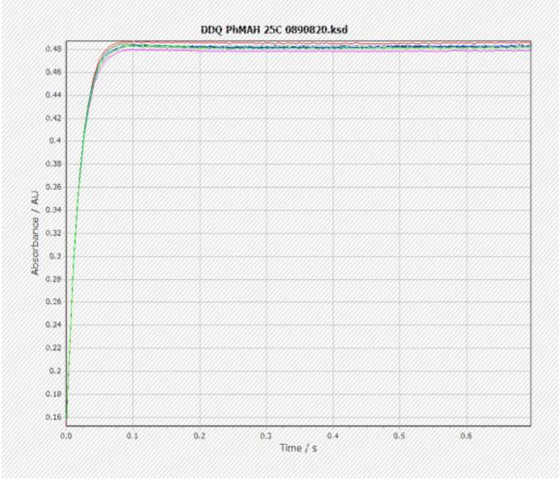
</td><td>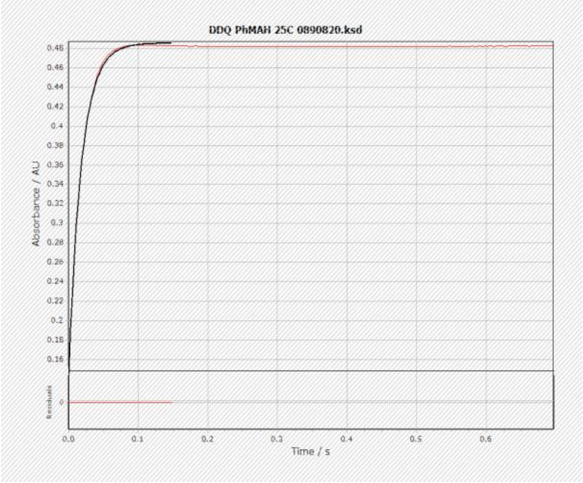
</td></tr>
<tr><td colspan="2">25℃ H</td></tr>
</table>

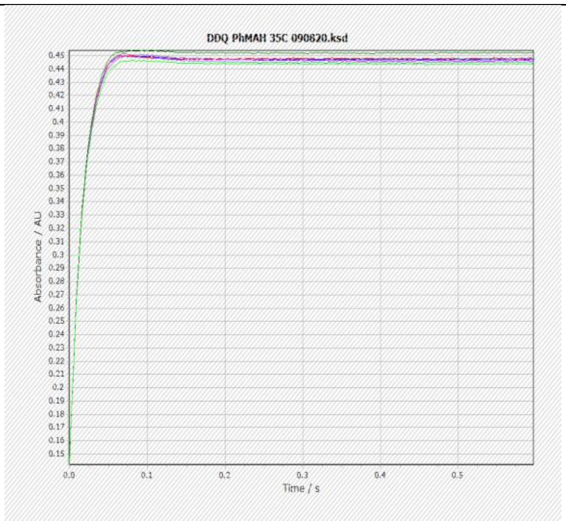
DDQ PhMAH 35C 090820.ksd
Absorbance / AU
Time / s

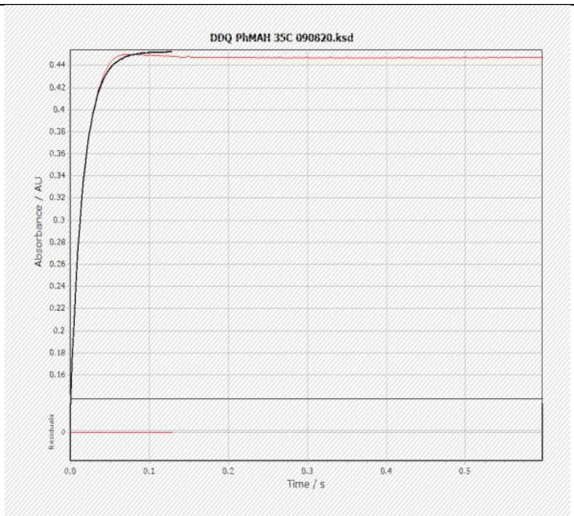
DDQ PhMAH 35C 090820.ksd
Absorbance / AU
Residuals
Time / s

35℃ H

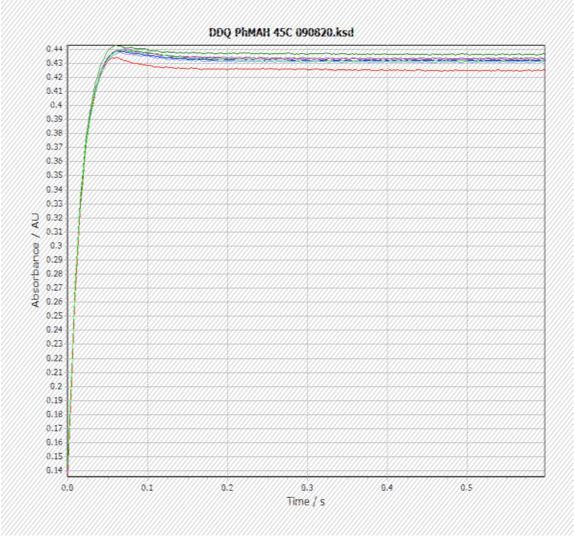
DDQ PhMAH 45C 090820.ksd
Absorbance / AU
Time / s

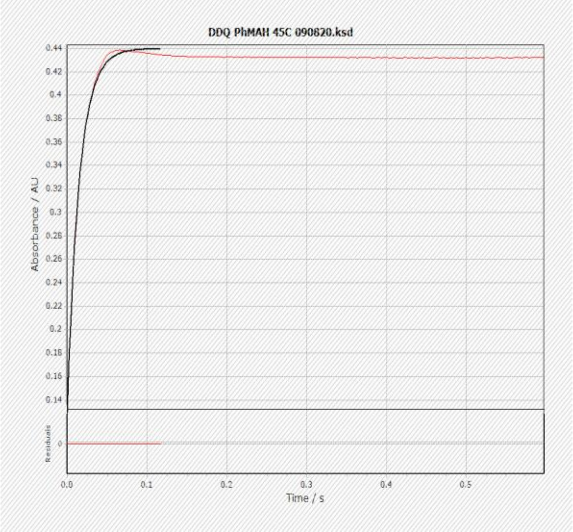
DDQ PhMAH 45C 090820.ksd
Absorbance / AU
Residuals
Time / s

45℃ H

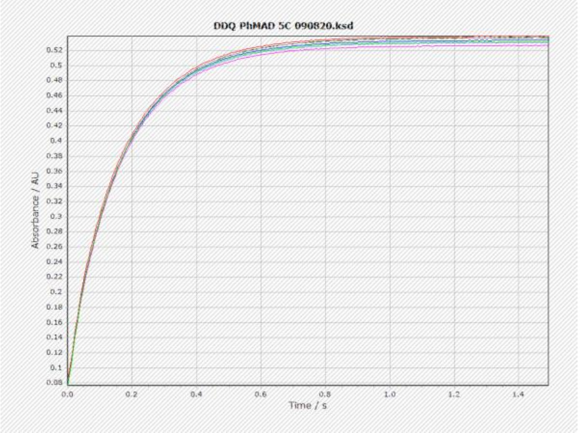
DDQ PhMAD 5C 090820.ksd
Absorbance / AU
Time / s

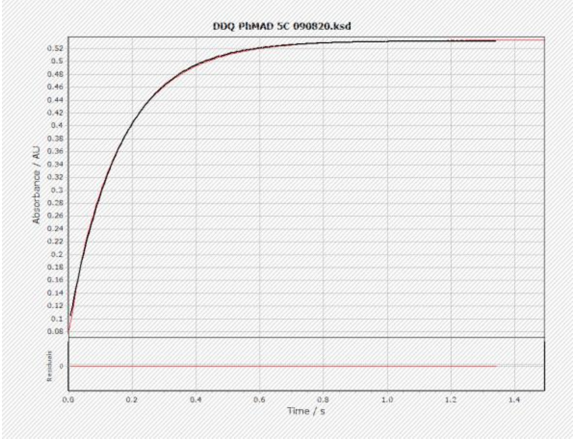
DDQ PhMAD 5C 090820.ksd
Absorbance / AU
Residuals
Time / s

5℃ D

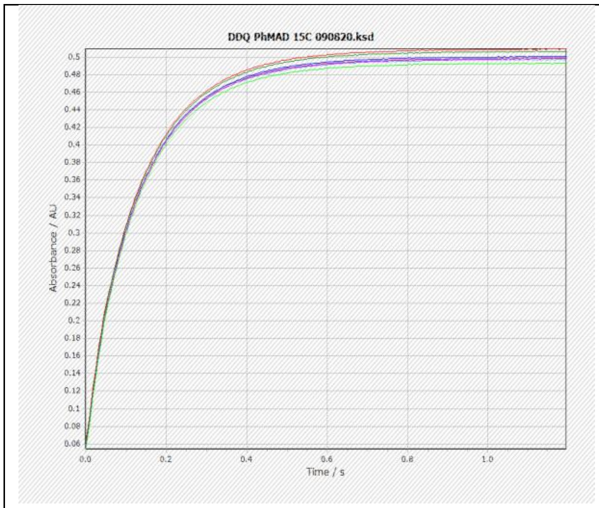

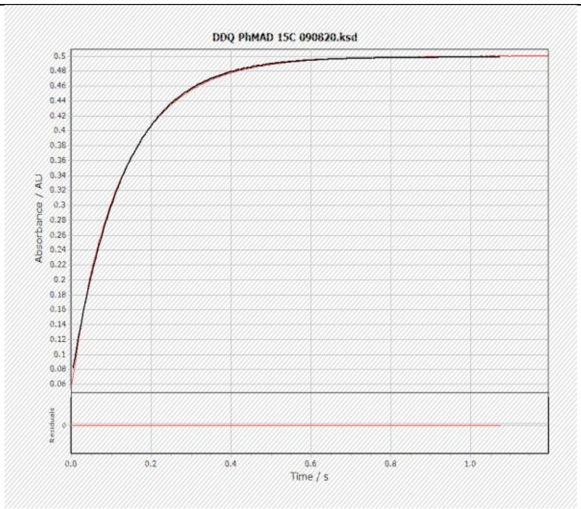

15°C D

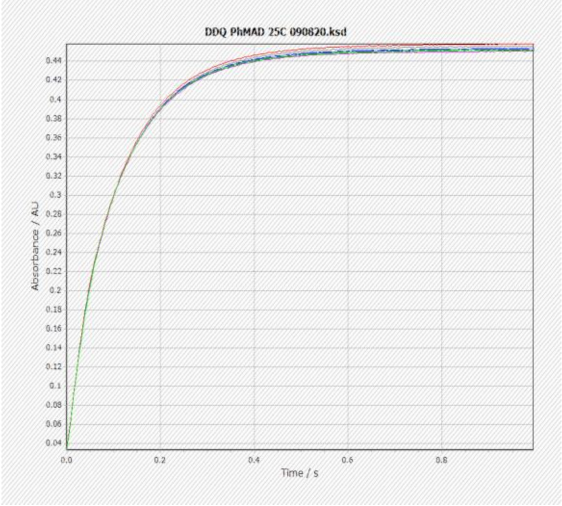

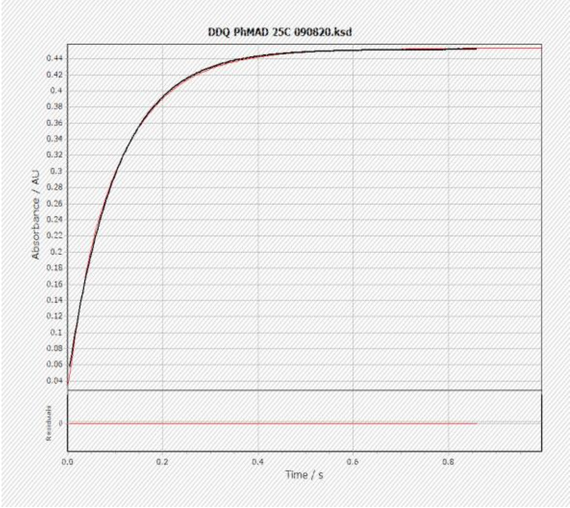

25°C D

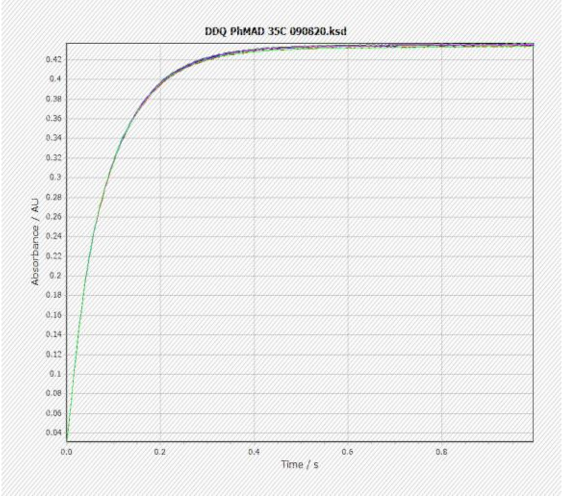

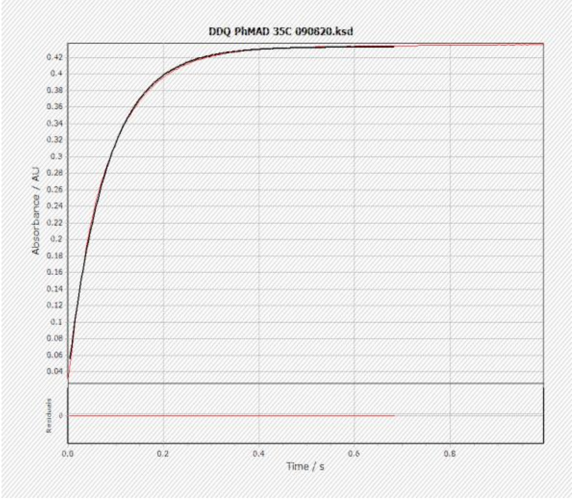

35°C D

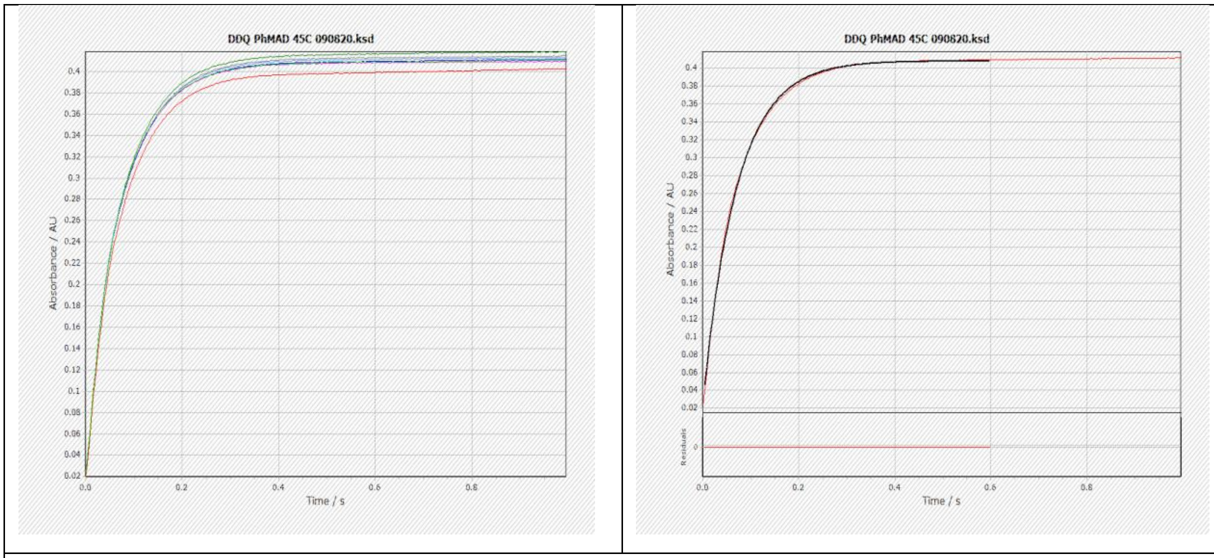

45°C D

Primary kinetic data for the rate constants in Table S4

Day 1 data
(August 31, 2025)

					Pseudo-first-order rate constants k^{pfo} (s^{-1})					
**Temp (°C)**	**Trial H1**	**Trial H2**	**Trial H3**	**Trial H4**	**Trial H5**	**Trial H6**	**Average k_H^{pfo} (s^{-1})**	**Stdev**	**k_{2H} ($M^{-1}s^{-1}$)**	**Stdev [a]**
45	30.56823	27.53090	29.13659	27.82931	27.26775	27.14326	28.24601	1.34E+00	5.65E+03	2.69E+02
35	23.09768	20.69136	21.02512	20.68573	20.08723	20.57055	21.02628	1.06E+00	4.21E+03	2.12E+02
25	14.35586	14.48742	14.57297	14.64697	14.64470	14.43926	14.52453	1.17E-01	2.90E+03	2.35E+01
15	8.78340	9.50375	9.58643	9.50494	9.80138	9.76791	9.49130	3.70E-01	1.90E+03	7.39E+01
5	5.41697	5.81244	6.10575	6.07709	6.10033	6.04982	5.92707	2.73E-01	1.19E+03	5.46E+01
**Temp (°C)**	**Trial D1**	**Trial D2**	**Trial D3**	**Trial D4**	**Trial D5**	**Trial D6**	**Average k_D^{pfo} (s-1)**	**Stdev**	**k_{2D} (M-1s-1)**	**Stdev [a]**
45	2.11398	2.43105	2.35967	2.43527	2.43231	2.37472	2.3578	1.24E-01	4.72E+02	2.48E+01
35	1.45233	1.45922	1.43203	1.43154	1.41936	1.41583	1.4351	1.74E-02	2.87E+02	3.49E+00
25	0.84012	0.85394	0.86130	0.86131	0.85974	0.86569	0.8570	9.10E-03	1.71E+02	1.82E+00
15	0.47986	0.51040	0.51843	0.51161	0.50711	0.50819	0.5059	1.34E-02	1.01E+02	2.68E+00
5	0.25438	0.2573	0.26001	0.26055	0.25974	0.26056	0.2588	2.46E-03	5.18E+01	4.92E-01

[a] = (Stdev(for k^{pfo})/k^{pfo})*k_2

Six kinetic trials and the mean fit	First-order kinetic fitted average of six trials
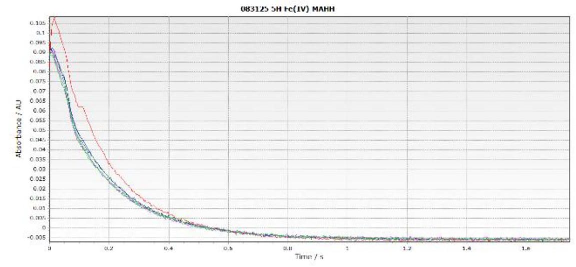	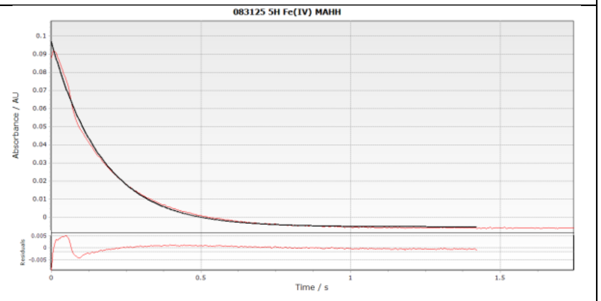
5°C H	
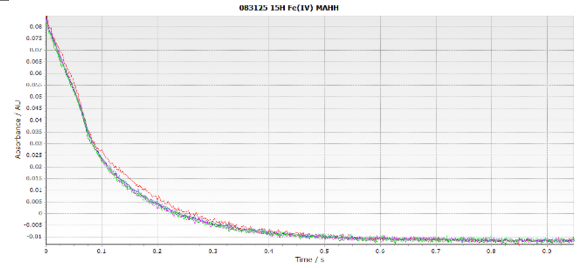	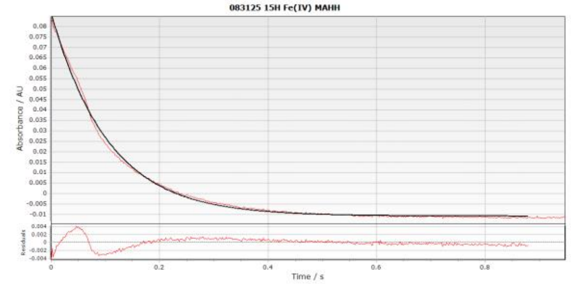
15°C H	
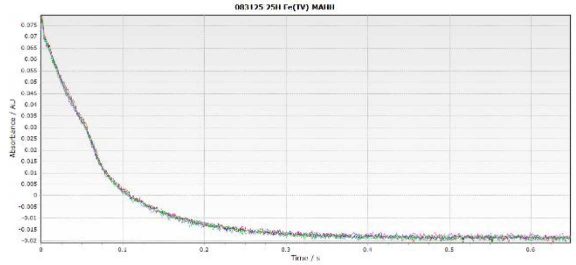	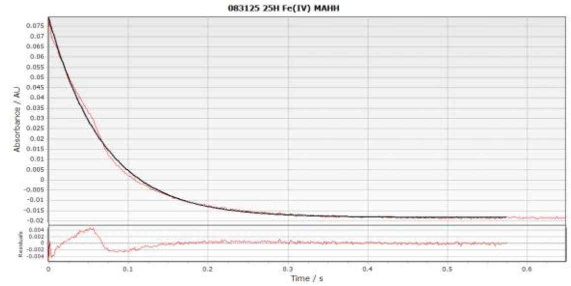
25°C H	
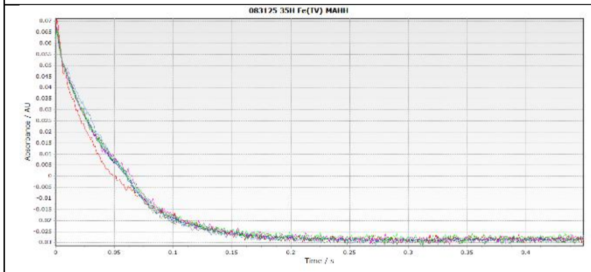	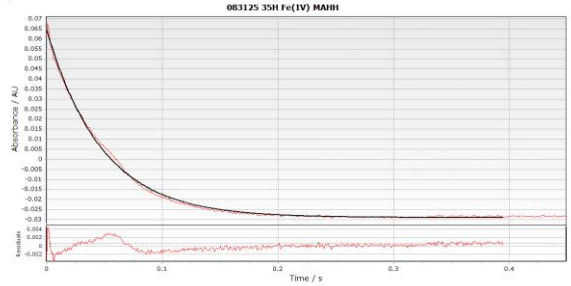
35°C H	

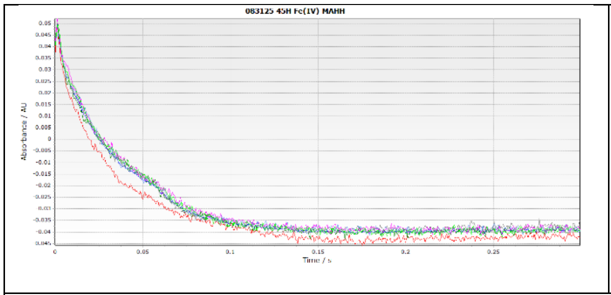

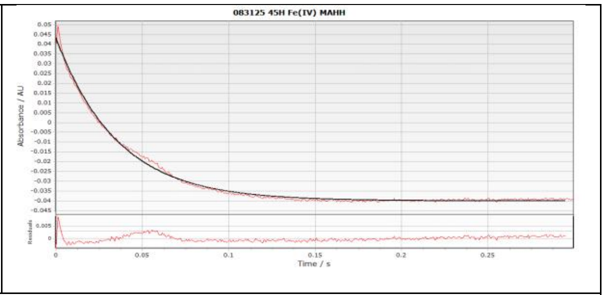

45°C H

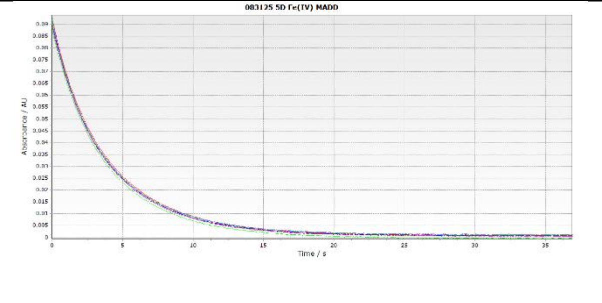

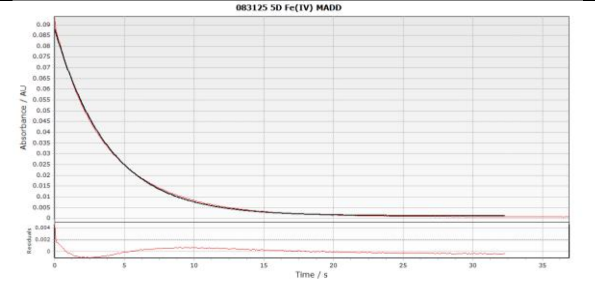

5°C D

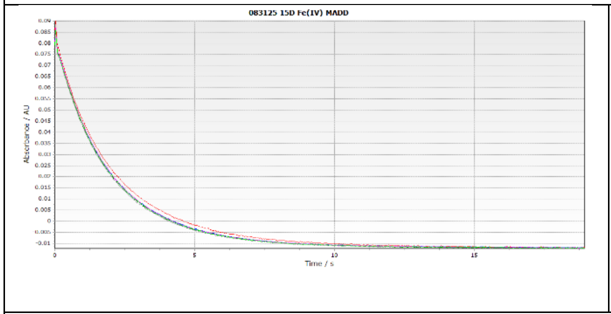

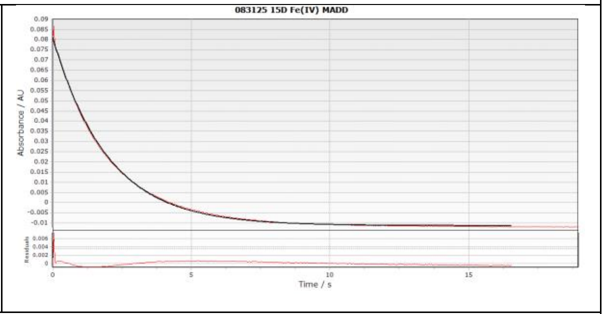

15°C D

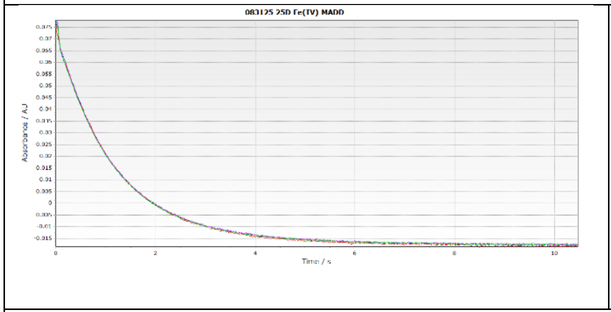

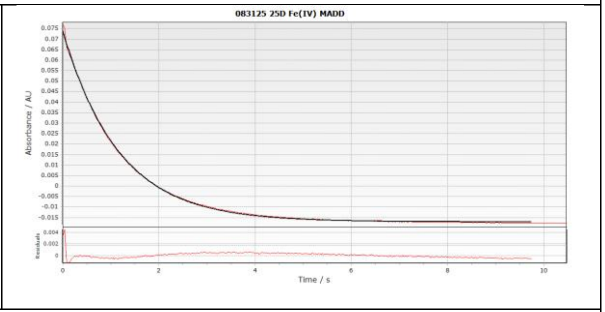

25°C D

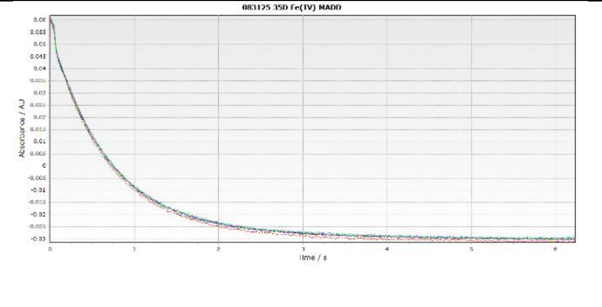

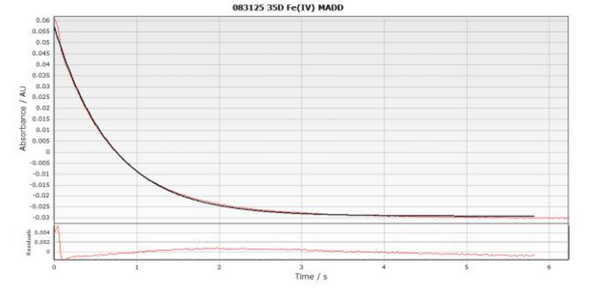

35°C D

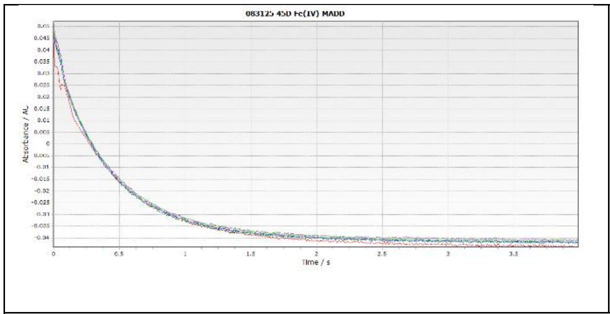

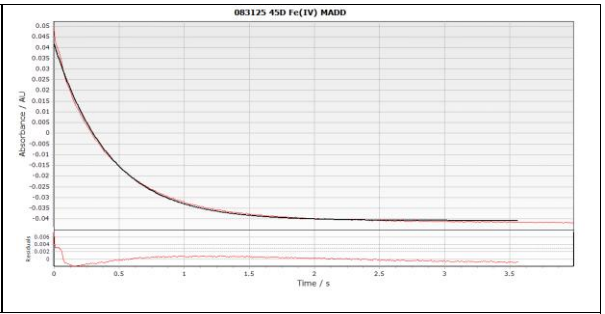

45℃ D

Day 2 data (September 16, 2025)

Pseudo-first-order rate constants

k^{pfo} (s^{-1})

Temp (°C)	Trial H1	Trial H2	Trial H3	Trial H4	Trial H5	Trial H6	Average k_H^{pfo} (s^{-1})	Stdev	k_{2H} ($M^{-1}s^{-1}$)	Stdev [a]
45	26.21657	25.77006	27.39667	27.77564	26.21870	27.23720	26.76914	8.04E-01	5.35E+03	1.61E+02
35	19.57096	19.68326	19.36136	19.43391	19.18608	18.68900	19.32076	3.54E-01	3.86E+03	7.08E+01
25	13.62063	13.59948	13.57760	13.36173	13.23339	13.18709	13.42999	1.95E-01	2.69E+03	3.89E+01
15	8.93117	9.34037	9.31437	9.25151	9.15437	8.98444	9.16271	1.72E-01	1.83E+03	3.44E+01
5	5.53168	5.54530	5.65307	5.71170	5.83624	5.68759	5.66093	1.13E-01	1.13E+03	2.27E+01

Temp (°C)	Trial D1	Trial D2	Trial D3	Trial D4	Trial D5	Trial D6	Average k_D^{pfo} (s-1)	Stdev	k_{2D} (M-1s-1)	Stdev [a]
45	2.47171	2.47357	2.45903	2.50271	2.46977	2.46282	2.4733	1.54E-02	4.95E+02	3.09E+00
35	1.55100	1.54198	1.50847	1.47738	1.47278	1.48818	1.5066	3.34E-02	3.01E+02	6.67E+00
25	0.81255	0.83154	0.82937	0.82765	0.82665	0.82937	0.8262	6.89E-03	1.65E+02	1.38E+00
15	0.47925	0.48810	0.49455	0.49124	0.48666	0.49100	0.4885	5.28E-03	9.77E+01	1.06E+00
5	0.24777	0.25186	0.25079	0.25006	0.25107	0.25499	0.2511	2.37E-03	5.02E+01	4.73E-01

[a] = (Stdev(for k^{pfo})/k^{pfo})*k_2

Six kinetic trials and the mean fit

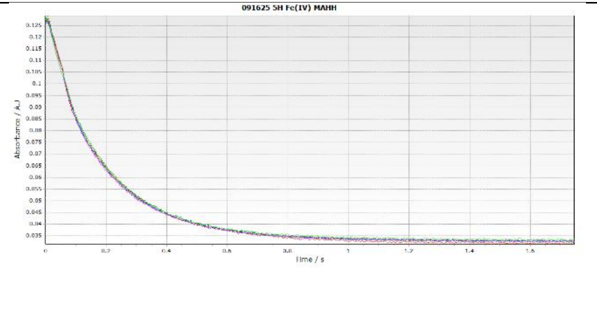

First-order kinetic fitted average of six trials

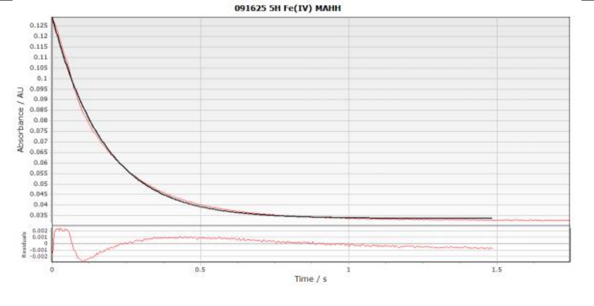

5℃ H

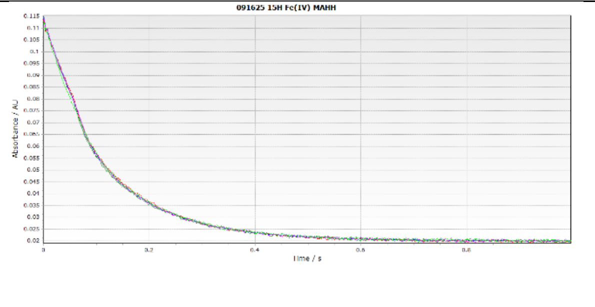

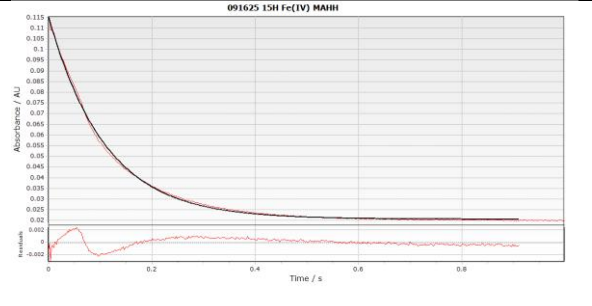

15°C H

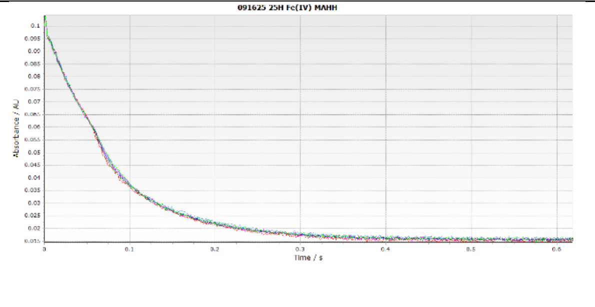

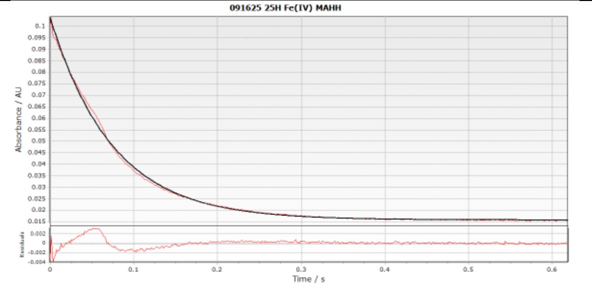

25°C H

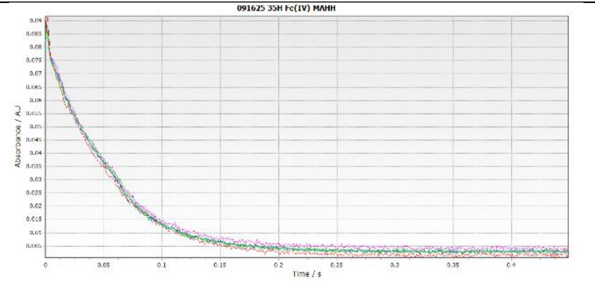

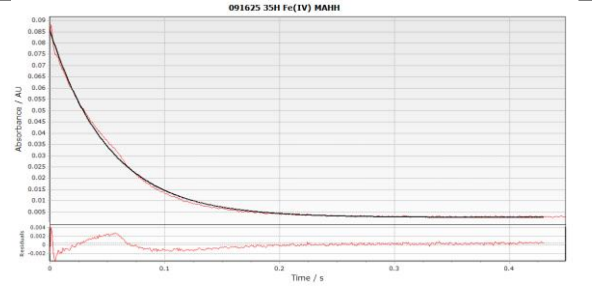

35°C H

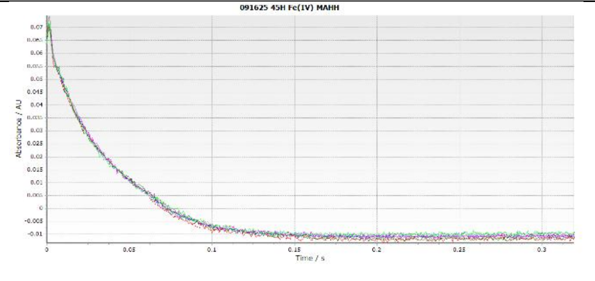

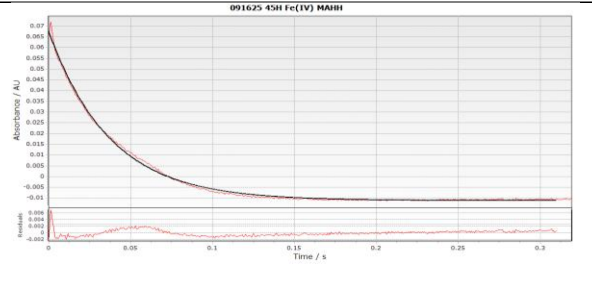

45°C H

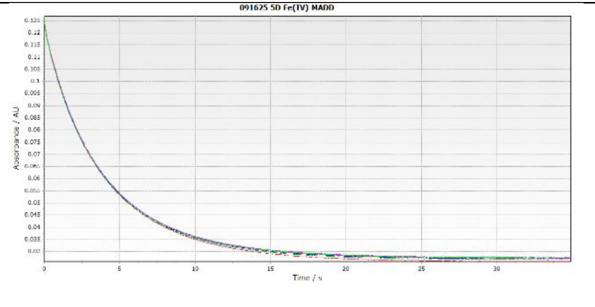

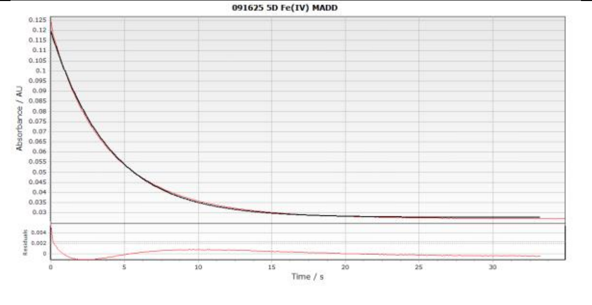

5°C D

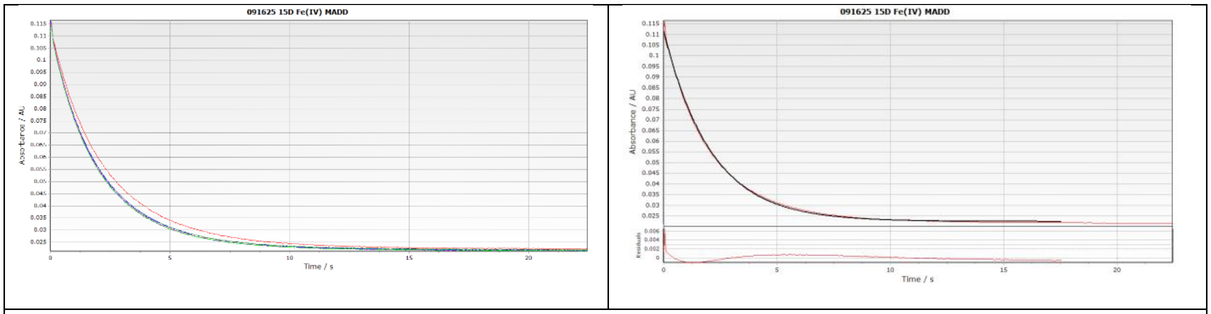

15°C D

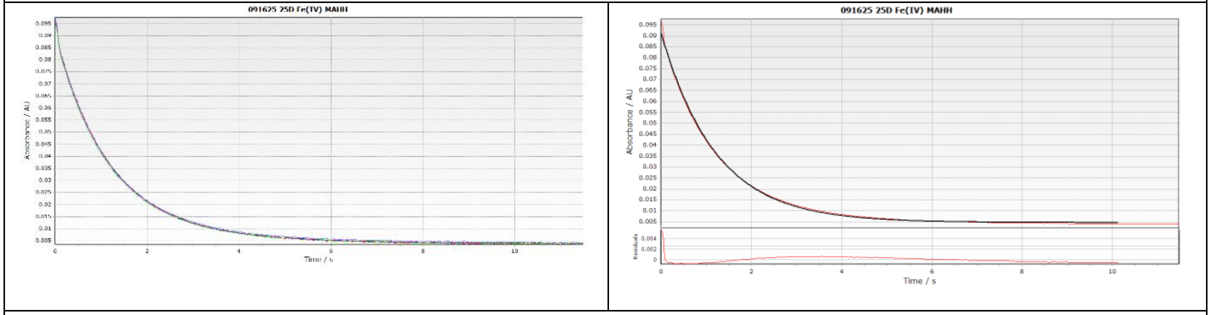

25°C D

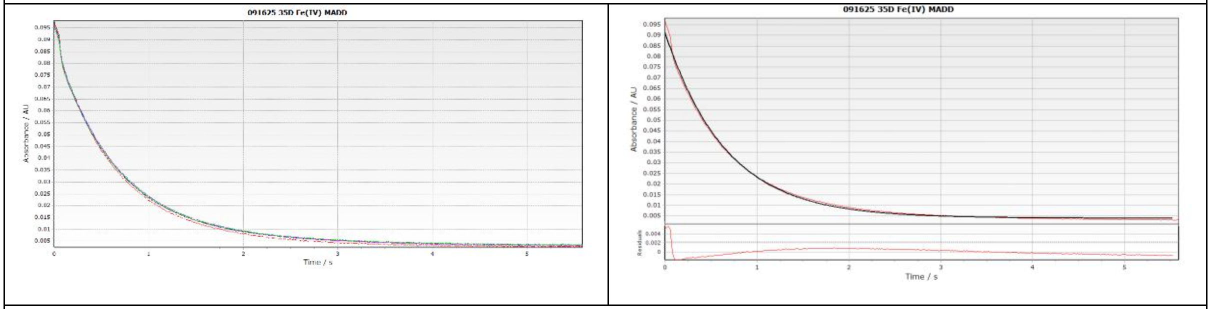

35°C D

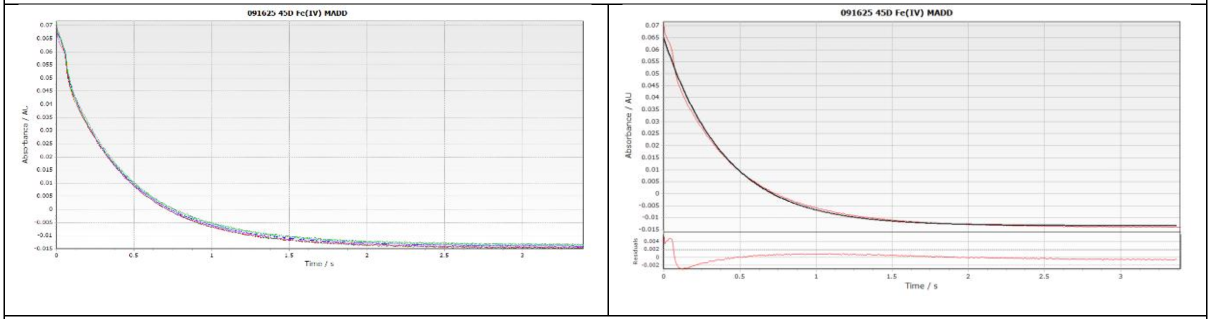

45°C D

Day 3 data
September 23, 2025)

	Pseudo-first-order rate constants k^{pfo} (s^{-1})									
**Temp (°C)**	**Trial H1**	**Trial H2**	**Trial H3**	**Trial H4**	**Trial H5**	**Trial H6**	**Average k_H^{pfo} (s^{-1})**	**Stdev**	**k_{2H} ($M^{-1}s^{-1}$)**	**Stdev [a]**
45	25.23052	26.06956	26.64828	25.82029	25.27651	26.18856	25.87229	5.50E-01	5.17E+03	1.10E+02
35	20.60824	19.43161	18.65862	18.23156	18.29422	18.54324	18.96125	9.14E-01	3.79E+03	1.83E+02
25	13.36704	13.44437	13.41885	13.54502	13.40327	13.30399	13.41376	8.07E-02	2.68E+03	1.61E+01
15	9.11667	9.20066	9.07185	9.05952	8.97536	8.83294	9.04283	1.27E-01	1.81E+03	2.53E+01
5	5.65182	5.67651	5.84094	5.85431	5.83305	5.80886	5.77758	8.94E-02	1.16E+03	1.79E+01
**Temp (°C)**	**Trial D1**	**Trial D2**	**Trial D3**	**Trial D4**	**Trial D5**	**Trial D6**	**Average k_D^{pfo} (s-1)**	**Stdev**	**k_{2D} (M-1s-1)**	**Stdev [a]**
45	2.55540	2.48639	2.37534	2.38320	2.41686	2.35464	2.4286	7.74E-02	4.86E+02	1.55E+01
35	1.41067	1.39794	1.41914	1.40789	1.40944	1.40142	1.4078	7.45E-03	2.82E+02	1.49E+00
25	0.85267	0.86366	0.84872	0.84728	0.82585	0.83315	0.8452	1.37E-02	1.69E+02	2.73E+00
15	0.50882	0.51526	0.50689	0.49905	0.49627	0.49160	0.5030	8.83E-03	1.01E+02	1.77E+00
5	0.24774	0.25357	0.25465	0.25470	0.25574	0.25763	0.2540	3.36E-03	5.08E+01	6.72E-01

[a] = (Stdev(for k^{pfo})/k^{pfo})*k_2

Six kinetic trials and the mean fit	First-order kinetic fitted average of six trials
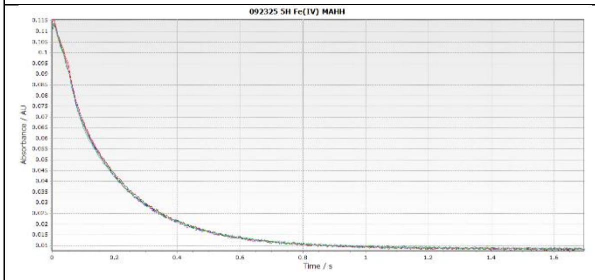	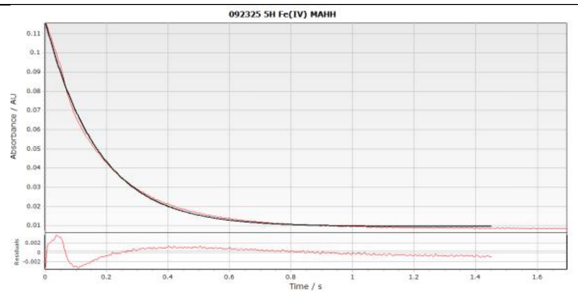
5°C H	
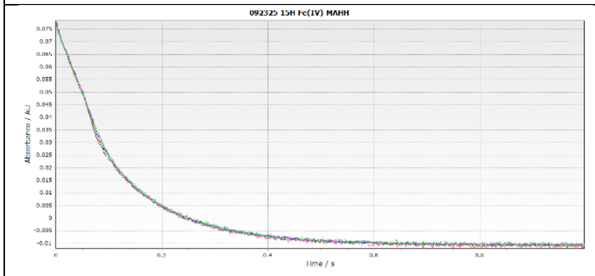	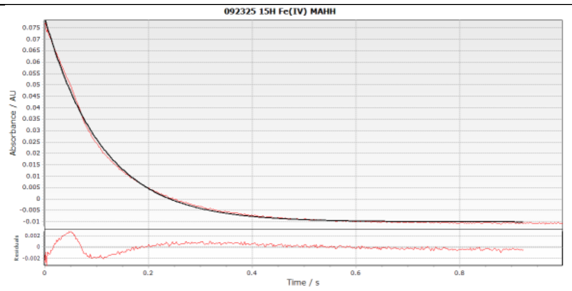
15°C H	

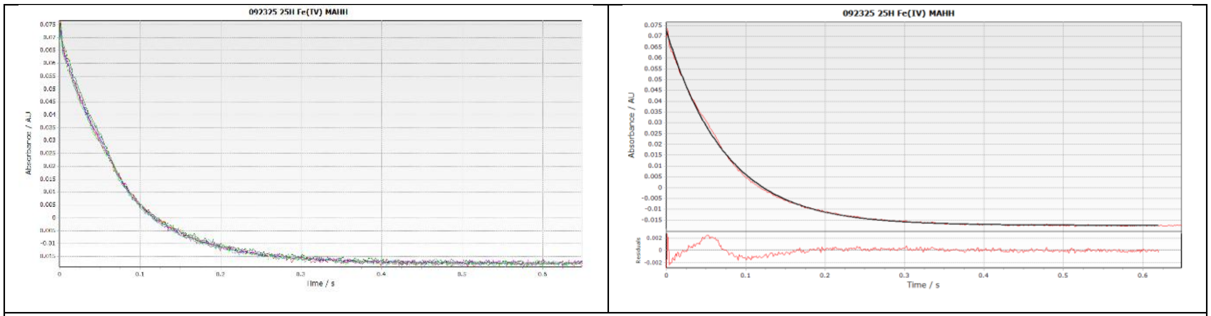

25°C H

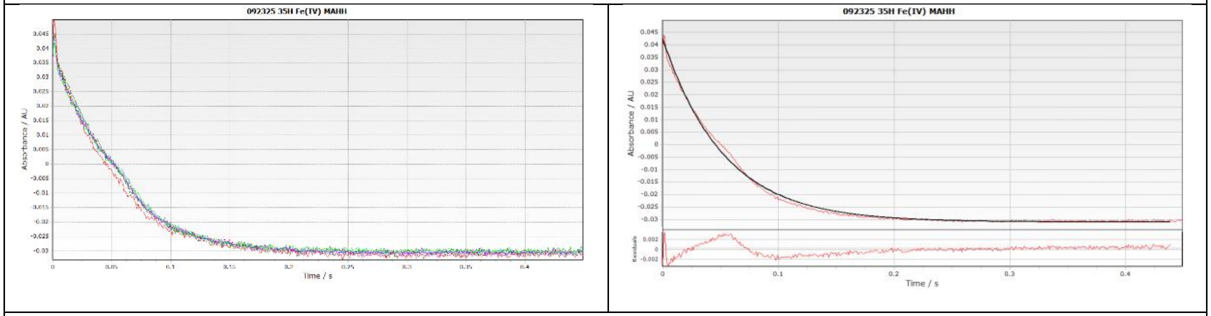

35°C H

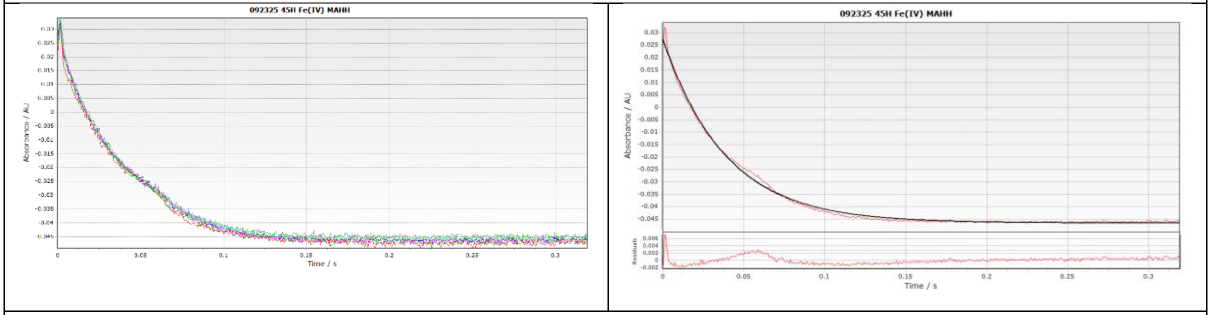

45°C H

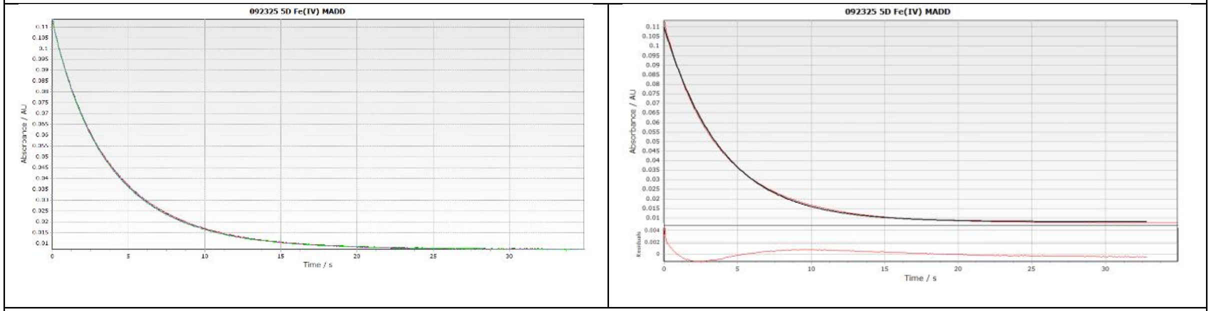

5°C D

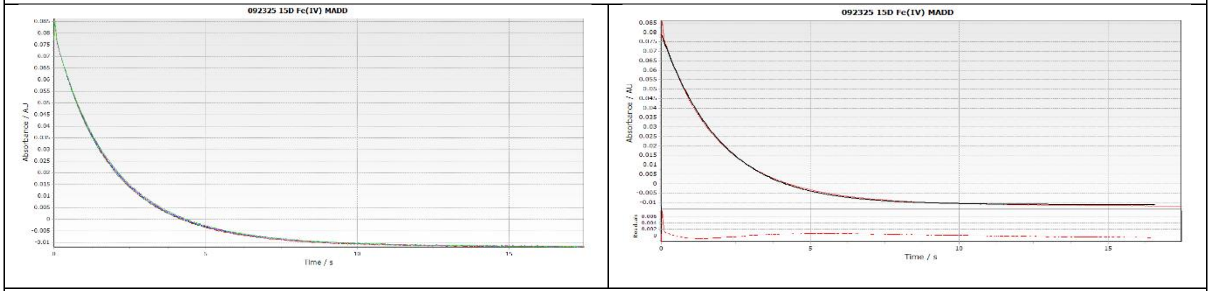

15°C D

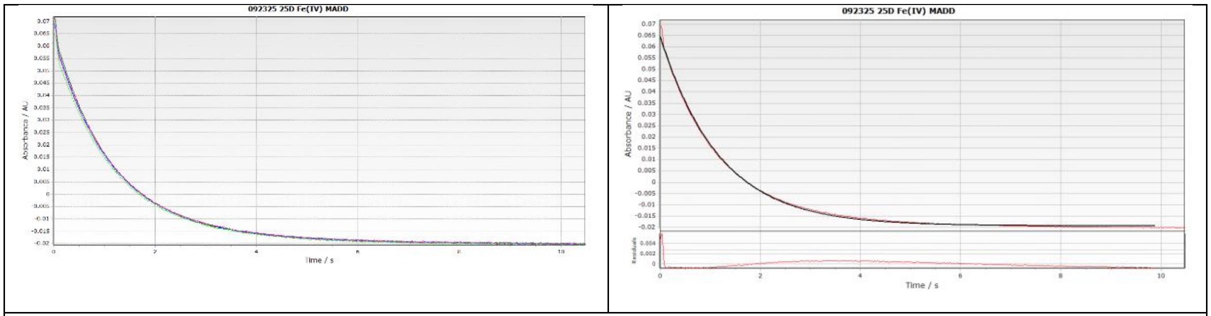

25°C D

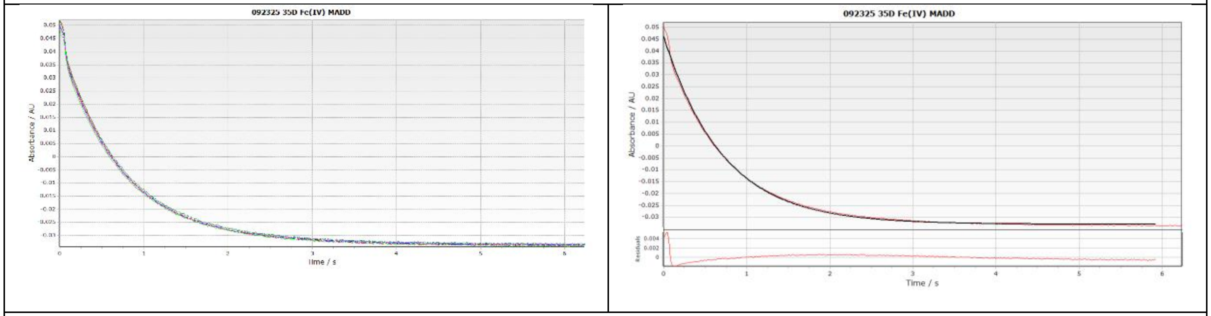

35°C D

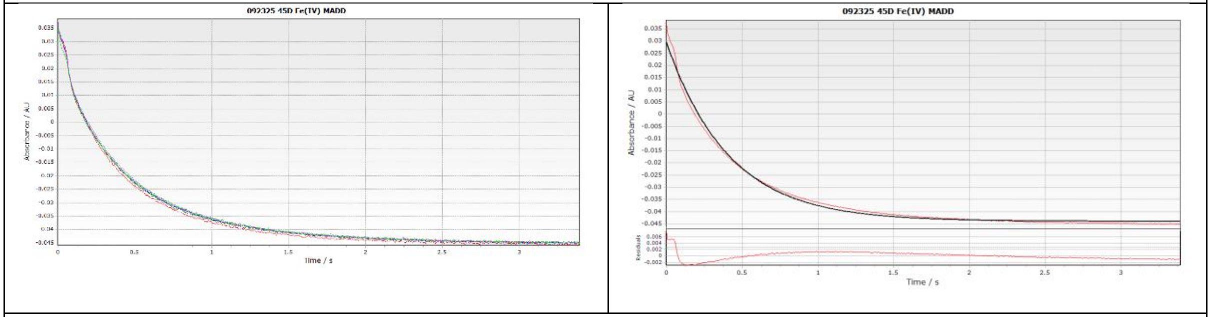

45°C D

Primary kinetic data for the rate constants in Table S5

Day 1 data (August 30, 2025)

				Pseudo-first-order rate constants k^{pfo} (s^{-1})						
**Temp (°C)**	**Trial H1**	**Trial H2**	**Trial H3**	**Trial H4**	**Trial H5**	**Trial H6**	**Average k_H^{pfo} (s^{-1})**	**Stdev**	**k_{2H} ($M^{-1}s^{-1}$)**	**Stdev [a]**
45	2.87527	2.99082	2.97399	3.10655	2.98239	2.91169	2.97345	7.94E-02	5.95E+02	1.59E+01
35	1.85276	1.83123	1.84172	1.83875	1.83168	1.84610	1.84037	8.36E-03	3.68E+02	1.67E+00
25	1.14895	1.20017	1.20901	1.21298	1.20046	1.20030	1.19531	2.33E-02	2.39E+02	4.67E+00
15	0.75506	0.74477	0.76012	0.75867	0.76615	0.75700	0.75696	7.07E-03	1.51E+02	1.41E+00
5	0.43453	0.42368	0.42761	0.43092	0.43228	0.42256	0.42860	4.81E-03	8.57E+01	9.62E-01
**Temp (°C)**	**Trial D1**	**Trial D2**	**Trial D3**	**Trial D4**	**Trial D5**	**Trial D6**	**Average k_D^{pfo} (s-1)**	**Stdev**	**k_{2D} (M-1s-1)**	**Stdev [a]**
45	0.38227	0.38211	0.38021	0.37215	0.38048	0.38231	0.3799	3.92E-03	7.60E+01	7.84E-01

35	0.20427	0.19652	0.19953	0.19870	0.19981	0.19848	0.1996	2.58E-03	3.99E+01	5.17E-01
25	0.10746	0.10642	0.10837	0.10637	0.10559	0.10586	0.1067	1.05E-03	2.13E+01	2.10E-01
15	0.04583	0.04793	0.05031	0.05181	0.05173	0.05228	0.0500	2.58E-03	1.00E+01	5.16E-01
5	0.02414	0.02564	0.02656	0.0262	0.03141	0.02612	0.0267	2.47E-03	5.34E+00	4.94E-01

[a] = (Stdev(for k^{pfo})/k^{pfo})*k_2

Six kinetic trials and the mean fit	First-order kinetic fitted average of six trials
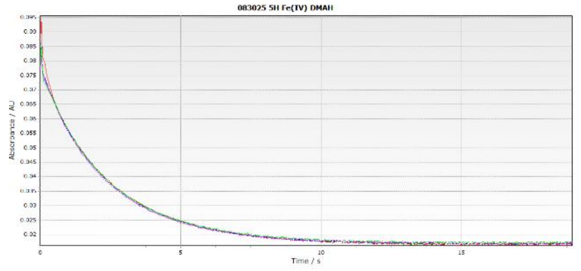	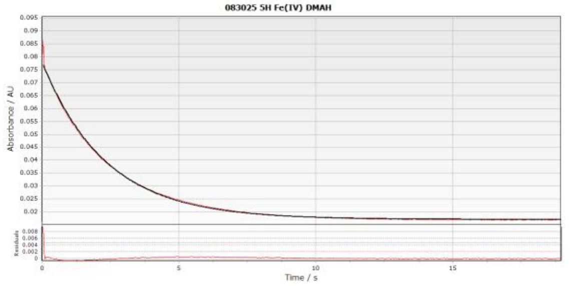
5°C H	
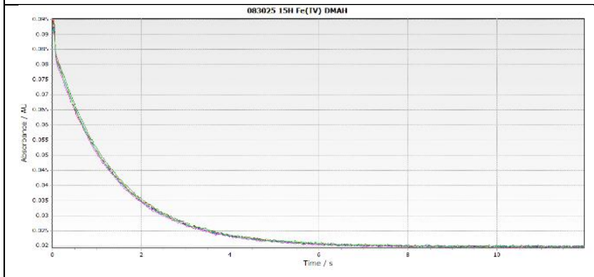	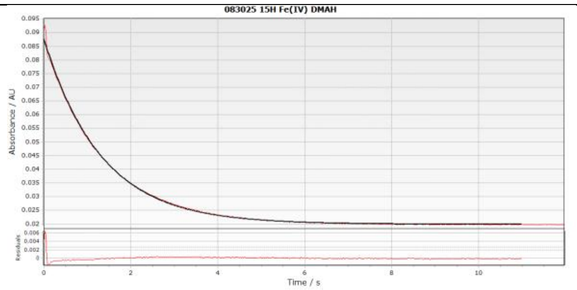
15°C H	
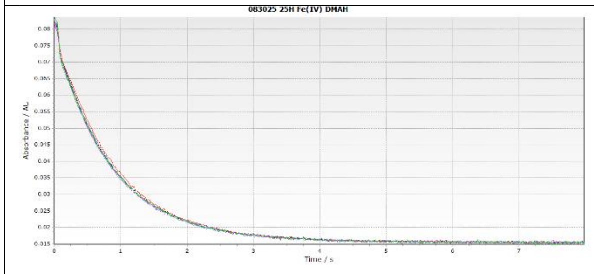	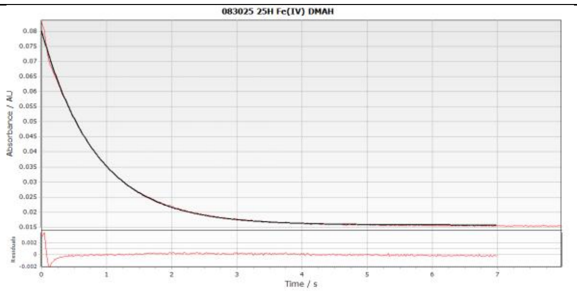
25°C H	
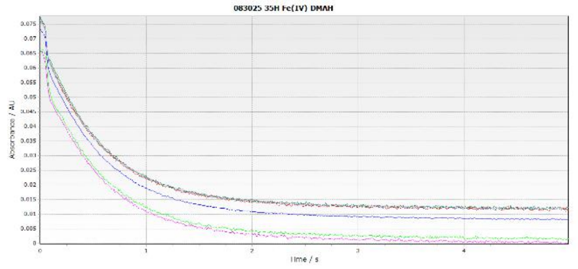	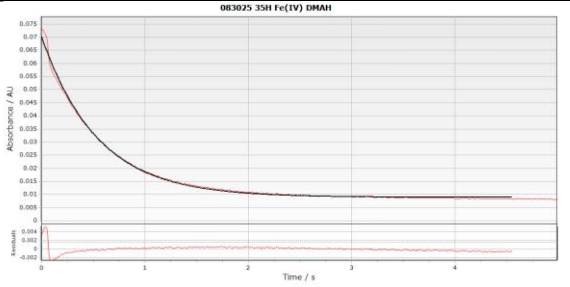
35°C H	

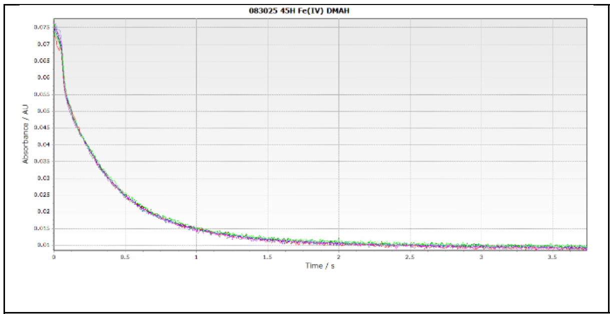

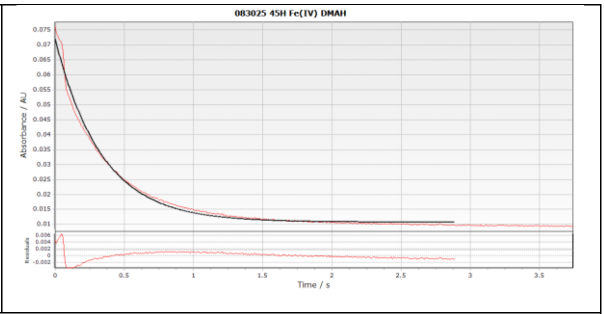

45°C H

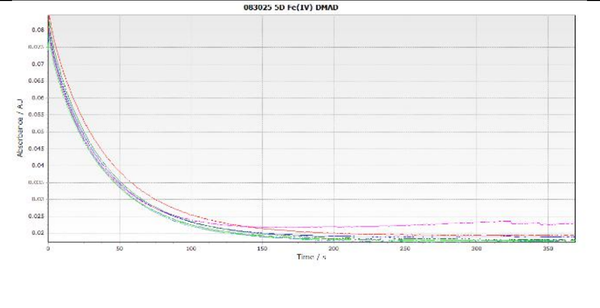

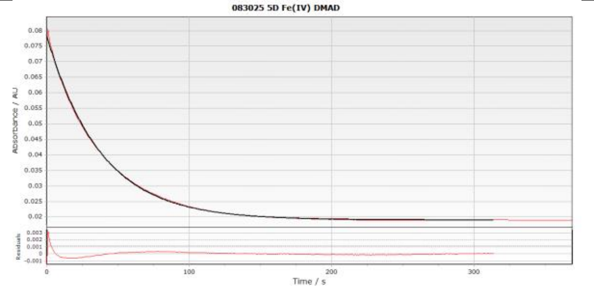

5°C D

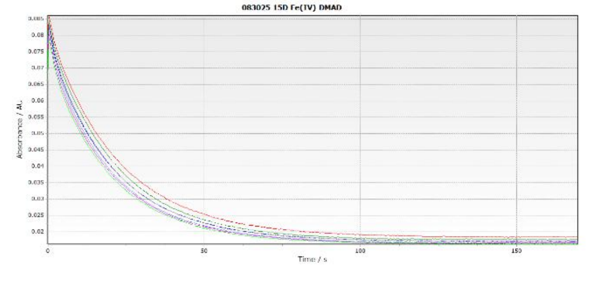

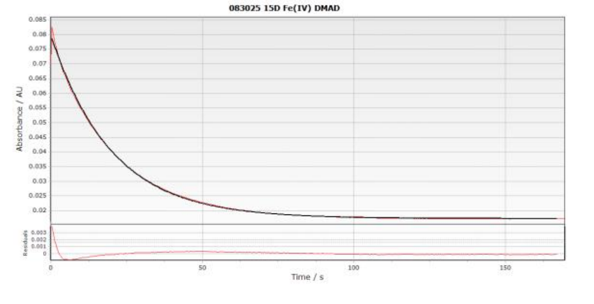

15°C D

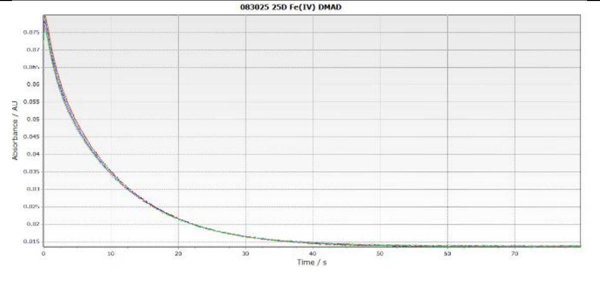

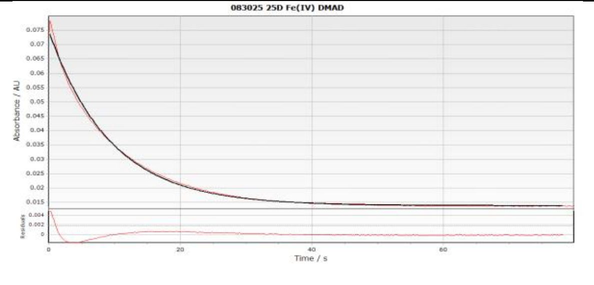

25°C D

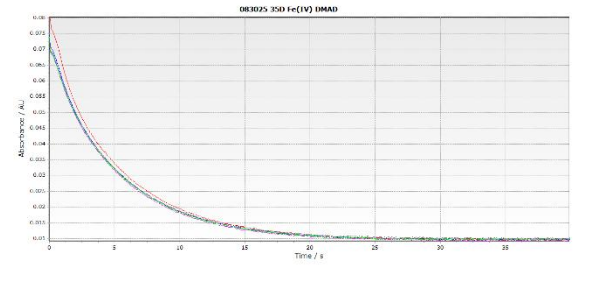

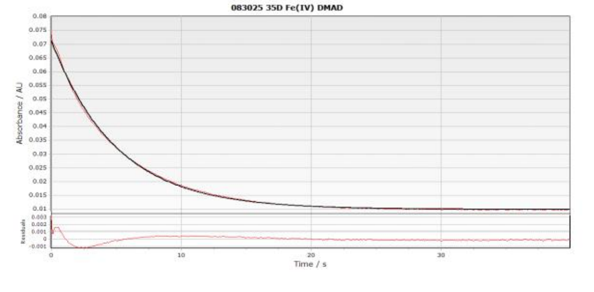

35°C D

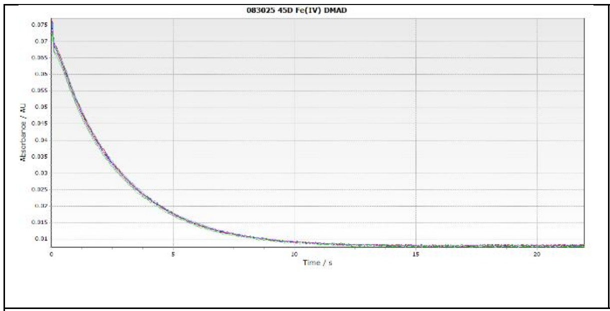	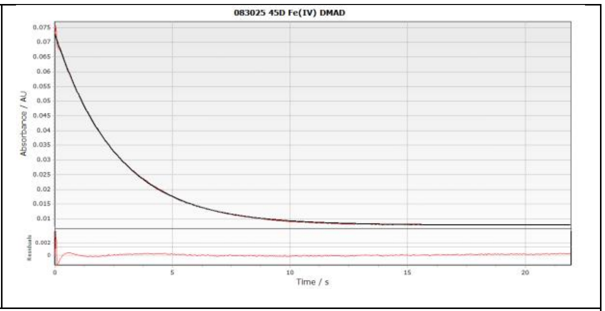
45°C D	

Day 2 data (September 08, 2025)

				Pseudo-first-order rate constants k^{pfo} (s^{-1})						
**Temp (°C)**	**Trial H1**	**Trial H2**	**Trial H3**	**Trial H4**	**Trial H5**	**Trial H6**	**Average k_H^{pfo} (s^{-1})**	**Stdev**	**k_{2H} ($M^{-1}s^{-1}$)**	**Stdev [a]**
45	2.85072	2.85991	2.80773	2.91146	2.88232	2.91473	2.87115	4.05E-02	5.74E+02	8.11E+00
35	1.88261	1.88564	1.90442	1.88494	1.89758	1.92094	1.89602	1.49E-02	3.79E+02	2.97E+00
25	1.13966	1.17382	1.17703	1.15039	1.17988	1.16920	1.16500	1.62E-02	2.33E+02	3.25E+00
15	0.62403	0.69490	0.72165	0.72611	0.72982	0.72854	0.70418	4.13E-02	1.41E+02	8.27E+00
5	0.39278	0.41789	0.42409	0.42171	0.41883	0.42492	0.41670	1.20E-02	8.33E+01	2.41E+00
**Temp (°C)**	**Trial D1**	**Trial D2**	**Trial D3**	**Trial D4**	**Trial D5**	**Trial D6**	**Average k_D^{pfo} (s-1)**	**Stdev**	**k_{2D} (M-1s-1)**	**Stdev [a]**
45	0.39702	0.39419	0.39100	0.39217	0.38555	0.38881	0.3915	4.03E-03	7.83E+01	8.05E-01
35	0.20709	0.19989	0.19776	0.19775	0.20072	0.19663	0.2000	3.80E-03	4.00E+01	7.60E-01
25	0.09588	0.09862	0.10170	0.09847	0.10084	0.09945	0.0992	2.04E-03	1.98E+01	4.09E-01
15	0.04580	0.04830	0.04912	0.04958	0.04900	0.04925	0.0485	1.39E-03	9.70E+00	2.78E-01
5	0.02234	0.02267	0.02295	0.02373	0.02358	0.02354	0.0231	5.65E-04	4.63E+00	1.13E-01

[a] = (Stdev(for k^{pfo})/k^{pfo})*k_2

Six kinetic trials and the mean fit	First-order kinetic fitted average of six trials
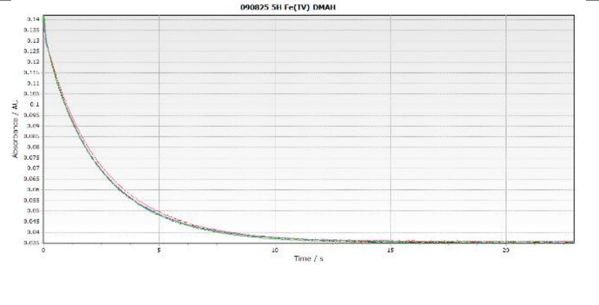	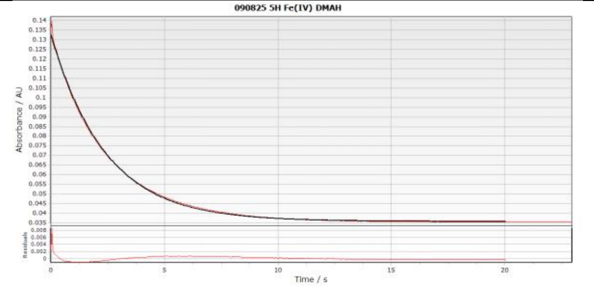
5°C H	

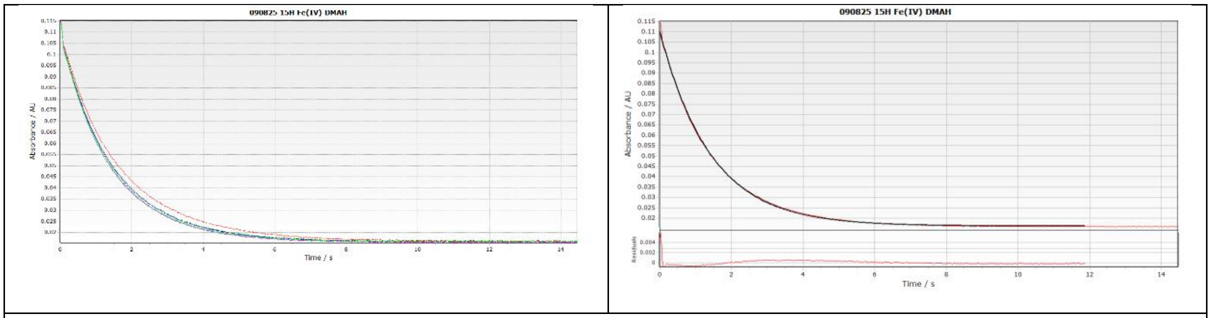

15°C H

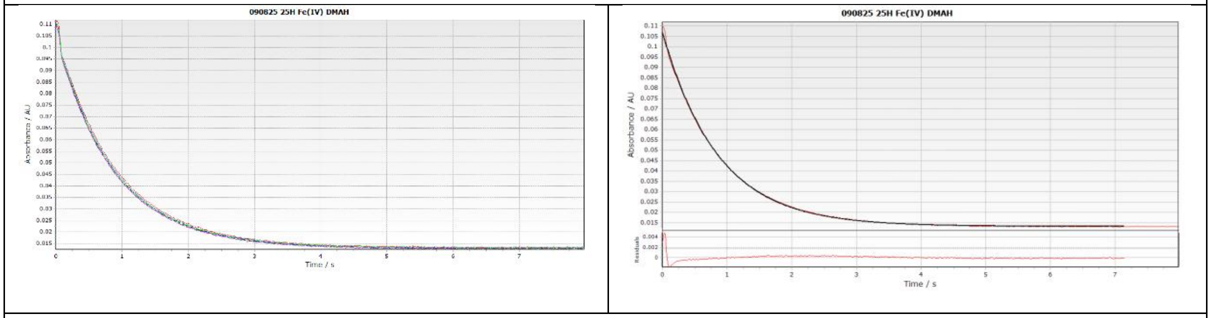

25°C H

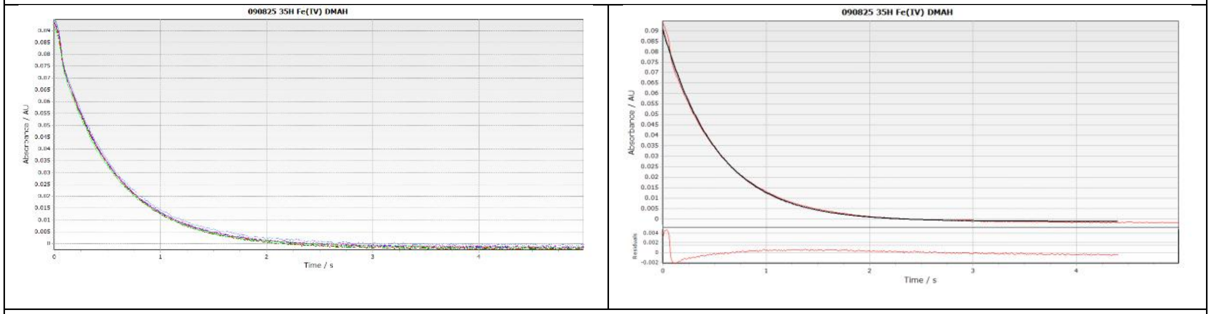

35°C H

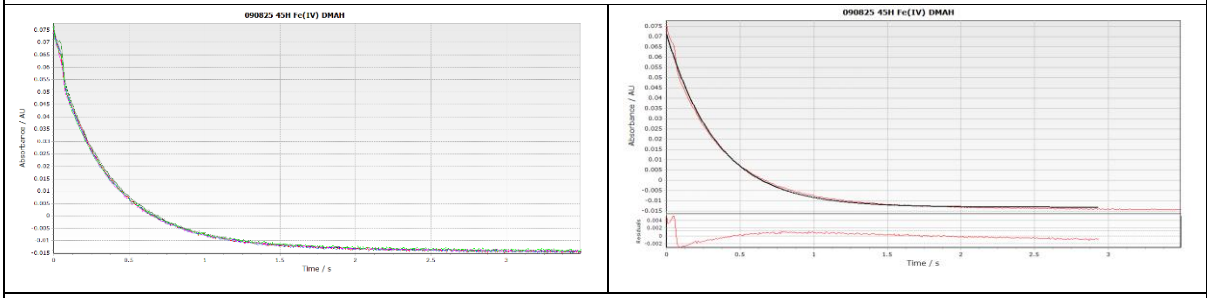

45°C H

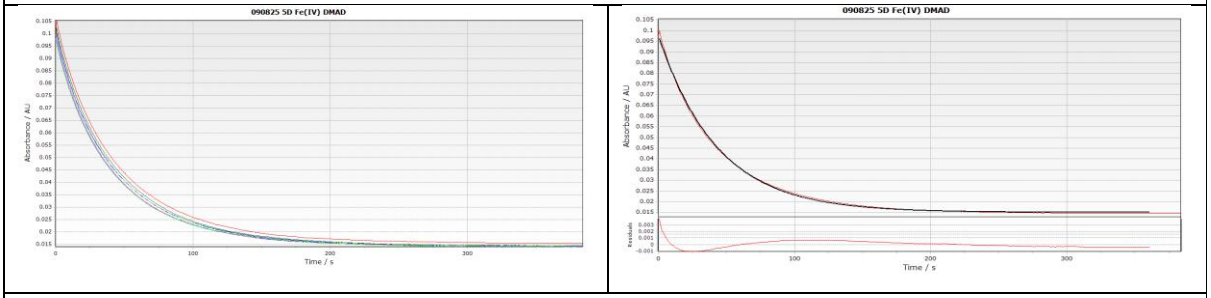

5°C D

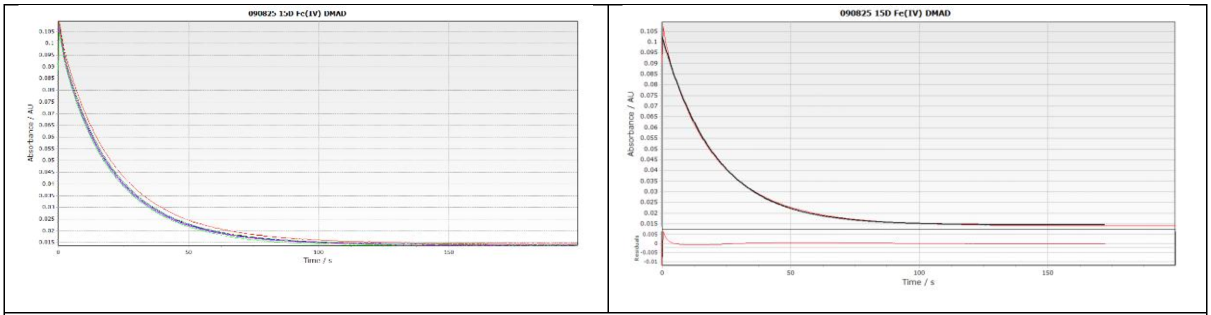

15°C D

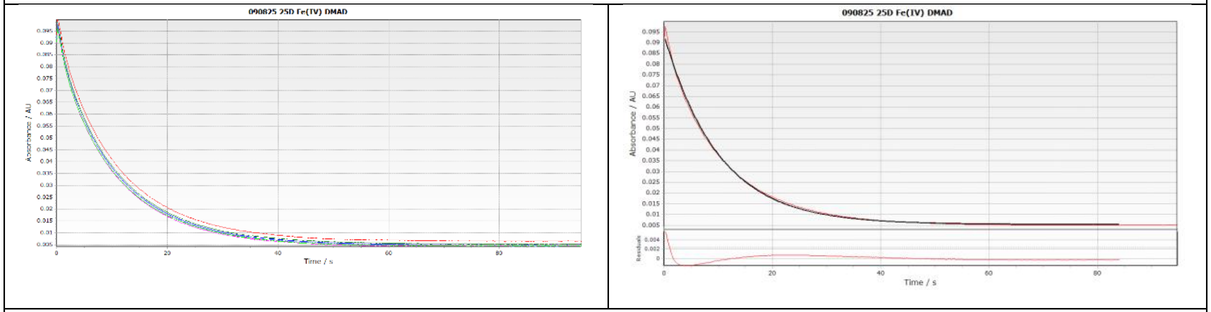

25°C D

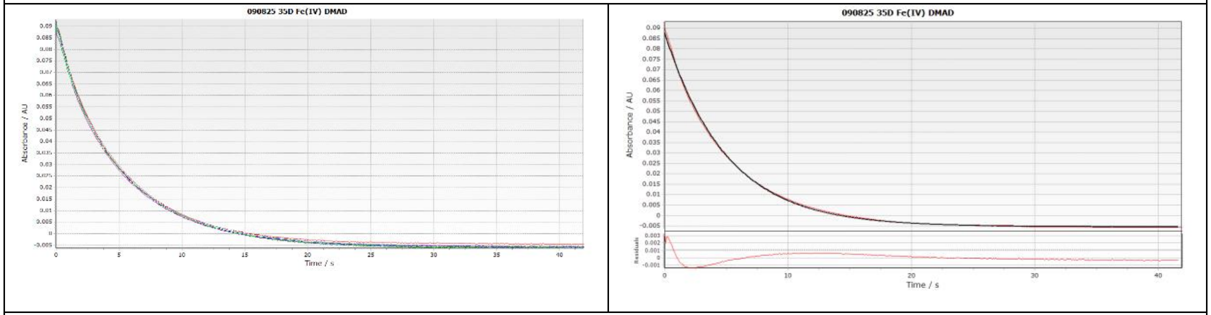

35°C D

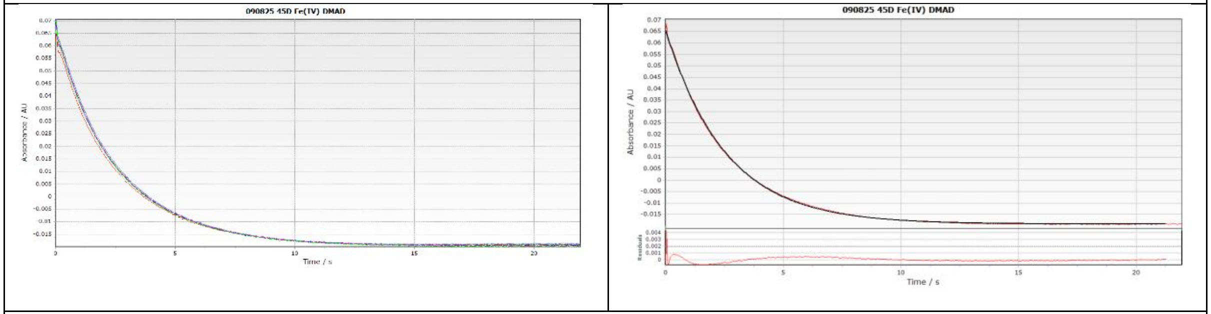

45°C D

Day 3 data (September 09, 2025)

	Pseudo-first-order rate constants k^{pfo} (s^{-1})									
**Temp (°C)**	**Trial H1**	**Trial H2**	**Trial H3**	**Trial H4**	**Trial H5**	**Trial H6**	**Average k_H^{pfo} (s^{-1})**	**Stdev**	**k_{2H} ($M^{-1}s^{-1}$)**	**Stdev [a]**
45	2.78578	2.79316	2.77432	2.81886	2.69780	2.64316	2.75218	6.72E-02	5.50E+02	1.34E+01

35	1.84221	1.79384	1.84738	1.86223	1.82833	1.84407	1.83634	2.35E-02	3.67E+02	4.70E+00
25	1.10474	1.16606	1.18703	1.18874	1.18082	1.17762	1.16750	3.18E-02	2.34E+02	6.36E+00
15	0.67915	0.71493	0.71682	0.72406	0.72584	0.72307	0.71398	1.76E-02	1.43E+02	3.52E+00
5	0.41940	0.42199	0.42006	0.42088	0.42147	0.42372	0.42125	1.53E-03	8.43E+01	3.06E-01

**Temp (°C)**	**Trial D1**	**Trial D2**	**Trial D3**	**Trial D4**	**Trial D5**	**Trial D6**	**Average k_D^{pfo} (s-1)**	**Stdev**	**k_{2D} (M-1s-1)**	**Stdev [a]**
45	0.35874	0.36337	0.36643	0.36377	0.37052	0.37672	0.3666	6.29E-03	7.33E+01	1.26E+00
35	0.18954	0.19182	0.19295	0.18728	0.18746	0.19056	0.1899	2.30E-03	3.80E+01	4.59E-01
25	0.10163	0.10300	0.10282	0.10618	0.10589	0.10648	0.1043	2.09E-03	2.09E+01	4.18E-01
15	0.04747	0.04751	0.04876	0.04895	0.04832	0.04870	0.0483	6.49E-04	9.66E+00	1.30E-01
5	0.02287	0.02305	0.02319	0.02318	0.02372	0.02327	0.0232	2.85E-04	4.64E+00	5.70E-02

[a] = (Stdev(for k^{pfo})/k^{pfo})*k_2

Six kinetic trials and the mean fit	First-order kinetic fitted average of six trials

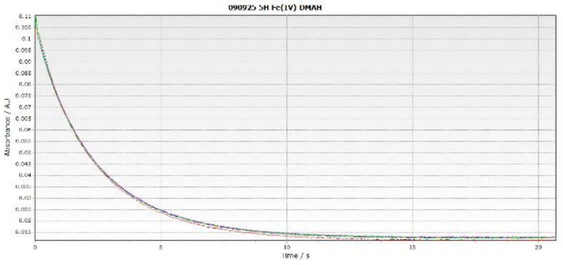

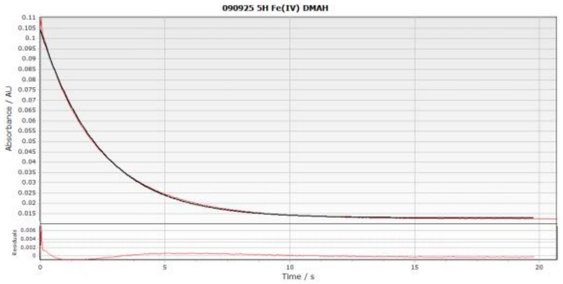

5°C H

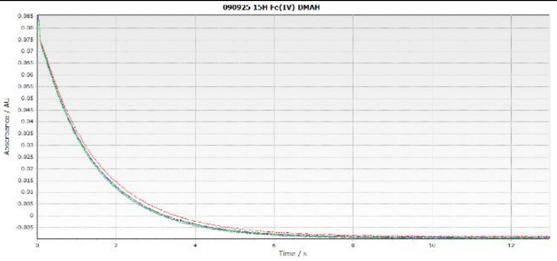

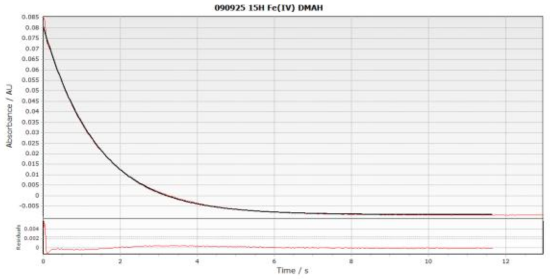

15°C H

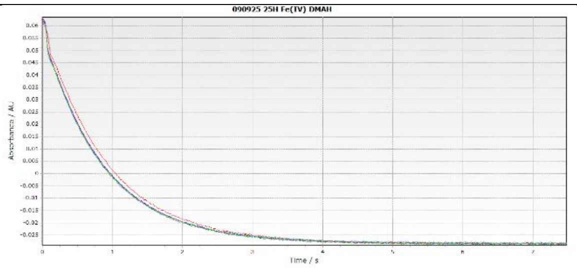

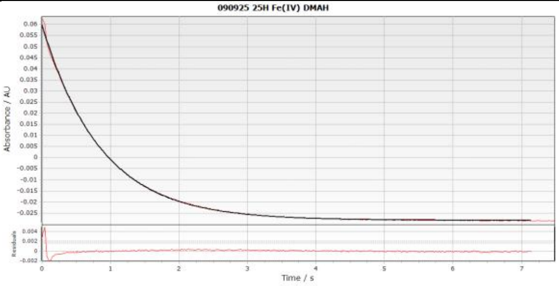

25°C H

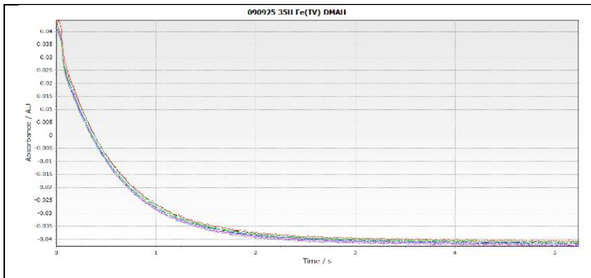

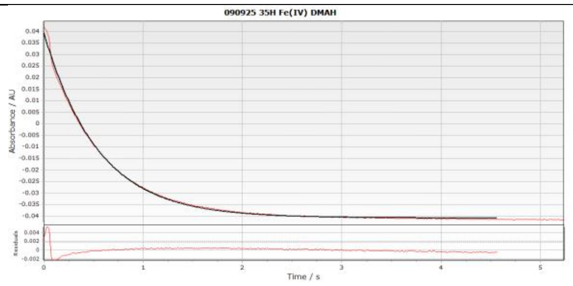

35°C H

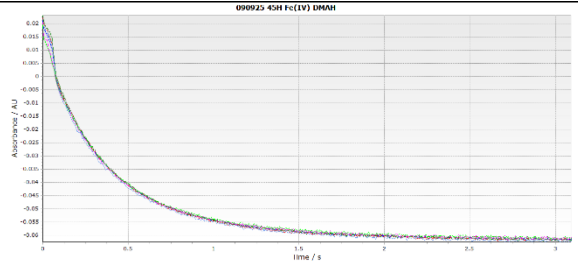

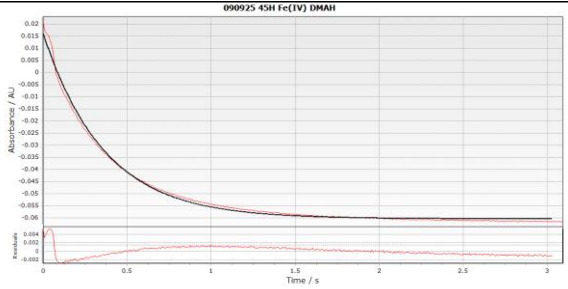

45°C H

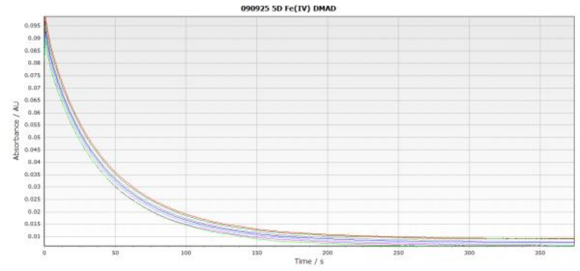

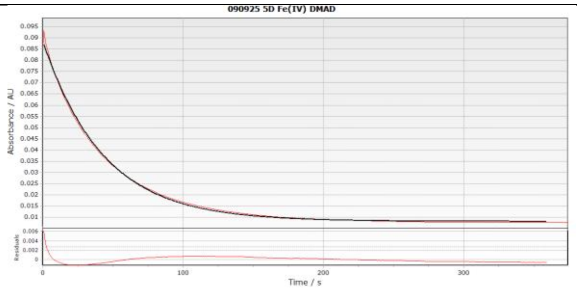

5°C D

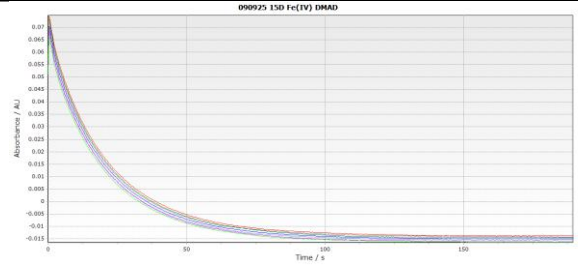

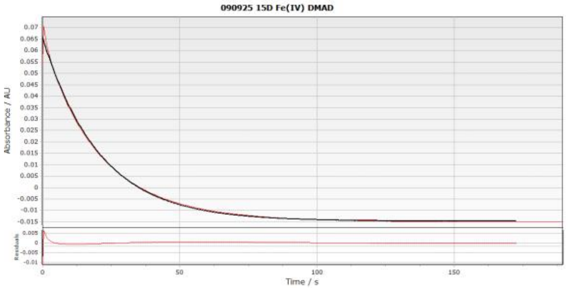

15°C D

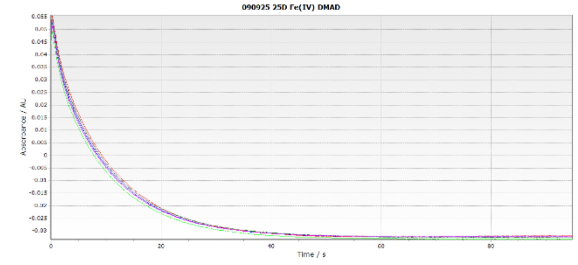

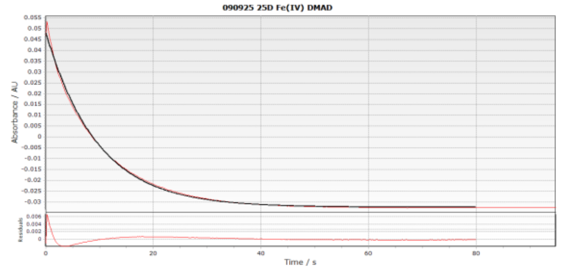

25°C D

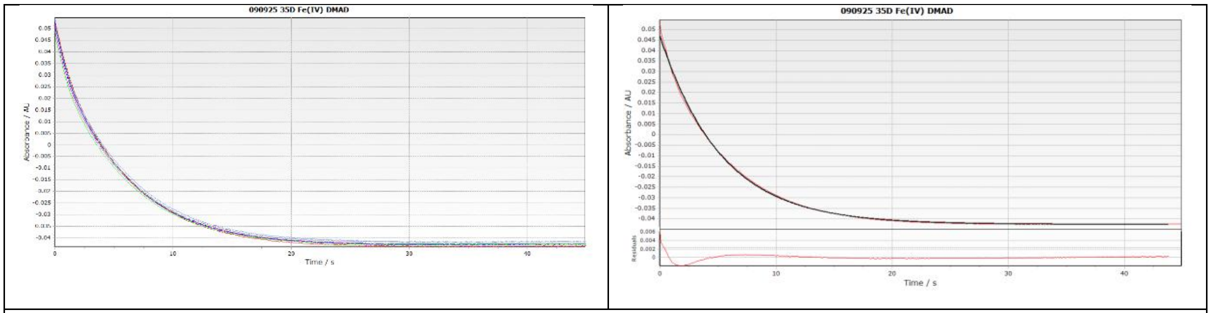

35°C D

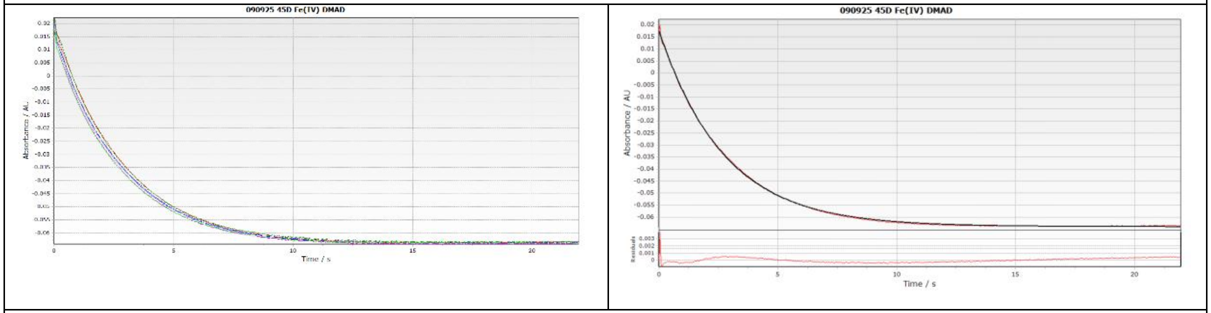

45°C D

Primary kinetic data for the rate constants in Table S6

Day 1 data (August 26, 2025)

Pseudo-first-order rate constants k^{pfo} (s^{-1})

**Temp (°C)**	**Trial H1**	**Trial H2**	**Trial H3**	**Average k_H^{pfo} (s^{-1})**	**Stdev**	**k_{2H} ($M^{-1}s^{-1}$)**	**Stdev [a]**
45	1.86639	1.84754	1.82158	1.84517	2.25E-02	3.69E+02	4.50E+00
35	1.22778	1.25233	1.25353	1.24455	1.45E-02	2.49E+02	2.91E+00
25	0.86910	0.88500	0.88100	0.87837	8.27E-03	1.76E+02	1.65E+00
15	0.51085	0.53568	0.54316	0.52990	1.69E-02	1.06E+02	3.38E+00
5	0.30881	0.32628	0.32628	0.32046	1.01E-02	6.41E+01	2.02E+00
**Temp (°C)**	**Trial D1**	**Trial D2**	**Trial D3**	**Average k_D^{pfo} (s^{-1})**	**Stdev**	**k_{2D} ($M^{-1}s^{-1}$)**	**Stdev [a]**
55	0.30476	0.30504	0.30323	0.3043	9.74E-04	6.09E+01	1.95E-01
45	0.14640	0.14514	0.14577	0.1458	6.30E-04	2.92E+01	1.26E-01
35	0.07385	0.07329	0.07297	0.0734	4.45E-04	1.47E+01	8.91E-02
25	0.03300	0.03373	0.03404	0.0336	5.34E-04	6.72E+00	1.07E-01
15	0.01213	0.01382	0.01468	0.0135	1.30E-03	2.71E+00	2.59E-01

[a] = (Stdev(for k^{pfo})/k^{pfo})*k_2

Three kinetic trials together with the average	First-order kinetic fitted average of three trials
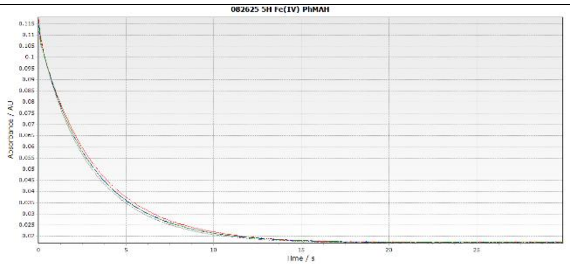	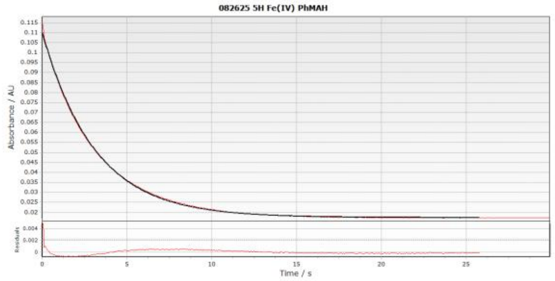
5℃ H	
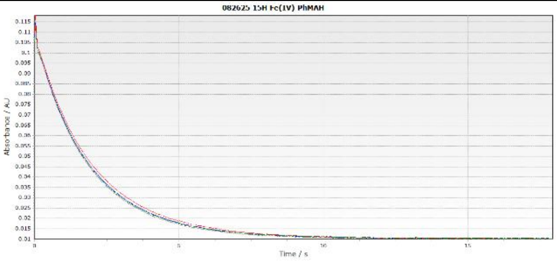	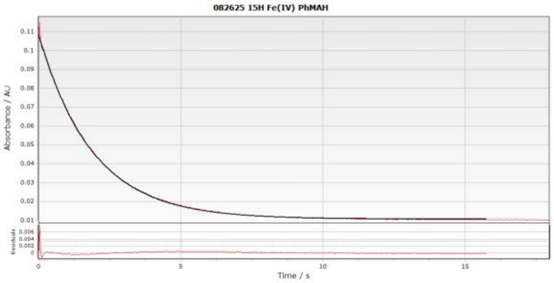
15℃ H	
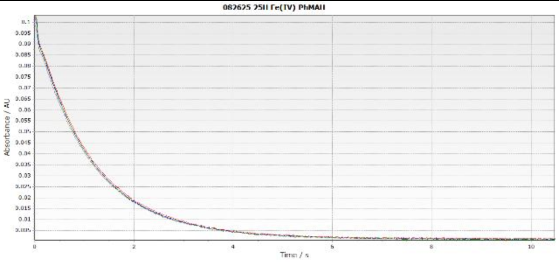	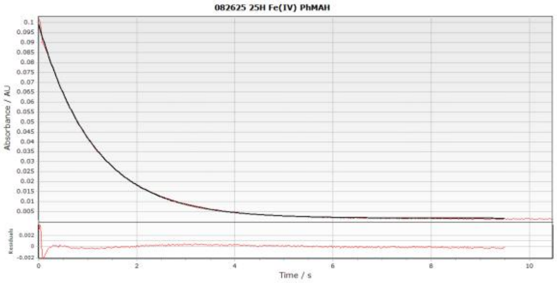
25℃ H	
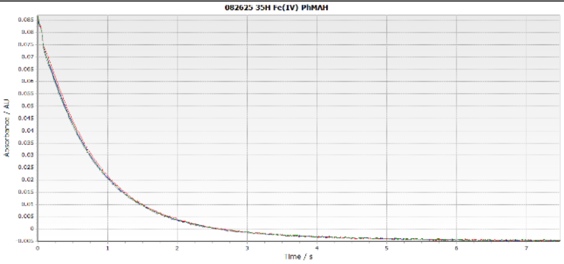	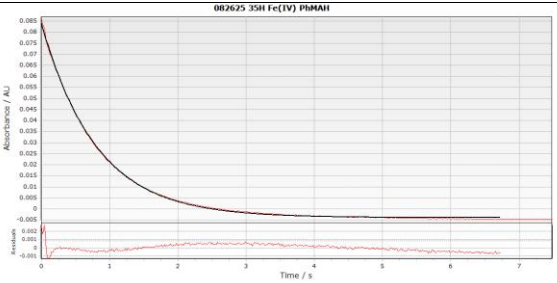
35℃ H	

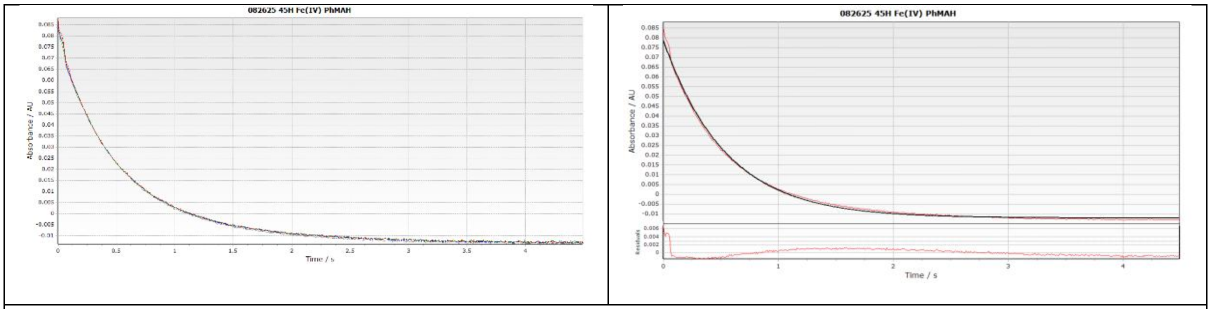

45°C H

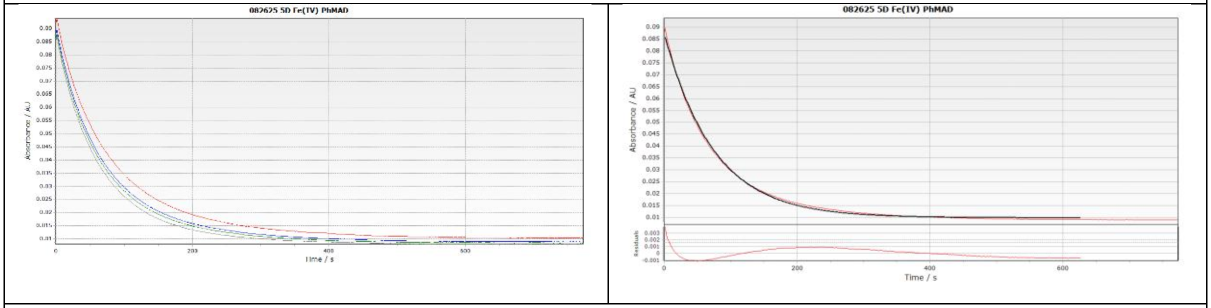

5°C D

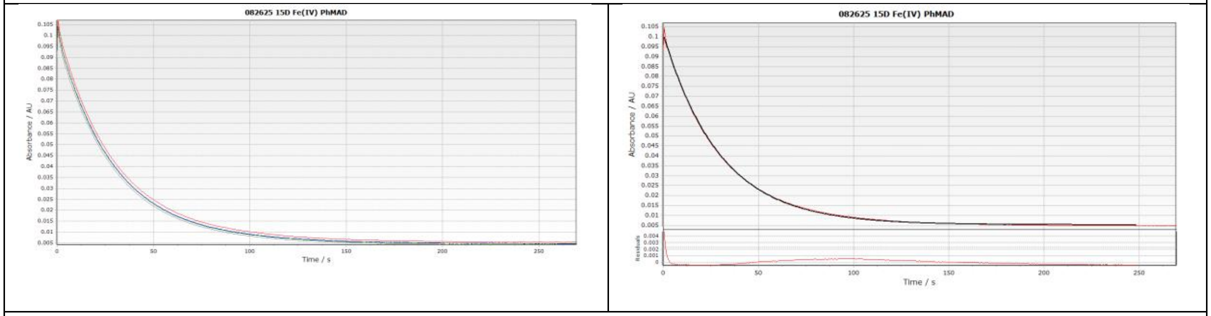

15°C D

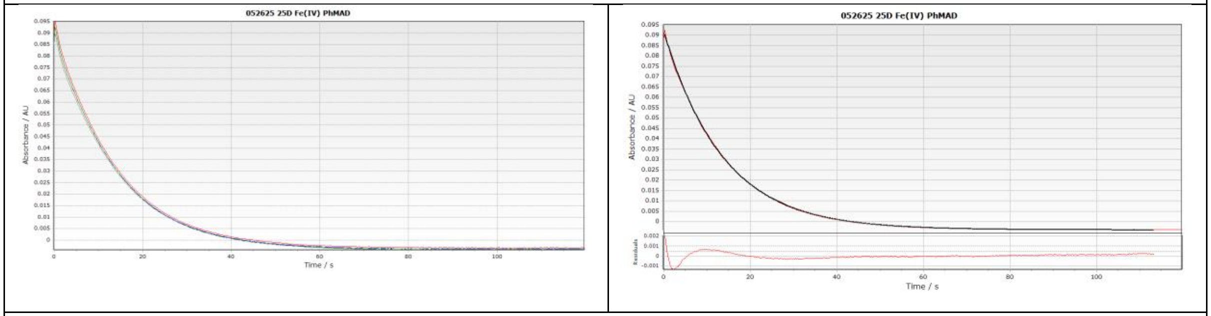

25°C D

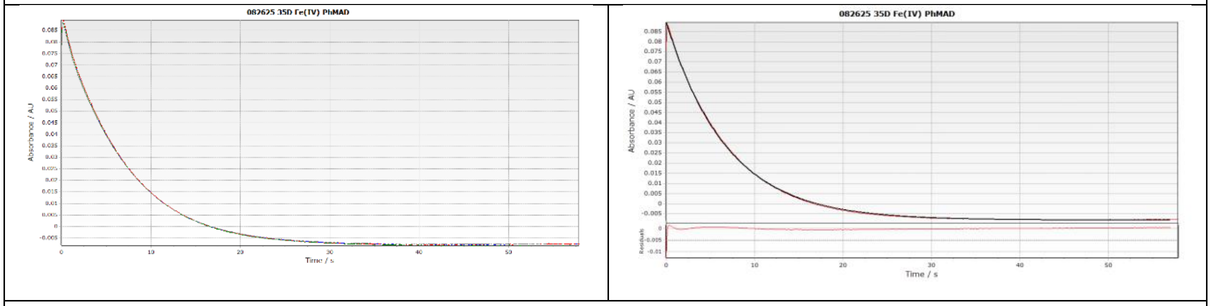

35°C D

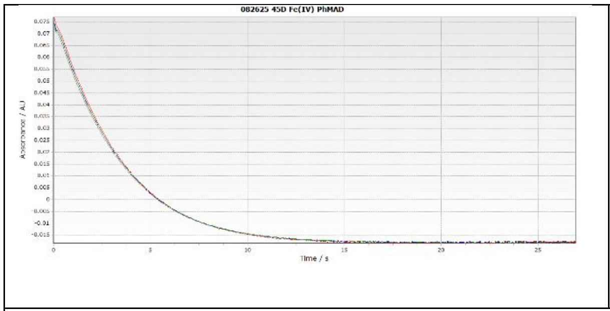

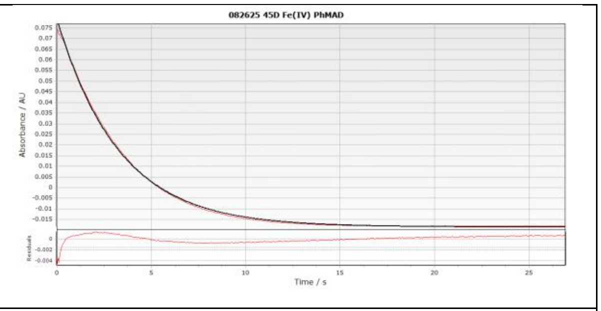

45℃ D

Day 2 data (September 02, 2025)

Temp (°C)	Trial H1	Trial H2	Trial H3	Trial H4	Trial H5	Trial H6	Average k_H^{pfo} (s^{-1})	Stdev	k_{2H} ($M^{-1}s^{-1}$)	Stdev [a]
	Pseudo-first-order rate constants k^{pfo} (s^{-1})									
45	1.96725	1.90553	1.95096	1.95928	1.98469	1.90977	1.94125	3.20E-02	3.88E+02	6.40E+00
35	1.18589	1.27641	1.30776	1.30939	1.30805	1.31510	1.25669	6.33E-02	2.51E+02	1.27E+01
25	0.86496	0.86808	0.87421	0.86844	0.86704	0.86640	0.86908	4.71E-03	1.74E+02	9.41E-01
15	0.52225	0.52471	0.52616	0.52488	0.52980	0.52174	0.52437	1.98E-03	1.05E+02	3.95E-01
5	0.27102	0.28917	0.31556	0.31880	0.32156	0.31630	0.29192	2.24E-02	5.84E+01	4.48E+00
**Temp (°C)**	**Trial D1**	**Trial D2**	**Trial D3**	**Trial D4**	**Trial D5**	**Trial D6**	**Average k_D^{pfo} (s-1)**	**Stdev**	**k_{2D} (M-1s-1)**	**Stdev [a]**
45	0.31050	0.29917	0.30280	0.30350	0.27116	0.29793	0.3042	5.79E-03	6.08E+01	1.16E+00
35	0.14120	0.14476	0.14123	0.14116	0.14238	0.14513	0.1424	2.05E-03	2.85E+01	4.09E-01
25	0.06429	0.06928	0.06921	0.06901	0.06926	0.07256	0.0676	2.86E-03	1.35E+01	5.72E-01
15	0.02825	0.03119	0.03212	0.03234	0.03234	0.03212	0.0305	2.02E-03	6.10E+00	4.04E-01
5	0.01391	0.01497	0.01525	0.01508	0.01529	0.01567	0.0147	7.07E-04	2.94E+00	1.41E-01

[a] = (Stdev(for k^{pfo})/k^{pfo})*k_2

Six kinetic trials and the mean fit	First-order kinetic fitted average of six trials
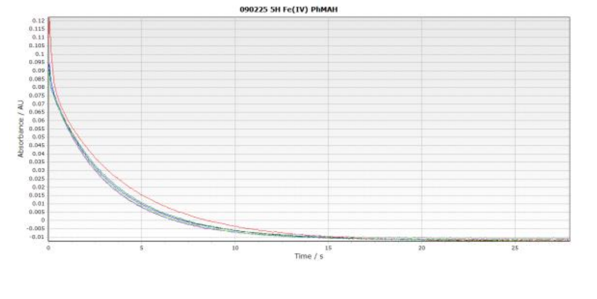	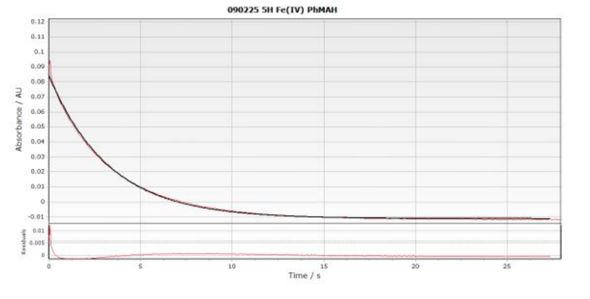

5℃ H

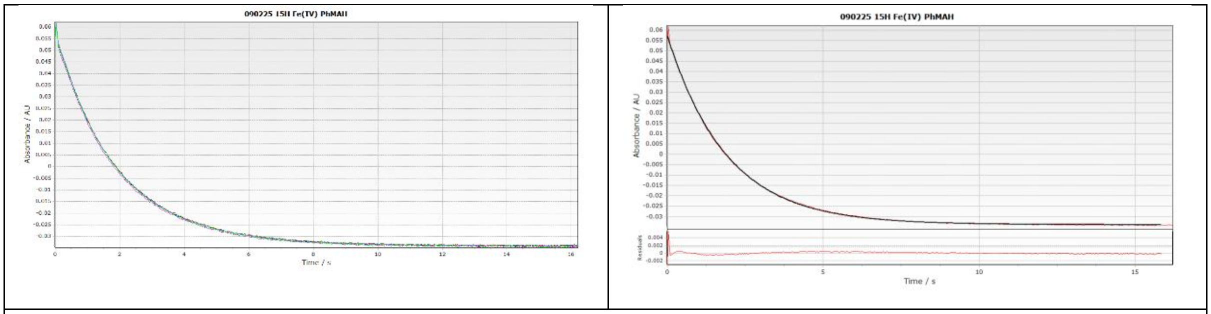

15°C H

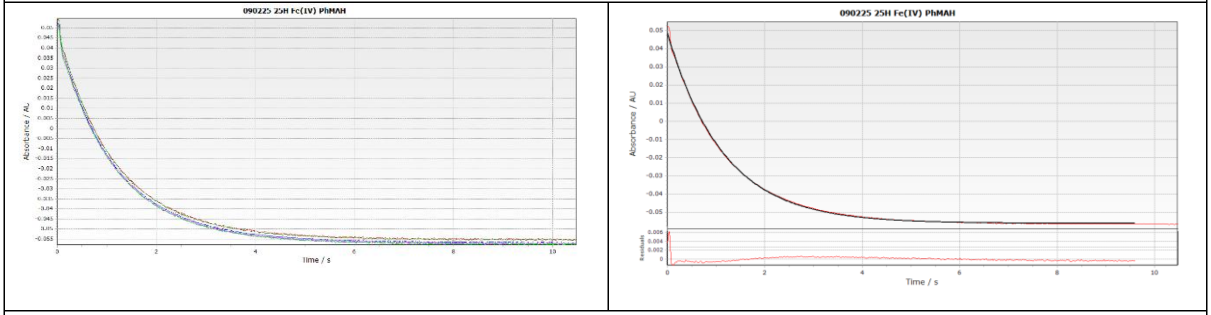

25°C H

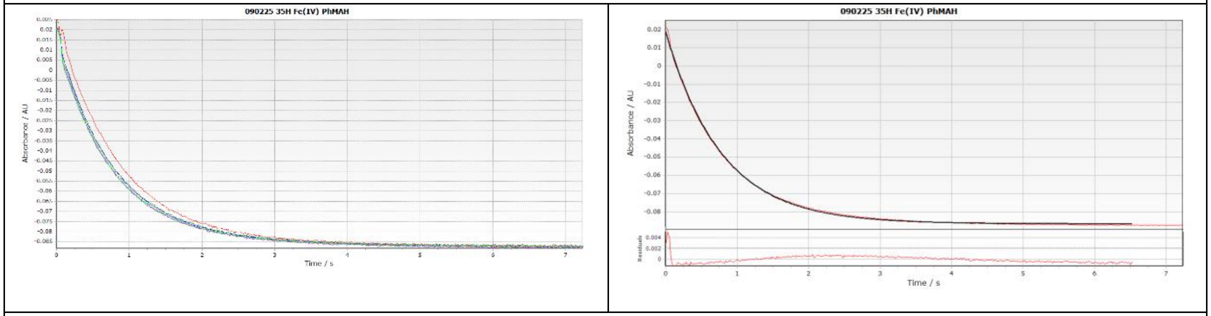

35°C H

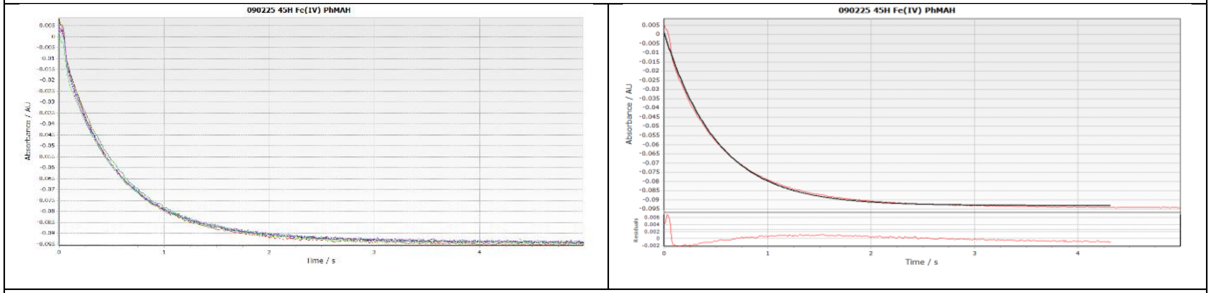

45°C H

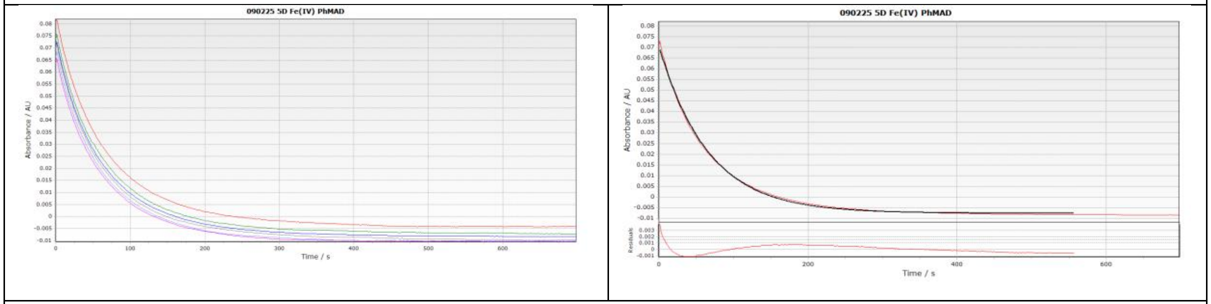

5°C D

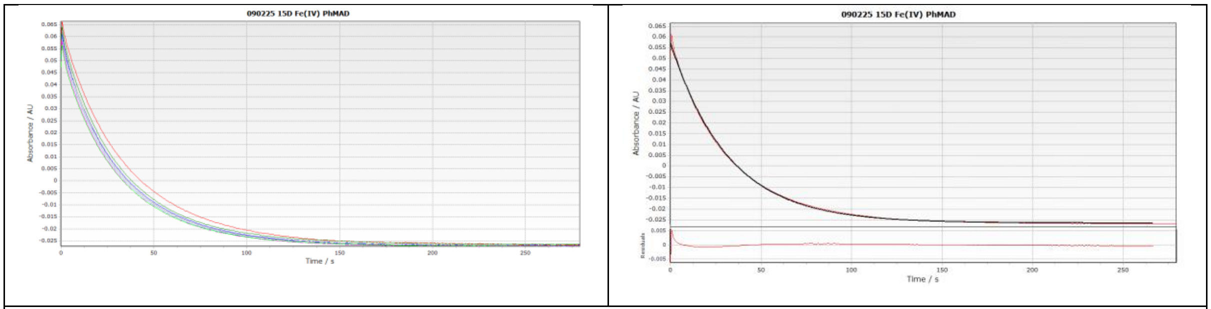

15°C D

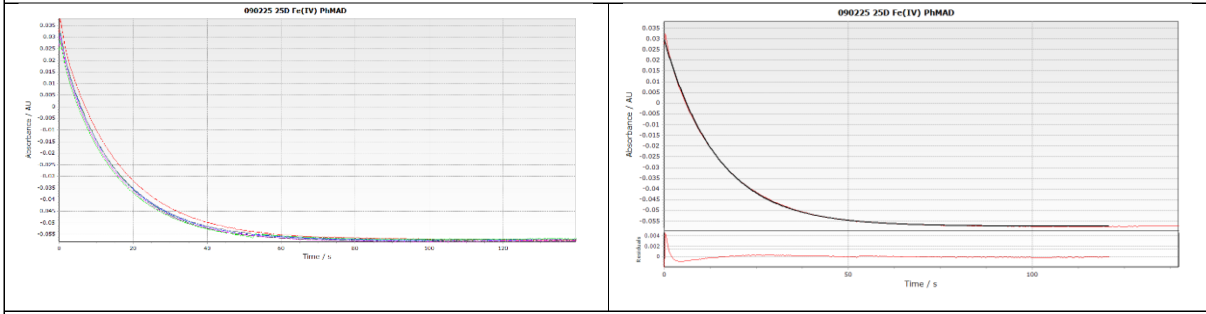

25°C D

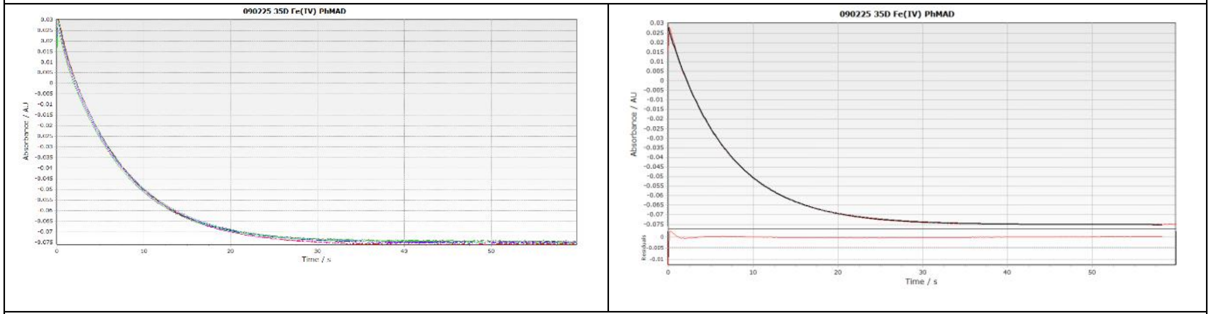

35°C D

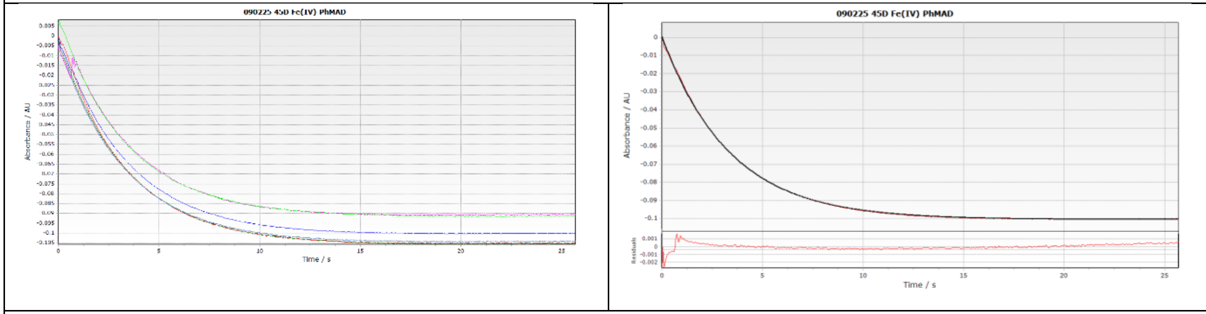

45°C D

Day 3 data (September 04, 2025)

	Pseudo-first-order rate constants k^{pfo} (s^{-1})									
**Temp (°C)**	**Trial H1**	**Trial H2**	**Trial H3**	**Trial H4**	**Trial H5**	**Trial H6**	**Average k_H^{pfo} (s^{-1})**	**Stdev**	**k_{2H} (M^{-1}s^{-1})**	**Stdev [a]**
45	1.83492	1.78375	1.82124	1.81912	1.82037	1.77975	1.81330	2.65E-02	3.63E+02	5.30E+00

35	1.26955	1.28267	1.26080	1.26446	1.27104	1.27732	1.27101	1.10E-02	2.54E+02	2.20E+00
25	0.86034	0.86316	0.85637	0.85523	0.85270	0.85101	0.85996	3.41E-03	1.72E+02	6.82E-01
15	0.46558	0.51809	0.54777	0.54864	0.54925	0.54972	0.51048	4.16E-02	1.02E+02	8.32E+00
5	0.31838	0.33159	0.32647	0.32916	0.33390	0.33072	0.32548	6.66E-03	6.51E+01	1.33E+00

**Temp (°C)**	**Trial D1**	**Trial D2**	**Trial D3**	**Trial D4**	**Trial D5**	**Trial D6**	**Average k_D^{pfo} (s-1)**	**Stdev**	**k_{2D} (M-1s-1)**	**Stdev [a]**
45	0.29152	0.29947	0.29650	0.29743	0.29248	0.29158	0.2958	4.02E-03	5.92E+01	8.03E-01
35	0.14391	0.14497	0.14545	0.14340	0.14394	0.14330	0.1448	7.88E-04	2.90E+01	1.58E-01
25	0.06832	0.07240	0.07395	0.07277	0.07308	0.07345	0.0716	2.91E-03	1.43E+01	5.82E-01
15	0.03247	0.03269	0.03274	0.03260	0.03295	0.03305	0.0326	1.44E-04	6.53E+00	2.87E-02
5	0.01598	0.01625	0.01678	0.01648	0.01699	0.01664	0.0163	4.07E-04	3.27E+00	8.14E-02

[a] = (Stdev(for k^{pfo})/k^{pfo})*k_2

Six kinetic trials and the mean fit	First-order kinetic fitted average of six trials
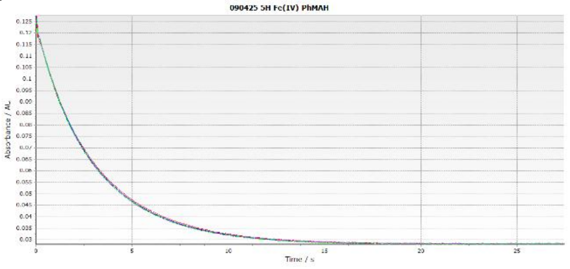	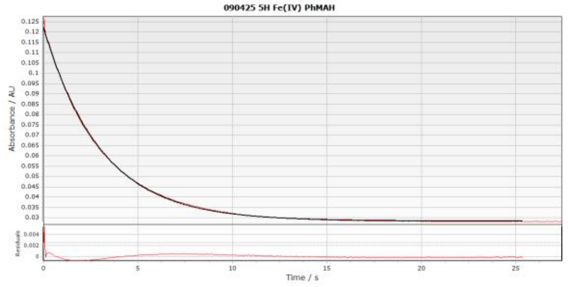
5°C H	
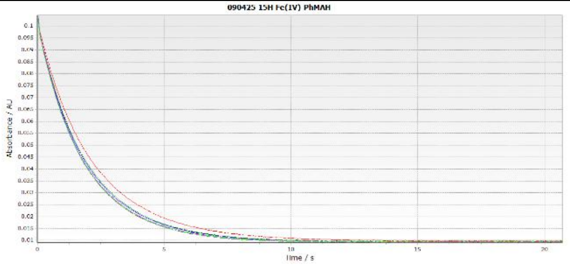	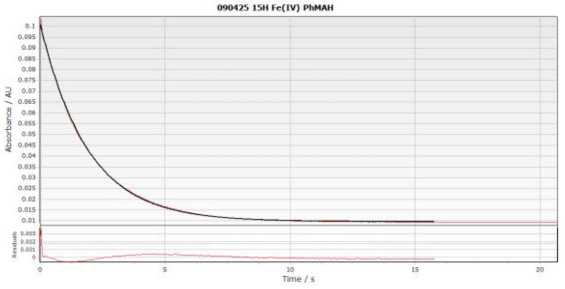
15°C H	
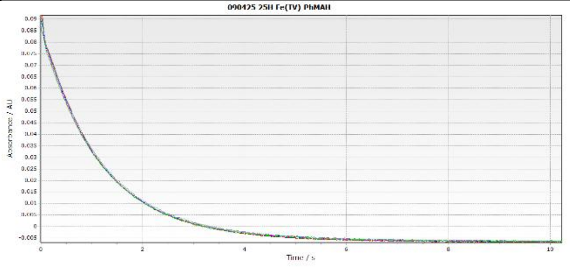	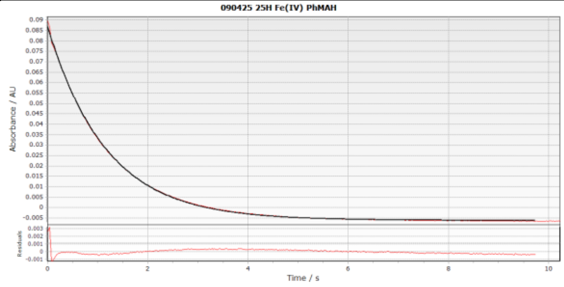
25°C H	

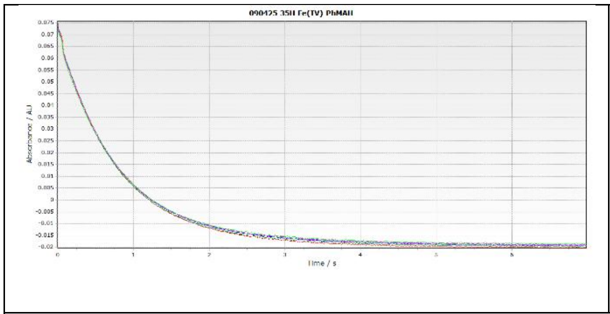

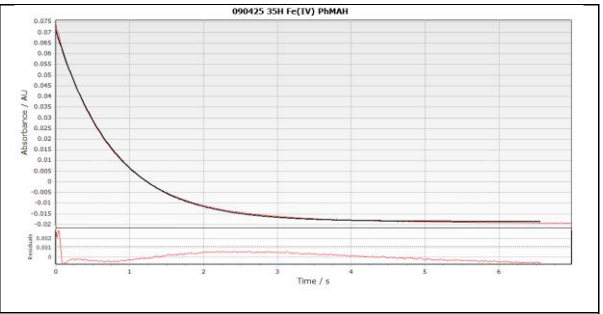

35℃ H

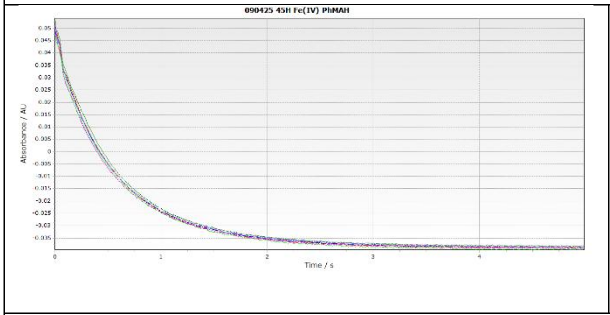

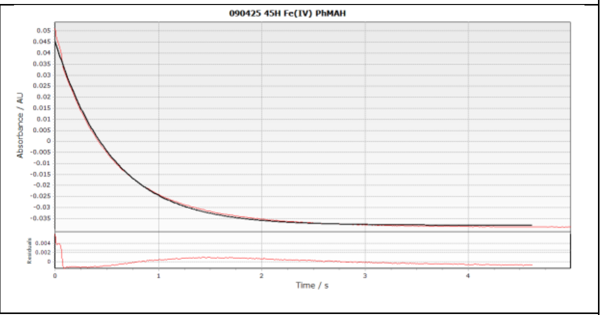

45℃ H

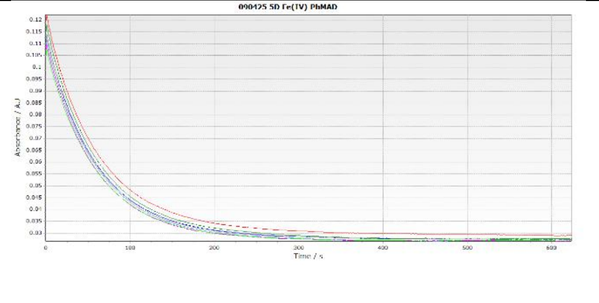

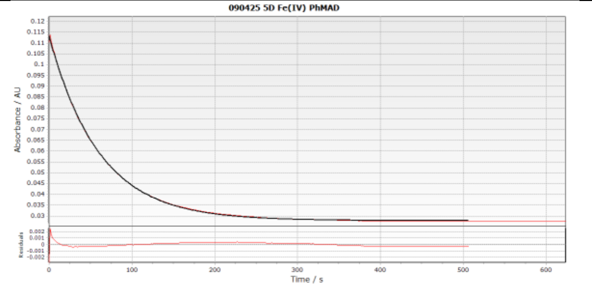

5℃ D

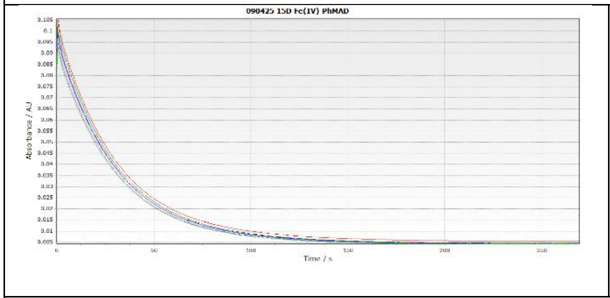

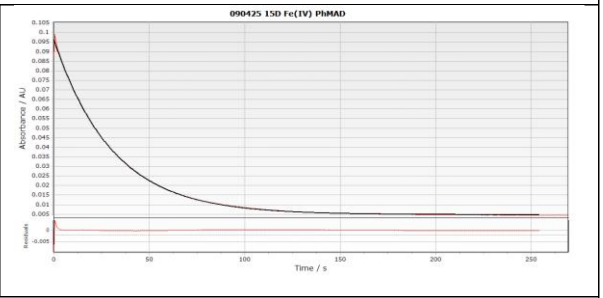

15℃ D

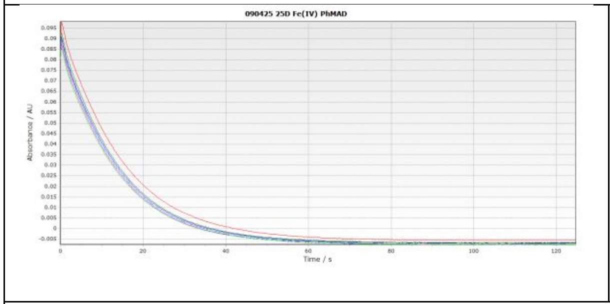

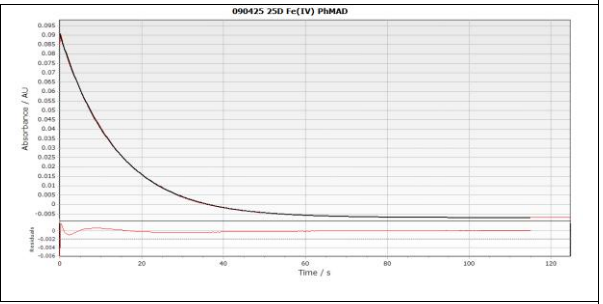

25℃ D

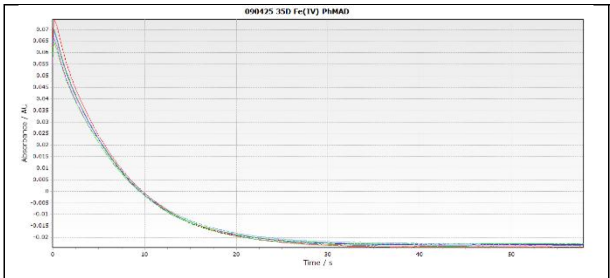
090425 35D Fe(IV) PhMAD
Absorbance / AU
Time / s

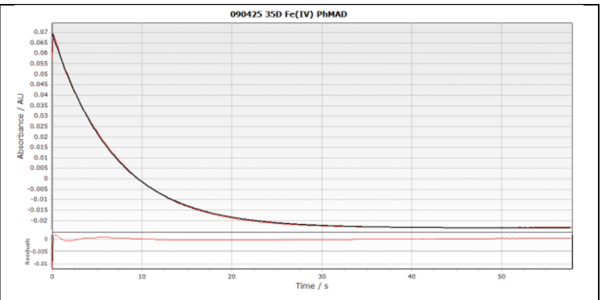
090425 35D Fe(IV) PhMAD
Absorbance / AU
Residuals
Time / s

35℃ D

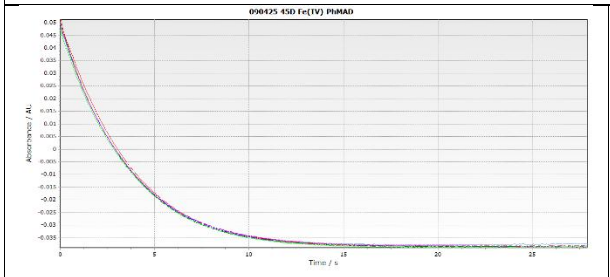
090425 45D Fe(IV) PhMAD
Absorbance / AU
Time / s

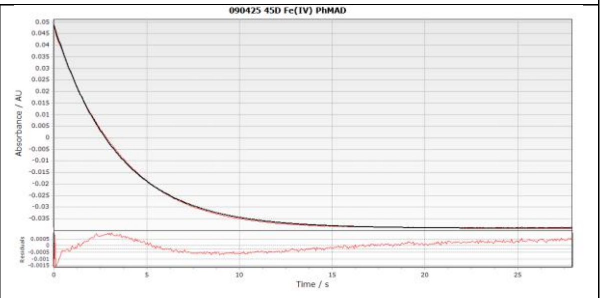
090425 45D Fe(IV) PhMAD
Absorbance / AU
Residuals
Time / s

45℃ D
